# A shape-based inter-layer contours correspondence method for ICT-based reverse engineering

**DOI:** 10.1371/journal.pone.0176383

**Published:** 2017-05-10

**Authors:** Liming Duan, Shangpeng Yang, Gui Zhang, Fei Feng, Minghui Gu

**Affiliations:** 1 ICT Research Center, Key Laboratory of Optoelectronic Technology and System of the Education Ministry of China, Chongqing University, Chongqing, China; 2 Engineering Research Center of Industrial Computed Tomography Nondestructive Testing of the Education Ministry of China, Chongqing University, Chongqing, China; 3 College of Mechanical Engineering, Chongqing University, Chongqing, China; University of California Santa Barbara, UNITED STATES

## Abstract

The correspondence of a stack of planar contours in ICT (industrial computed tomography)-based reverse engineering, a key step in surface reconstruction, is difficult when the contours or topology of the object are complex. Given the regularity of industrial parts and similarity of the inter-layer contours, a specialized shape-based inter-layer contours correspondence method for ICT-based reverse engineering was presented to solve the above problem based on the vectorized contours. In this paper, the vectorized contours extracted from the slices consist of three graphical primitives: circles, arcs and segments. First, the correspondence of the inter-layer primitives is conducted based on the characteristics of the primitives. Second, based on the corresponded primitives, the inter-layer contours correspond with each other using the proximity rules and exhaustive search. The proposed method can make full use of the shape information to handle industrial parts with complex structures. The feasibility and superiority of this method have been demonstrated via the related experiments. This method can play an instructive role in practice and provide a reference for the related research.

## Introduction

Currently, surface reconstruction from a stack of planar contours extracted from slices is a significant problem in reverse engineering. Surface reconstruction plays an important role in modeling [1, 2], and is generally divided into three sub-problems: correspondence, branch and tiling [3]. As the primary sub-problem, correspondence directly affects the efficiency and quality of surface reconstruction. Generally, the correspondence from a stack of planar contours is difficult when the contours or topology of the object are complex.

Many studies on correspondence have been conducted since the 1970s. Bersler [4] developed an approach based on domain knowledge and used Bayesian analysis to construct constraints for the correspondence. In [5], the signed distance function was used to perform the distance transformation for regional correspondence. Projection overlap-based methods use 2D bounding boxes [6] to evaluate the overlaps, and these methods were used to reconstruct intensive structures such as the plexus [7]. The minimum spanning tree method approximates the contours as ellipses, assembles them into generalized cylinders, and minimizes the spanning tree for the correspondence [3]. These above methods can obtain satisfactory results when the objects are not complex. To handle the objects with complex structures, two types of topological data have been introduced: the Reeb graph and contours tree. The Reeb graph derived from Morse theory is used to establish an inter-layer topology to guide the correspondence process [8]. In [9], as the extension of the Reeb graph, a data structure was developed to represent the non-manifold topology. Zheng [10] first set the contours radius threshold, and then used the contours tree to find inter-layer contours have been performed correspondence, which is effective for nested structures. Wang [11] applied an intelligent pattern by designing a three-layer back propagation (BP) network for the contours tree to accomplish the correspondence. Chen [12] first computed the overlap ratio of the inter-layer contours, and then combined certain rules to determine the correspondence on the contours tree. In [13], Owada enumerated all possible cases to conduct the correspondence for complex cases such as nesting and branch, but this method is not automatic, and requires manual intervention. Furthermore, it is difficult to find all cases in complex situations.

The described conventional correspondence methods are insufficient for addressing the correspondence problem of complex geometry, which refers to the inter-layer concave branch and nesting in this paper. Projection overlap-based methods are not suitable for handling concavity due to the too large error between the bounding box and the actual shape. The results from minimum spanning tree are prone to errors in the process of performing correspondence with nesting, which tends to confuse the construction of the spanning tree. Building a Reeb graph and a contour tree requires manual intervention and professional knowledge, making automation unavailable in this case. In conclusion, all above-mentioned methods are difficult for the correspondence of branch. Hence, a method to address the problem of complex correspondence is required.

In addition to the above inter-layer contours correspondence methods, there are several methods to perform correspondence of inter-layer contour-segments. Shih [14] used the similarity of the inter-layer contour-segments to perform correspondence of contour-segments with the help of the contour tree. Barequet [15] used the proximity of discrete points in inter-layer contours to vote for corresponding contour-segments [16]. However, there are few previous studies on correspondence based on inter-layer contour-segments.

Vector graphics are composed of graphical primitives that can be expressed by mathematical formulas [17, 18], so they are characterized by smaller memory when depicting contours. When rebuilding a CAD model from a series of slices, the extracted contours from the slices tend to be accurately vectorized because the shape of industrial parts is always regular. Vectorized contours are composed of several basic primitives, such as circles, arcs and segments. In addition, some algorithms have been presented to identify primitives from contours of slices [19, 20].

In this paper, considering the regularity of industrial parts and the characteristics of vector graphics, we present a shape-based inter-layer contours correspondence method. In this method, inter-layers contours are vectorized, and represented by the primitives that are considered as basic correspondence elements. We utilize the similarity and proximity to perform inter-layer contours correspondence using the contour-segments correspondence methods [21, 22]. This method is specially designed for industrial parts modeling in ICT-based reverse engineering, and can improve the correspondence performance of complex industrial parts. In our method, the vectorized contours are composed of three types of primitives: circles, arcs and segments.

This paper is organized as follows. Section 2 defines the custom terms and provides a method overview. Section 3 describes how to perform the correspondence of inter-layer primitives. In Section 4, the inter-layer contours are performed correspondence based on the results in Section 3. Section 5 presents the experiments and discussion. The conclusions are presented in Section 6.

## Custom terms and overview of the proposed method

**Fig 1:**
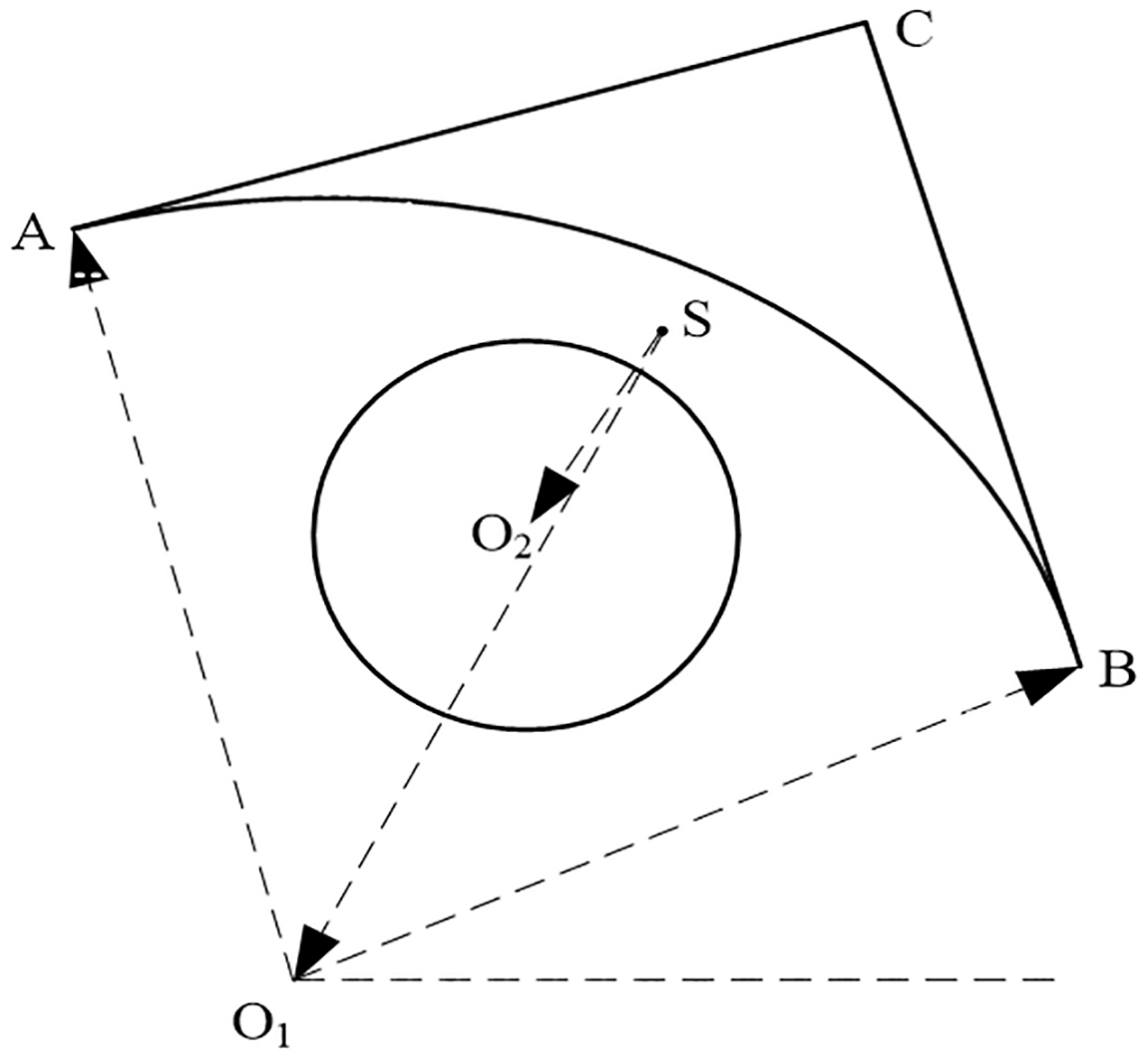
Illustration of the custom terms.

Some terms were defined in this paper to facilitate the narrative. As illustrated in Fig 1, the custom terms are defined as follows.

1. The **unsealed primitive** refers to a segment or arc, such as *AC* or $\overset{⌢}{AB}$.
2. The **composite contour** refers to the contour constructed by unsealed primitives, e.g., *ABC* is composed of *AC*, *BC* and $\overset{⌢}{AB}$.
3. The **layer center** refers to the geometric center of the composition of all contours in a layer. For example, point *S* is the layer center of the layer with ⊙*O*_2_ and composite contour *ABC*.
4. All these components constitute the vectors of **the layer center to the circle center** (LTC) by lining from the layer center to the circle or arc center, such as $\vec{SO_{2}}$ and $\vec{SO_{1}}$.

The basis of this method is contours vectorization according to the regular shape of industrial parts. In this paper, the correspondence of inter-layer contours was first considered, and then the total correspondence can be achieved by concatenating the results of inter-layer correspondence. The refined steps are described as follows.

**Step 1** Mutually perform the correspondence of inter-layer circles or arcs, and mutually perform the correspondence of the inter-layer segments.
**Step 2** Perform the pairwise correspondence of the inter-layer composite contours, and perform the pairwise correspondence of the inter-layer composite contours.
**Step 3** Perform the correspondence of the inter-layer branch contours, which includes three branch cases: one-to-two, one-to-many and many-to-many.

## Correspondence of the inter-layer primitives

The inter-layer correspondence of primitives includes two cases: inter-layer correspondence of a circle or an arc and inter-layer correspondence of a segment, which is elaborated in the following sub-sections.

### 3.1 Inter-layer correspondence of a circle or an arc

**Fig 2:**
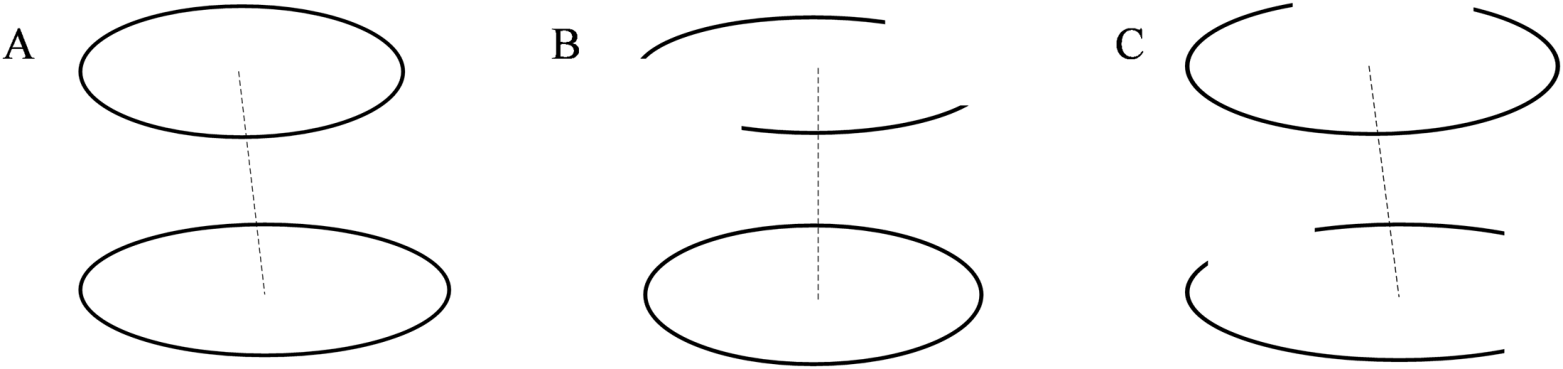
Cases of inter-layer correspondence of a circle or an arc. (A) inter-layer correspondence between a circle and a circle;(B) inter-layer correspondence between a circle and arc(s); and (C) inter-layer correspondence between arc(s) and arc(s).

As is shown in Fig 2, inter-layer correspondence of a circle or an arc includes three cases: inter-layer correspondence between a circle and a circle, inter-layer correspondence between a circle and arc(s), and inter-layer correspondence between arc(s) and arc(s).

By extracting the centerline and estimating the radius, the surface reconstruction of tubular trees provides an approximate circular shape of the contours to perform correspondence of inter-layer contours [16, 17]. However, this method do not involve the inter-layer correspondence of the arcs, and is merely applicable to the pipeline structure. Based on the above-mentioned method, we perform the correspondence using the homology of the centroids and radius of inter-layer circles or arcs.

**Fig 3:**
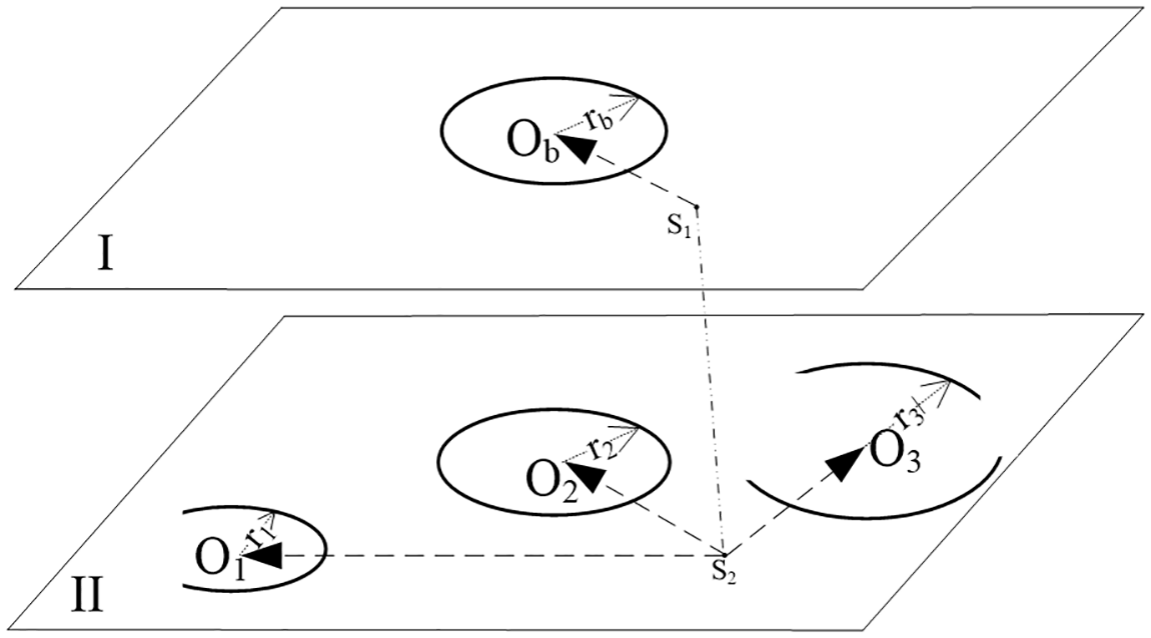
Illustration of the inter-layer correspondence of a circle or an arc.

The correspondence algorithm of an inter-layer circle or an arc is shown in Fig 3. In Fig 3, *S*_1_ and *S*_2_ are the layer centers of layers I and II, respectively. The algorithm can be implemented by the following steps.

**Step 1** According to the coincidence of the arcs centers and the equality of their radiuses, combine all appropriate arcs into a set of arcs in each layer such as *O*_3_.
**Step 2** Select a circle or a set of arcs in layer I as the reference such as *O*_*b*_, whose radius is *r*_*b*_. Traverse the circles or a set of arcs in layer II to find the circles or a set of arcs with the radius *r*_*i*_ that satisfy Formula (1).
  $$\left|r_{i}-r_{b}\right|\leq \tau_{r}$$ (1)
  In Formula (1), the threshold *τ*_*r*_ is determined by operator’s experience according to the structure, size, complexity and layer spacing of the reconstructed object, so are the thresholds *τ*_*d*_, *τ*_*θ*_, *τ*_*v*_, *τ*_*m1*_ and*τ*_*m2*_. In general, the threshold *τ*_*r*_ is set to prevent an irrational correspondence, and does not require an accurate value.
**Step 3** Construct the referenced LTC $\vec{S_{1}O_{b}}$ in layer I by lining from the layer center *S*_1_ to the circle center *O*_*b*_, and construct the LTCs ${\vec{S_{2}O}}_{1}$, $\vec{S_{2}O_{2}}$ and $\vec{S_{2}O_{3}}$ in layer II. Then, seek the closest LTC in layer II to the referenced LTC according to the vector-close rule (VCR). The circle or a set of arcs that maps to the closest vector is required.

**Fig 4:**
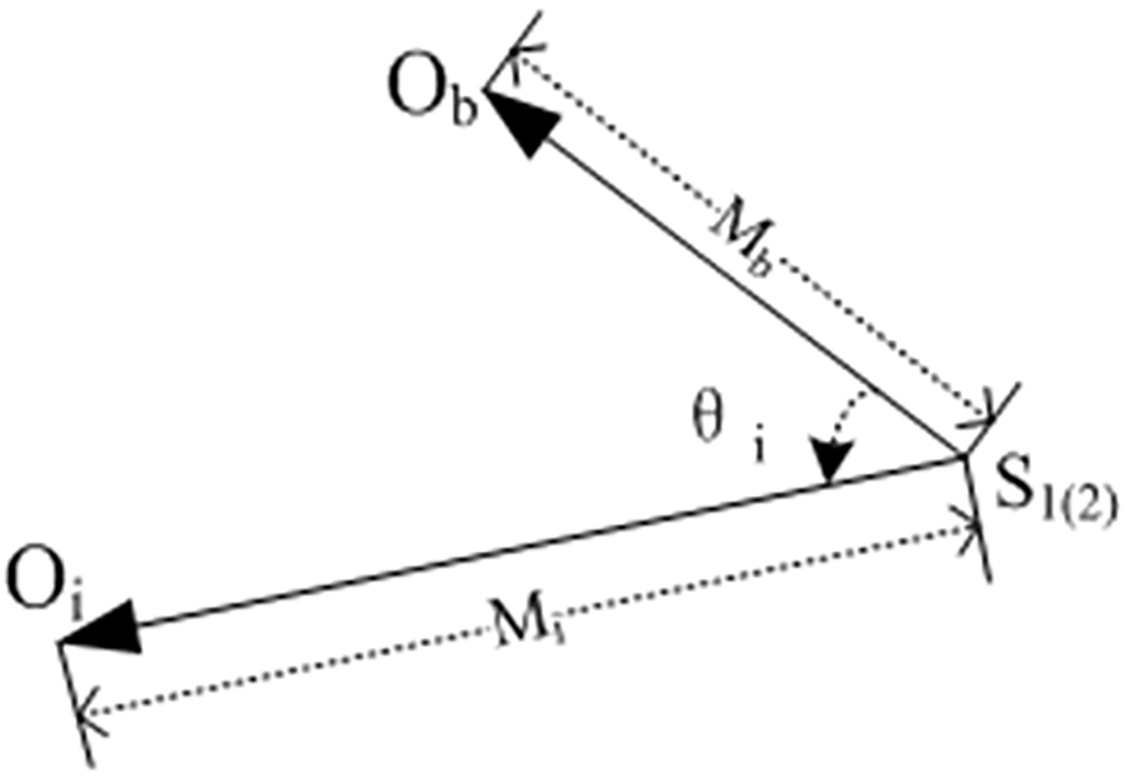
Illustration of the VCR.

The VCR is shown in Fig 4, where *M*_*b*_ is the norm of the referenced LTC, *M*_*i*_ (*i* = 0, 1…*m*) is the norm of the *i*th LTC in layer II, and *θ*_*i*_ (*i* = 0, 1…*m*) is the angle from *M*_*b*_ to *M*_*i*_. The mathematical expression of VCR is Formula (2). By combining the mutual difference of their norms with the proximity of angle *θ*_*i*_ to zero, the VCR evaluates the smallestΔ_*i*_ among the Δ_*i*_ s, and is labeled as Δ_*m*_. Each of Δ_*i*_ expresses the proximity of two vectors. In Formula (3), all the circles or a set of arcs that corresponding to Δ_*m*_ are required in layer II. Note that in Formula (2), Φ represents no results.

$$\Delta_{i}=\{\begin{array}{cc} \left|\frac{M_{i}}{M_{b}}-1\right|\cdot \left|\left|\cos\theta_{i}\right|-1\right| & \left(M_{b}\geq M_{i}\right)\;and\;\left(\frac{M_{i}}{M_{b}}\geq \tau_{m1}\right)\;and\;\left(\theta_{i}<\tau_{\theta }\right)\\ \left|\frac{M_{b}}{M_{i}}-1\right|\cdot \left|\left|\cos\theta_{i}\right|-1\right| & \left(M_{b}<M_{i}\right)\;and\;\left(\frac{M_{i}}{M_{b}}\leq \tau_{m2}\right)\;and\;\left(\theta_{i}\leq \tau_{\theta }\right)\\ \Phi & else \end{array}$$ (2)

$$\Delta_{m}=\overset{m}{\underset{i=1}{\text{min}}}\Delta_{i}$$ (3)

**Fig 5:**
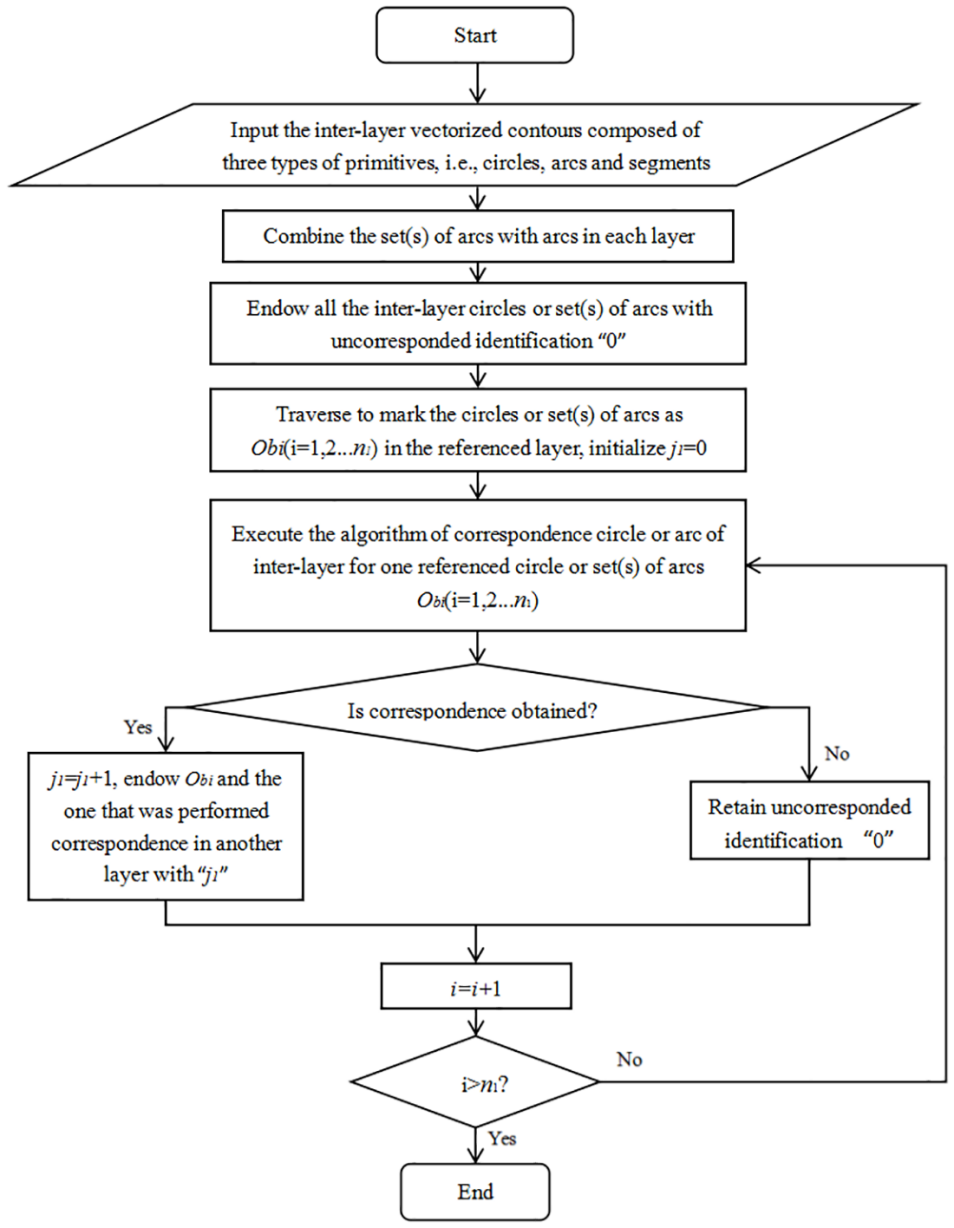
Flow chart of performing correspondence of inter-layer circles or arcs.

The above is the correspondence algorithm for a referenced circle or a set of arcs. However, in practice, there are often several circles or sets of arcs in each layer, and thus as shown in Fig 5, we designed the implementation of the algorithm (refer to Fig 3 when reading Fig 5). In Fig 5, *n*_1_ is the number of circles or sets of arcs in the referenced layer. Among the implemented results, there may be circles or sets of arcs with un-corresponded identification “0” in addition to *p*_1_ (*p*_1≤_*n*_1_) couples of corresponded circles or sets of arcs, which are processed in Section 4.

### 3.2 Correspondence of inter-layer segments

**Fig 6:**
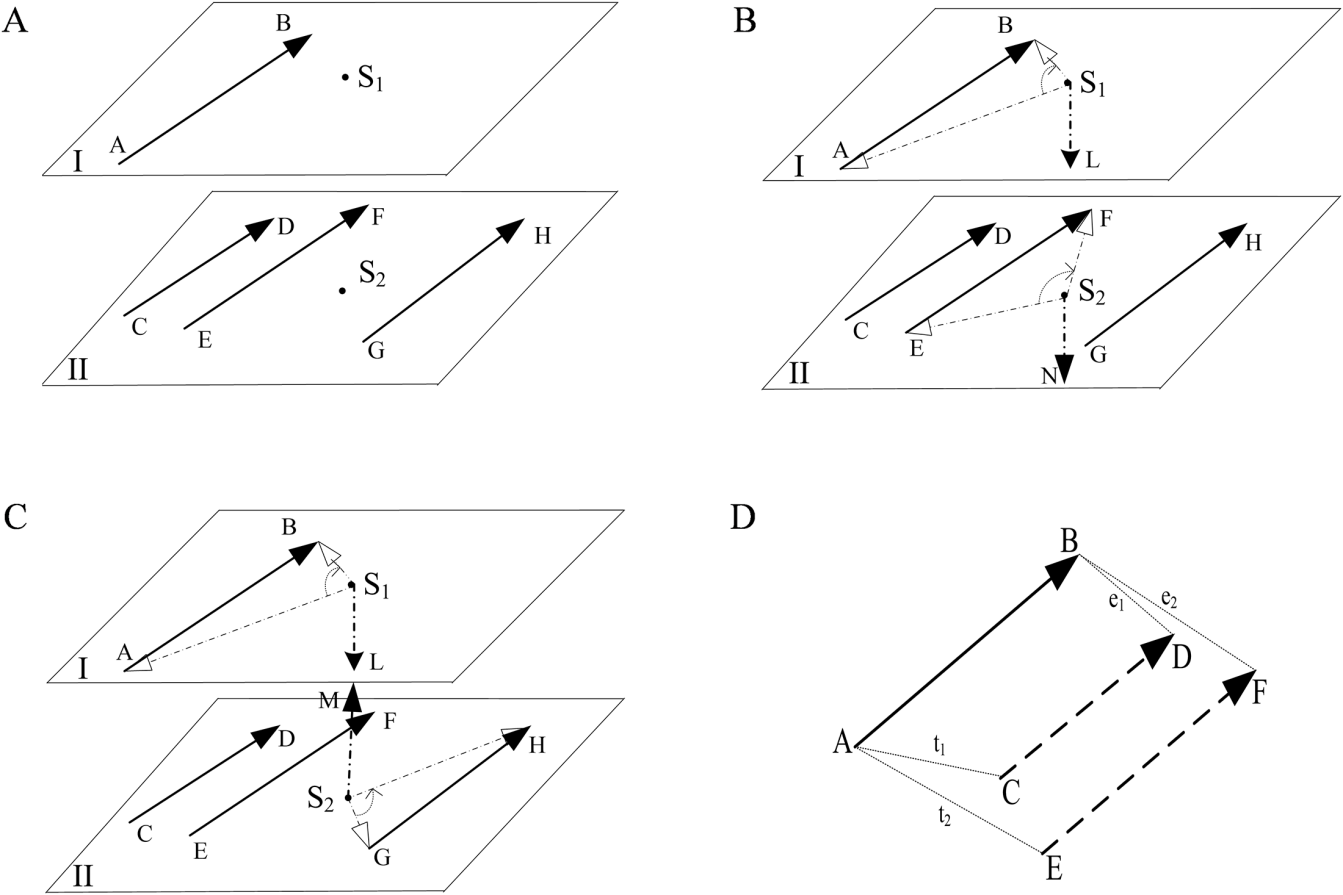
Illustration of the correspondence of inter-layer segments. (A) **VCR using in segments correspondence**; (B) diagram 1 of the SSLCR; (C) diagram 2 of the SSLCR; (D) diagram of the TCR.

The correspondence of inter-layer segments has been discussed in references [14, 22]. The steps of the method are described as follows. First, connect the sharp discrete points of the inter-layer contours in pairs by using the maximum inner product and determining the projection distance of the points. Second, consider these connections as a base to link other points. A different method was presented in this paper, which uses the spatial position of the segments to perform the correspondence. As shown in Fig 6A, the new method finds the segments that have completed correspondence in layer II for the referenced segment *AB* in layer I.

It is not difficult to imagine that the two inter-layer segments are parallel or approximately parallel. Besides, the projected positions of their appropriate endpoints on one layer are as close as possible. Based on the above consideration, three rules are used to perform inter-layer segments correspondence: VCR, the rule of the same side of the layer center (SSLCR) and the terminal close rule (TCR).

#### 3.2.1 Segments correspondence through VCR

First, construct the vectors by following the direction from left to right for all the segments in each layer, e.g., $\vec{AB}$, $\vec{CD}$, $\vec{EF}$ and $\vec{GH}$ in Fig 6(A). Second, use the VCR to find the closest vector in layer II to the referenced segment $\vec{AB}$ in layer I. However, the VCR is not a sufficient condition to perform inter-layer segments correspondence. Thus, the other two rules are appended to improve the correspondence accuracy.

#### 3.2.2 SSLCR

The purpose of the SSLCR is to examine whether the relative orientation of the vectors in layer II to the layer center *S*_2_ is consistent with that of the referenced segment $\vec{AB}$ to the layer center *S*_1_. In other words, the SSLCR determines whether both vectors are located left or right of their respective layer centers.

Referring to Fig 6B and 6C, the SSLCR is implemented as follows.

**Step 1** In layer I, connect *S*_1_ to the starting point and ending point of vector $\vec{AB}$ to construct two vectors $\vec{S_{1}A}$ and $\vec{S_{1}B}$, respectively. Use the right-hand rule to determine the orientation of the cross product $\vec{S_{1}L}=\vec{S_{1}A}\times \vec{S_{1}B}$. The orientation of $\vec{S_{1}L}$ is vertically downward.
**Step 2** Similarly, derive the cross product for each segment vector in layer II, e.g., $\vec{S_{2}N}$ for $\vec{EF}$ and $\vec{S_{2}M}$ for $\vec{GH}$.
**Step 3** Compare the orientation of each segment cross product in layer II with that of the referenced vector $\vec{S_{1}L}$, and determine the following: referring to Fig 6B, if two orientations are consistent (such as $\vec{S_{2}N}$ and $\vec{S_{1}L}$), perform the correspondence of segment *EF* with the referenced segment *AB*. Referring to Fig 6(C), if two orientation are not consistent (such as $\vec{S_{2}M}$ and $\vec{S_{1}L}$), do not perform this process (such as *GH* with *AB*).

#### 3.2.3 TCR

By comparing the distances between the appropriate ends of the referenced segment in the layer I and each segment in the layer II, TCR estimates the proximity between the referenced segment and each segment of the layer II. Referring to Fig 6D, the implementation of the TCR is presented as follows.

**Step 1** Project the referenced vector *AB* in layer I onto layer II to compare it with each vector in layer II.
**Step 2** Connect the starting and end points of the projected vector and those of each vector in layer II. Connect the other ends of the two vectors to obtain the two segments *t*_*i*_ and *e*_*i*_, and then compare the lengths of *t*_*i*_ and *e*_*i*_.
**Step 3** Traverse each vector in layer II to obtain a series of larger segments.
**Step 4** Select a minimum segment Ω from these larger ones, and compare Ω with the threshold *τ*_*d*_. If Ω≤*τ*_*d*_, obtain the result of Ω. Otherwise, there are no results. The segment in layer I that can perform correspondence with Ω is the required one.

There is a certain order when these three rules are applied. First, operate the SSLCR to determine which segments in layer II are on the same side as the referenced segment in layer I. Second, iteratively operate the VCR three times on the segments obtained by SSLCR to get the three closest segments to the referenced segment. Finally, operate the TCR on the three segments to obtain the unique segment that can perform correspondence with the referenced segment.

## Correspondence of inter-layer contours

Correspondence of inter-layer contours includes two cases: pairwise contours correspondence and branch correspondence.

### 4.1 Pairwise contours correspondence

**Fig 7:**
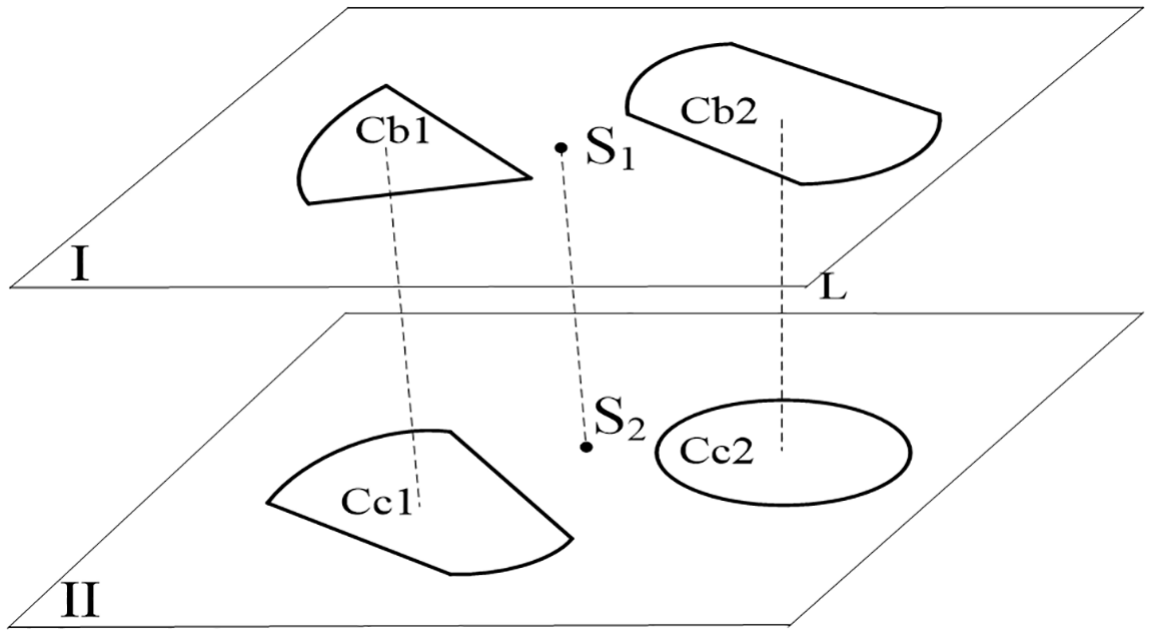
Illustration of the pairwise inter-layer contour correspondence.

As shown in Fig 7, the pairwise contour correspondence contains two cases: composite contours correspondence and correspondence of composite contour with a circle.

#### 4.1.1 Correspondence of pairwise composite contour

The procedures of pairwise composite contour correspondence are displayed as follows.

**Step 1** Randomly select a composite contour *Cb*1 in the referenced layer I, and there are *n*_*b*_ corresponded primitives in *Cb*1.
**Step 2** Traverse *n*_*b*_ primitives to check whether all of the primitives in layer II correspond with the *n*_*b*_ primitives located in one contour such as *Cc*1. If so, the two contours *Cb*1 and *Cc*1 are corresponded. Otherwise, they are not.
**Step 3** Traverse the other composite contours in the referenced layer I to perform the above check.

#### 4.1.2 Correspondence of a composite contour with a circle

To perform correspondence of a composite contour with a circle, we must calculate the angle *θ* between vectors $\vec{a}$ and $\vec{b}$, which is expressed in Formula (3).

$$\theta =\text{arccos}\left(\frac{\vec{a}\cdot \vec{b}}{\left|\vec{a}\right|\cdot \left|\vec{b}\right|}\right)\leq \tau_{v}$$ (4)

If the angle *θ* between the two vectors satisfies Formula (4), the vectors are considered parallel. Fig 7 shows the process of performing correspondence of a composite contour with a circle.

**Step 1** Select a circle (a composite contour) *Cb*_2_ in reference layer I, and select a composite contour (a circle) *Cc*_2_ in layer II.
**Step 2** Connect the centroids of *Cb*_2_ and *Cc*_2_ to construct a vector *L*, and connect two layer centers to construct vector $\vec{S_{1}S_{2}}$.
**Step 3** Evaluate Formula (4) for vectors *L* and $\vec{S_{1}S_{2}}$ to determine whether the two vectors are parallel, and if they are parallel, the circle and composite contour can be performed correspondence.
**Step 4** Traverse the composite contours (circles) in layer II to execute steps 2 and 3.
**Step 5** Traverse the circles (composite contours) in layer I to execute steps 2, 3 and 4.

### 4.2 Branch correspondence

Three branch cases are considered in this paper: one-to-two, one-to-many and many-to-many. The targeted contours in this section are the un-corresponded contours in the previous sections, which are integrated as set *Q*. In addition to the exhaustive search in the pairwise correspondence of composite contours, we use the vectors parallelism to accomplish the branch correspondence.

**Fig 8:**
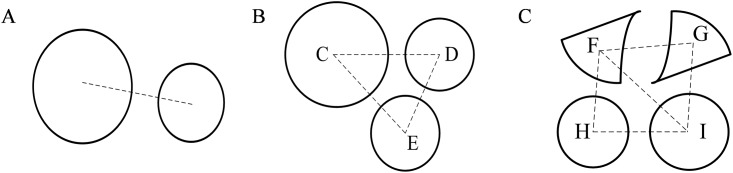
Illustration of SC. (a) TWT; (b) THT; (c) MAT.

The implementation of branch correspondence is as follows:

**Step 1** Traverse the contours of set *Q* in reference layer I to construct all possible TWTs, and calculate their centroids.
**Step 2** Randomly select a contour from set *Q* in layer II. Connect the centroid of the selected contour to each centroid of the constructed TWTs and obtain a vector.
**Step 3** Use Formula (4) on $\vec{S_{1}S_{2}}$ and the obtained vector to test whether the two vectors are parallel. If they are parallel, the TWT and selected contour in layer II form a one-to-two branch. Otherwise, split the TWT into two original contours.
**Step 4** Traverse the contours of set *Q* in layer II to execute the test. With the difference of creating SCs that are illustrated in Fig 8, construct one-to-many and many-to-many branches using method similar to that for the one-to-two branch construction.

## Experimental results and discussion

The proposed method was implemented in VC++6.0 with OpenGL. The experiments were performed on a Windows 8 PC with a 2.60 GHz Intel Celeron CPU and 2.0 GB RAM.

**Fig 9:**
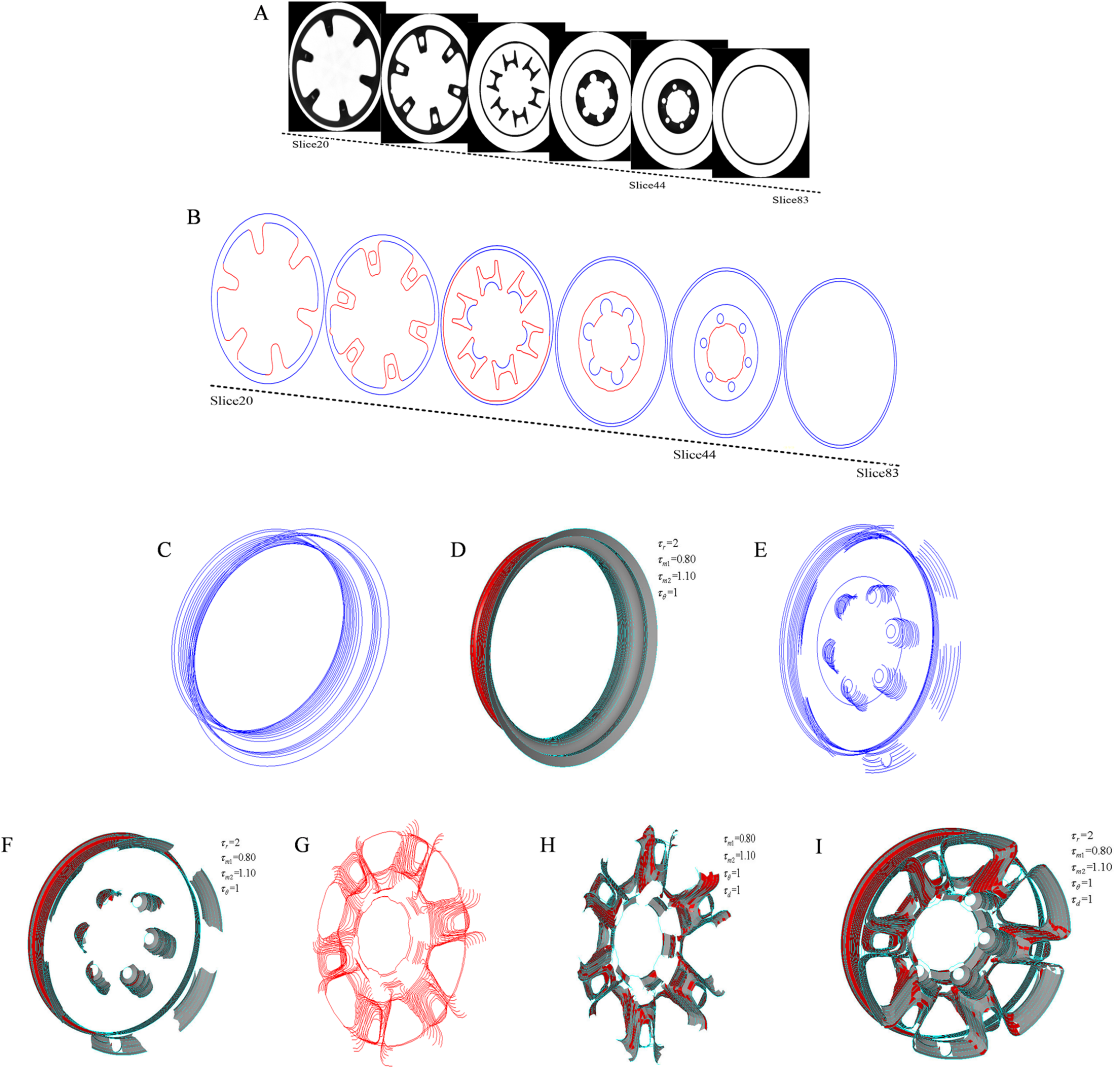
Raw data and correspondence of the hub. (A) 20~83 slices; (B) vectorized contours of the slices; (C) outer contours; (D) correspondence of the outer contours; (E) circles or arcs of the inner contours; (F) correspondence of circles or arcs of the inner contours; (G) segments of the inner contours; (H) correspondence of the segments of the inner contours; and (I) correspondence of the inner contours.

Fig 9A shows 20~83 slices of a hub scanned by ICT, and Fig 9B shows the extracted vectorized contours from the slices. In Fig 9B and similar figures, the circles and arcs are rendered in blue, and the segments are rendered in red. Fig 9C and 9D show the correspondence of the outer contours, all of which are circles. Fig 9E and 9F show the circles or arcs correspondence of the inner contours. Fig 9G and 9H show the segments correspondence of the inner contours. Fig 9I shows the inner contours correspondence according to the primitives correspondence. In Fig 9D, 9F, 9H and 9I and similar figures, the inter-layer surfaces are constructed by auto-lofting according to the correspondence of primitives and contours.

**Fig 10:**
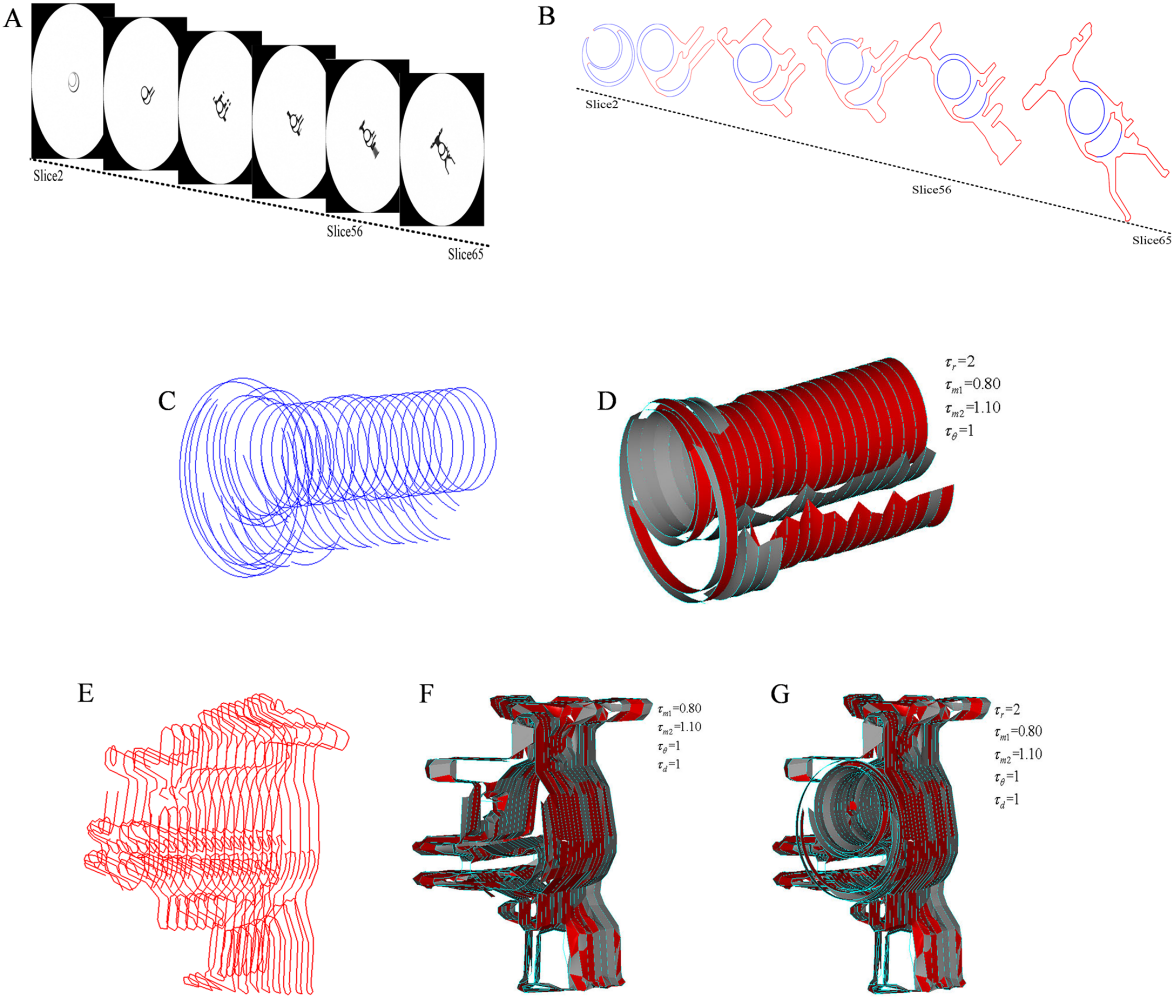
Raw data and correspondence of a carburetor. (A) 2~65 slices; (B) vectorized contours of the slices; (C) circles or arcs of the contours; (D) correspondence of the circles or arcs; (E) segments of the contours; (F) correspondence of the segments; (G) correspondence of the contours.

Fig 10A shows 2~65 slices of a carburetor scanned by ICT, and Fig 10B shows the extracted vectorized contours from the slices. Fig 10C and 10D show the correspondence of the circles or arcs of the contours. Fig 10E and 10F show the segments correspondence of the contours. Fig 10G shows the contours correspondence.

In operation, the correspondence of a circle or an arc must set *τ*_*r*_, *τ*_*m1*_, *τ*_*m2*_ and *τ*_*θ*_, the correspondence of segments must set *τ*_*m1*_, *τ*_*m2*_, *τ*_*θ*_ and *τ*_*d*_, and the correspondence of branches must also set *τ*_*v*_. Among these parameters, *τ*_*r*_ and *τ*_*d*_ take pixel as unit, *τ*_*θ*_ and *τ*_*v*_ take degrees (°) as unit, and *τ*_*m1*_ and *τ*_*m2*_ are dimensionless because they are proportions. In practice, the operation may be automated using a table look-up in programming. According to layer spacing, the radius of circles or arcs, and the statistics of segments and vectors, a mathematical table may be built to find to set the appropriate parameters. It is not difficult to obtain the best correspondence effect on any object to build the mathematical table, because we use pixels instead of the actual size. Thus, the difficulty of the operation greatly reduces, and the operators can be inexperienced.

**Fig 11:**
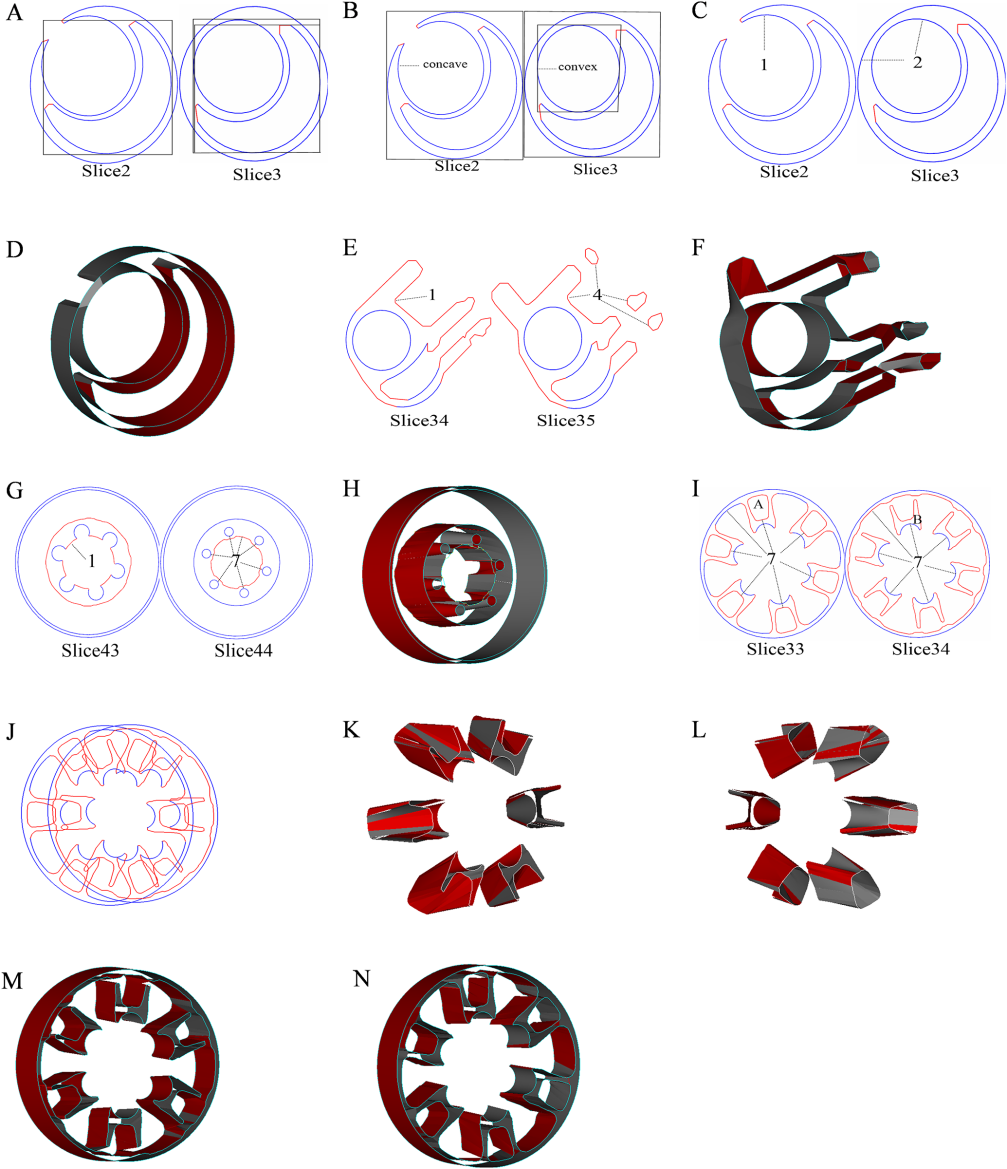
Illustration of the inter-layer pairwise and branch correspondence. (A)(B) illustrated examples of the projection overlap-based method; (C) 2–3 vectorized contours of the carburetor; (D) correspondence of the 2–3 contours of the carburetor using our method; (E) 34–35 vectorized contours of the carburetor; (F) correspondence of the 34–35 contours of the carburetor using our method; (G) 43–44 vectorized contours of the hub; (H) correspondence of the 43–44 contours of the hub using our method; (I) 33–34 vectorized contours of the hub; (J) 33–34 stacked contours of hub; (K)(L) false correspondence by minimum spanning tree; (M)(N) correspondence of the 33–34 contours of hub using our method.

According to the projection overlap-based method in Fig 11A, because of the concave shape of the crescent, the significant closeness is observed between box in slice 2 of the bounding crescent and the two boxes in slice 3 of the bounding crescent and a combination of the crescent and circle. Thus, there is great uncertainty in the process of the correspondence of the crescent of slice 2 with the crescent of slice 3. For the branch correspondence in Fig 11B using the projection overlap-based method, the one-to-two branch cannot be identified by the outermost boxes in slice 2 and slice 3 due to the nesting and concave shape. However,our method is applicable in convex and concave shapes,and provides better results for both shapes. As a convex example of the pairwise correspondence, the central circles in the 34–35 contours of the carburetor are handled well using our method, which are shown in Fig 11E and 11F. As a concave example of pairwise correspondence, the crescents on the bottom right in the 2–3 contours of the carburetor are effectively performed correspondence using our method, which are shown in Fig 11C and 11D. This advantage of the method extends from pairwise to branch such as one-to-two in Fig 11C and 11D, one-to-four in Fig 11E and 11F and one-to-seven in Fig 11G and 11H. The processing capacity of minimum spanning tree for nesting is poor, so the correspondence results in the complex case may be unreliable or erroneous, which are shown in Fig 11I–11L. In the above figures, the two closed contours A and B are not correctly performed correspondence, with each other. However, as shown in Fig 11I, 11J, 11M and 11N, our method can freely handle nesting, which also shows the seven-to-seven branch. In addition, our method is superior to the methods based on the Reeb graph and contour tree due to its easy implementation and automation capability.

In summary, in addition to a good pairwise correspondence of inter-layer contours, the method proposed in this paper can perform the inter-layer branch correspondence better than conventional methods, which includes both one-to-two and one-to-many cases as well as the more complex case of many-to-many. Moreover, for other complex cases such as nesting and a mixture of convex and concave, our method can perform perfect correspondence, which is difficult for conventional methods to achieve.

The correspondence method presented in this paper is based on the inter-layer primitives correspondence, which can be achieved in cases with pairwise and complex branch and nesting.

Inevitably, this method has a shortcoming. Because this method requires the recognized primitives to accurately express the original shape of the contours, poor correspondence may occur when there are few types of primitives in the object with irregular shape. However, despite this shortcoming, the proposed method has certain applications in ICT-based reverse engineering for its special ability to handle complex industrial parts with regular shapes.

## Conclusions

In this paper, a novel shape-based inter-layer contours correspondence method was proposed for surface reconstruction in ICT-based reverse engineering. This method is customized for industrial parts due to the regularity of the shape of the extracted vectorized contours from ICT-scanned slices. This method is simple, straightforward, easy to implement, and able to deal with various structures from simple pairwise to complex branch and nesting with either concave or convex contours. Compared with the conventional methods, this method has unparalleled advantages in the aspect of handling industrial parts with complex structures.

This method provides a new direction for studying correspondence in ICT-based reverse engineering, which can play an instructive role in practice and provide a reference for the related research. Moreover, the shape-based idea in this method may be adopted in other related fields.

This method is mainly targeted at the regular industrial parts. Therefore, the effect is not good for the irregular contours that cannot be accurately vectorized. This shortcoming provides an opportunity for improving this method. The emergence of more types of primitives such as ellipses, parabolas, hyperbolas, involutes, B-splines and Bézier curves is expected to improve the vectorization efficiency and quality. Because these primitives may be more accurately fit for the irregular contours, this method may be improved by adding these primitives.

## Supporting information

S1 File Raw image data. (ZIP)

S2 File Final image data. (ZIP)
