# Supplementary figures and images for: A shape-based inter-layer contours correspondence method for ICT-based reverse engineering (part 2 of 2)
Source: PLoS One. 2017 May 10;12(5):e0176383. doi: 10.1371/journal.pone.0176383 (PMC5425182; doi:10.1371/journal.pone.0176383)

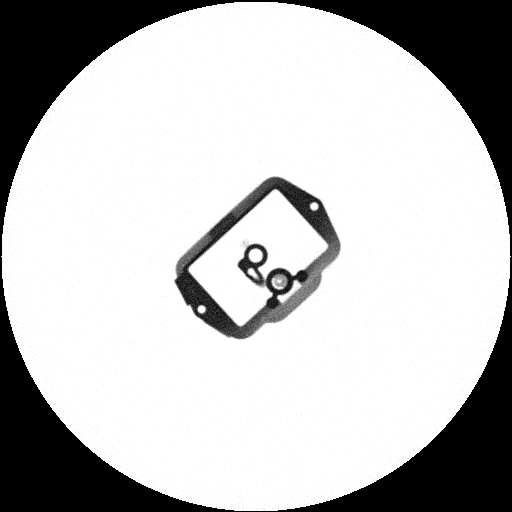

Supplement: S1 File — (ZIP) [file pone.0176383.s001.zip › Raw Image Data of a carburetor/CC-128.bmp]

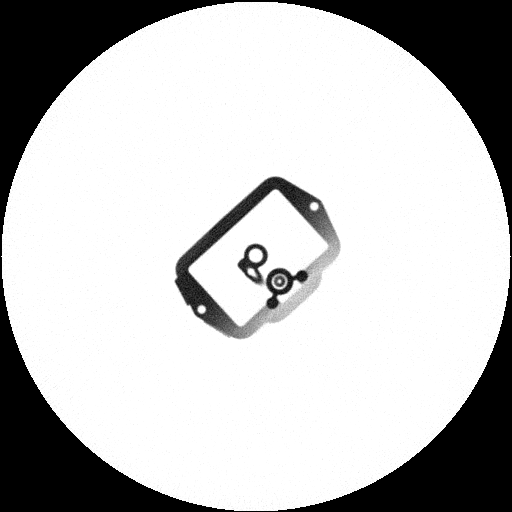

Supplement: S1 File — (ZIP) [file pone.0176383.s001.zip › Raw Image Data of a carburetor/CC-129.bmp]

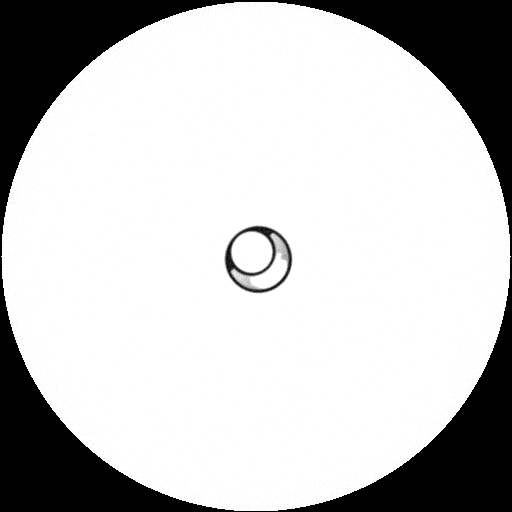

Supplement: S1 File — (ZIP) [file pone.0176383.s001.zip › Raw Image Data of a carburetor/CC-13.bmp]

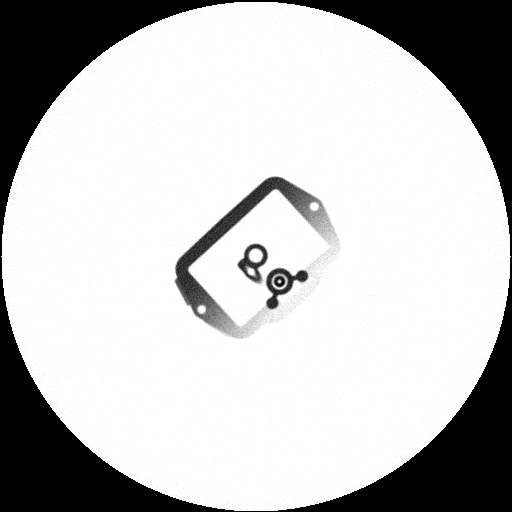

Supplement: S1 File — (ZIP) [file pone.0176383.s001.zip › Raw Image Data of a carburetor/CC-130.bmp]

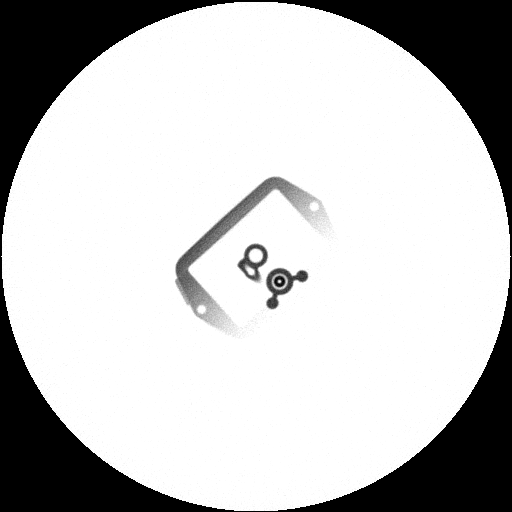

Supplement: S1 File — (ZIP) [file pone.0176383.s001.zip › Raw Image Data of a carburetor/CC-131.bmp]

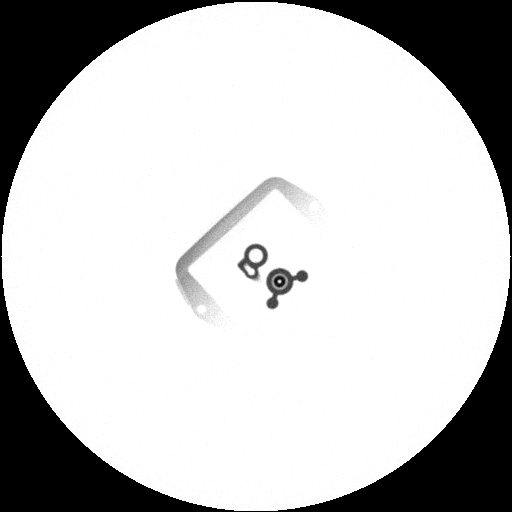

Supplement: S1 File — (ZIP) [file pone.0176383.s001.zip › Raw Image Data of a carburetor/CC-132.bmp]

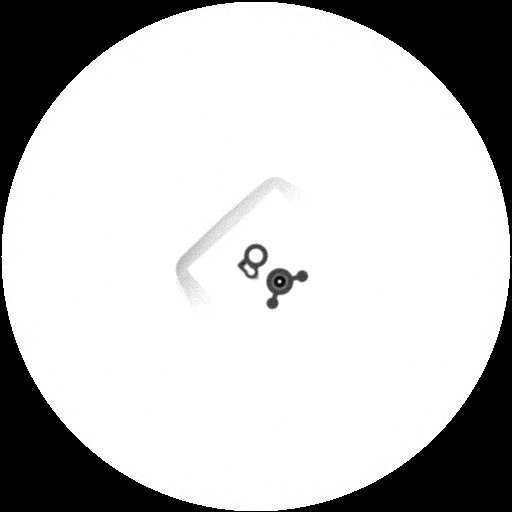

Supplement: S1 File — (ZIP) [file pone.0176383.s001.zip › Raw Image Data of a carburetor/CC-133.bmp]

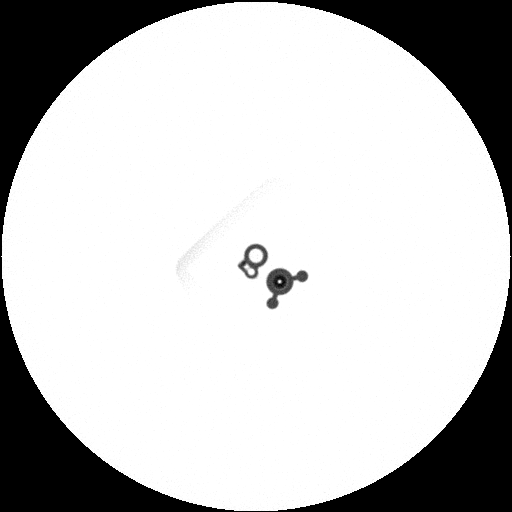

Supplement: S1 File — (ZIP) [file pone.0176383.s001.zip › Raw Image Data of a carburetor/CC-134.bmp]

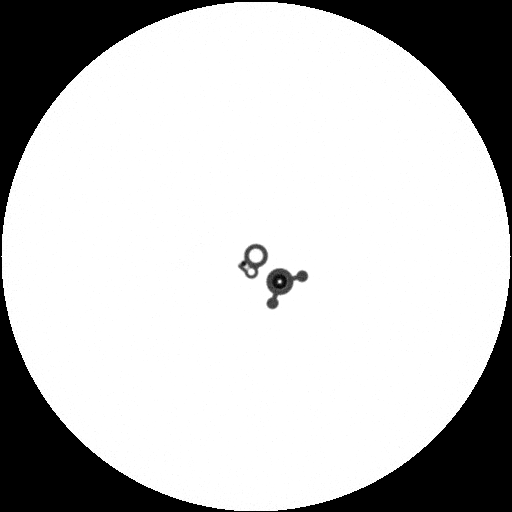

Supplement: S1 File — (ZIP) [file pone.0176383.s001.zip › Raw Image Data of a carburetor/CC-135.bmp]

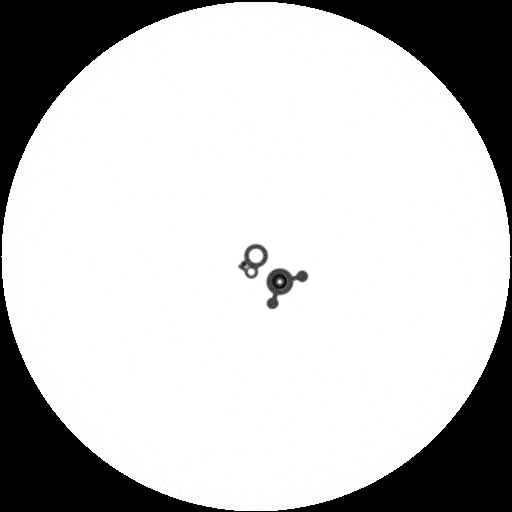

Supplement: S1 File — (ZIP) [file pone.0176383.s001.zip › Raw Image Data of a carburetor/CC-136.bmp]

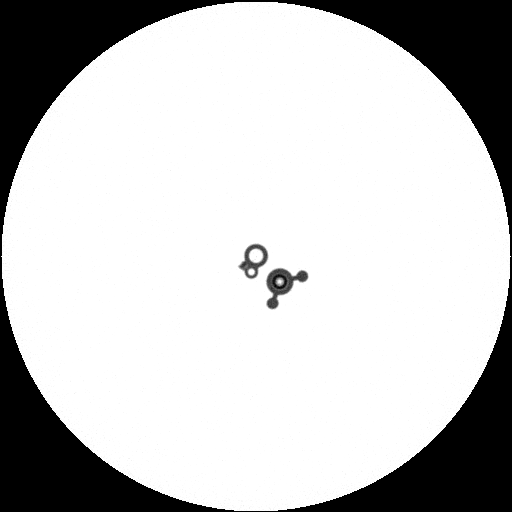

Supplement: S1 File — (ZIP) [file pone.0176383.s001.zip › Raw Image Data of a carburetor/CC-137.bmp]

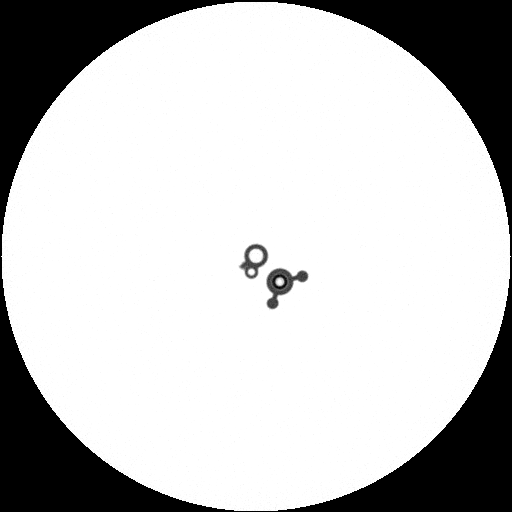

Supplement: S1 File — (ZIP) [file pone.0176383.s001.zip › Raw Image Data of a carburetor/CC-138.bmp]

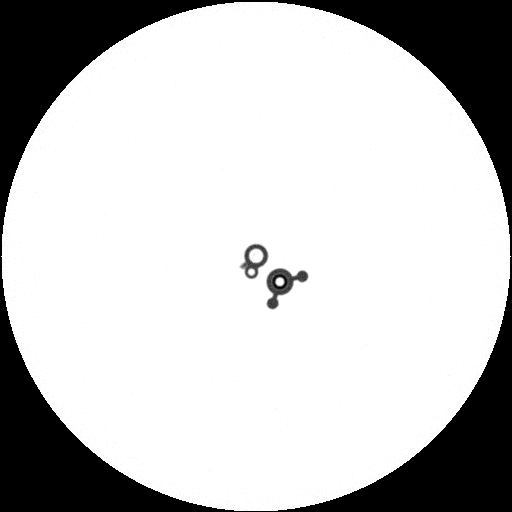

Supplement: S1 File — (ZIP) [file pone.0176383.s001.zip › Raw Image Data of a carburetor/CC-139.bmp]

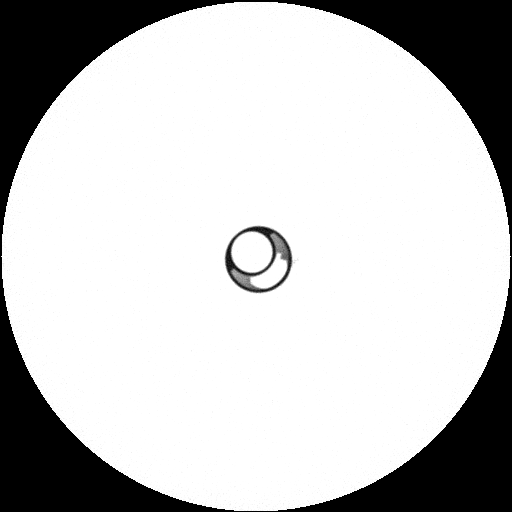

Supplement: S1 File — (ZIP) [file pone.0176383.s001.zip › Raw Image Data of a carburetor/CC-14.bmp]

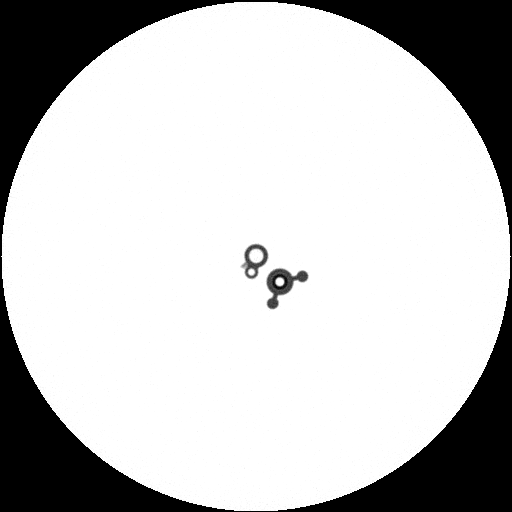

Supplement: S1 File — (ZIP) [file pone.0176383.s001.zip › Raw Image Data of a carburetor/CC-140.bmp]

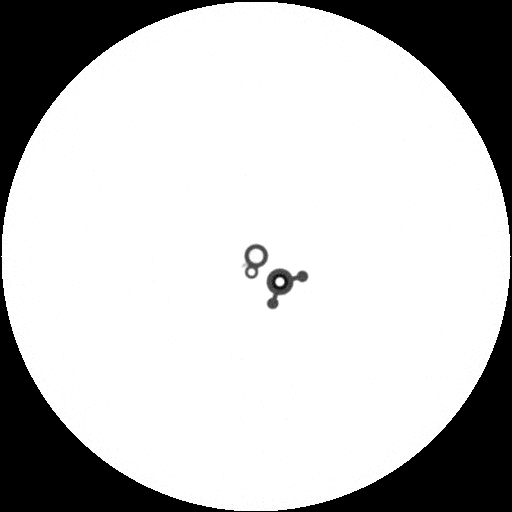

Supplement: S1 File — (ZIP) [file pone.0176383.s001.zip › Raw Image Data of a carburetor/CC-141.bmp]

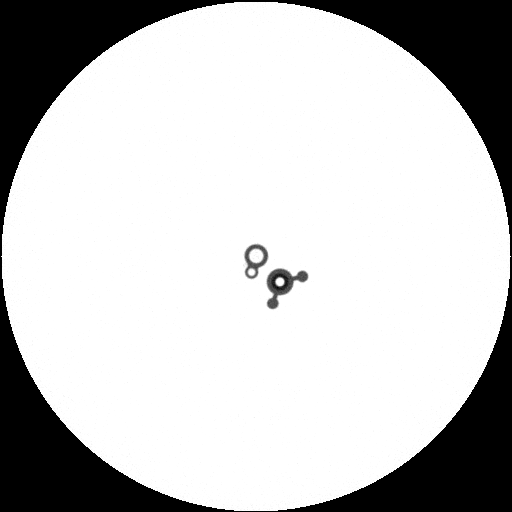

Supplement: S1 File — (ZIP) [file pone.0176383.s001.zip › Raw Image Data of a carburetor/CC-142.bmp]

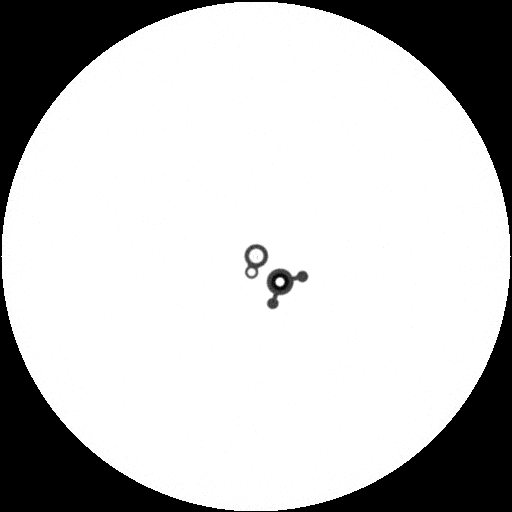

Supplement: S1 File — (ZIP) [file pone.0176383.s001.zip › Raw Image Data of a carburetor/CC-143.bmp]

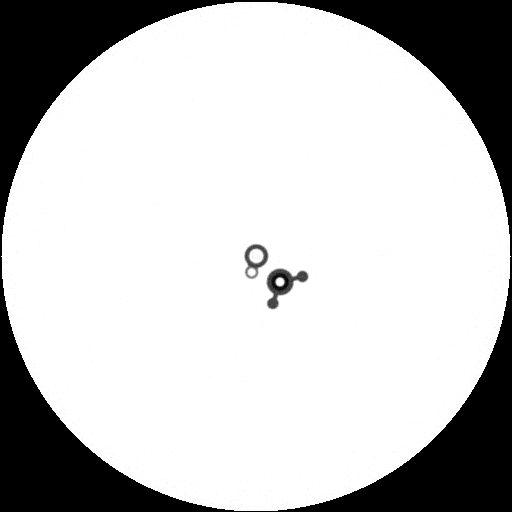

Supplement: S1 File — (ZIP) [file pone.0176383.s001.zip › Raw Image Data of a carburetor/CC-144.bmp]

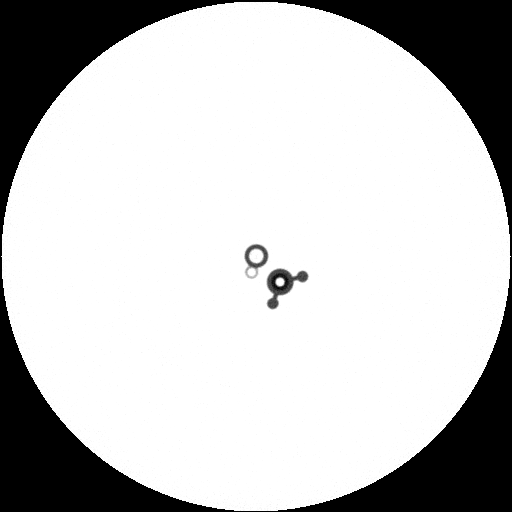

Supplement: S1 File — (ZIP) [file pone.0176383.s001.zip › Raw Image Data of a carburetor/CC-145.bmp]

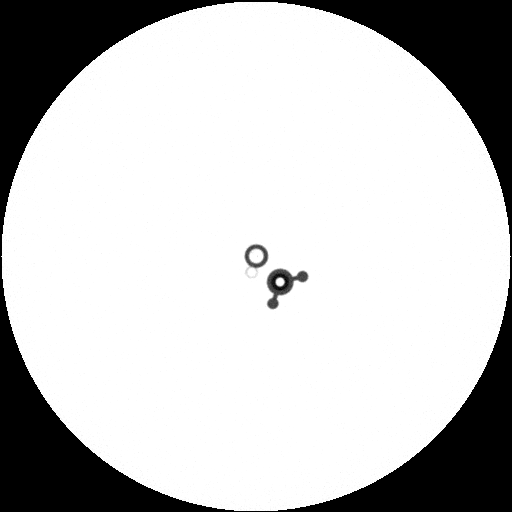

Supplement: S1 File — (ZIP) [file pone.0176383.s001.zip › Raw Image Data of a carburetor/CC-146.bmp]

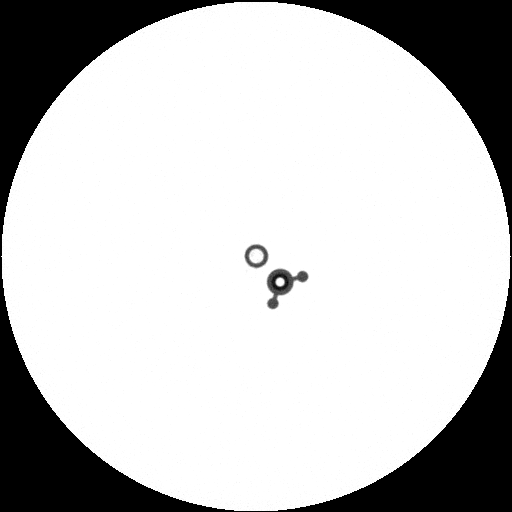

Supplement: S1 File — (ZIP) [file pone.0176383.s001.zip › Raw Image Data of a carburetor/CC-147.bmp]

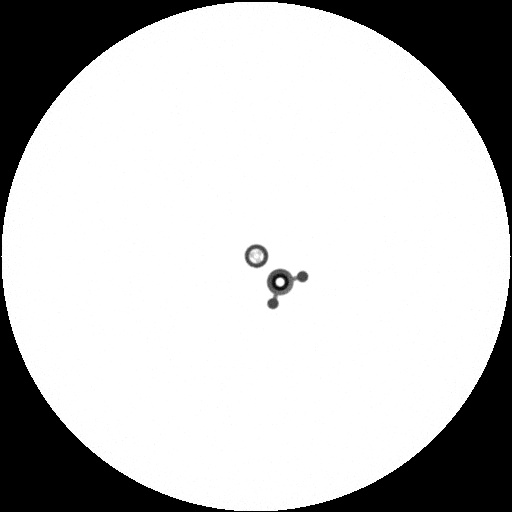

Supplement: S1 File — (ZIP) [file pone.0176383.s001.zip › Raw Image Data of a carburetor/CC-148.bmp]

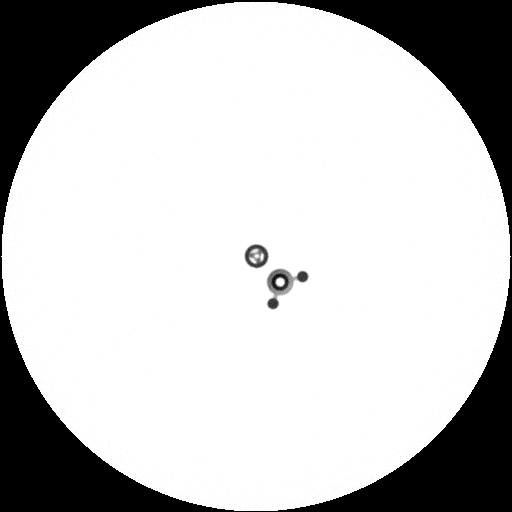

Supplement: S1 File — (ZIP) [file pone.0176383.s001.zip › Raw Image Data of a carburetor/CC-149.bmp]

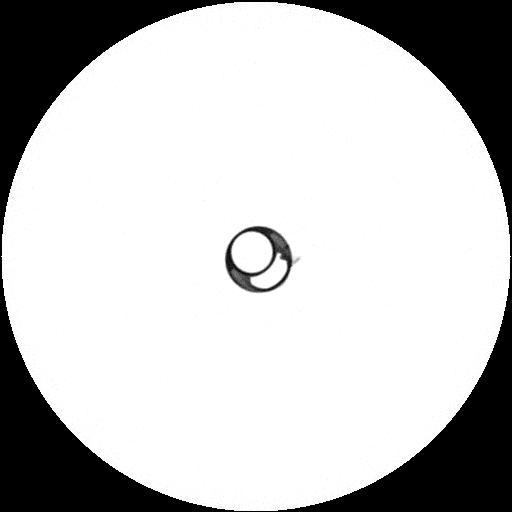

Supplement: S1 File — (ZIP) [file pone.0176383.s001.zip › Raw Image Data of a carburetor/CC-15.bmp]

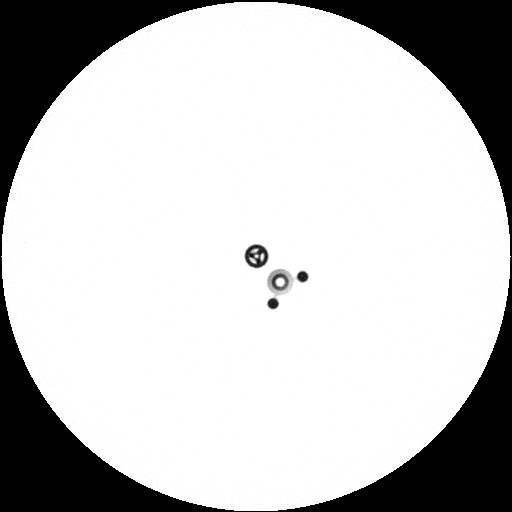

Supplement: S1 File — (ZIP) [file pone.0176383.s001.zip › Raw Image Data of a carburetor/CC-150.bmp]

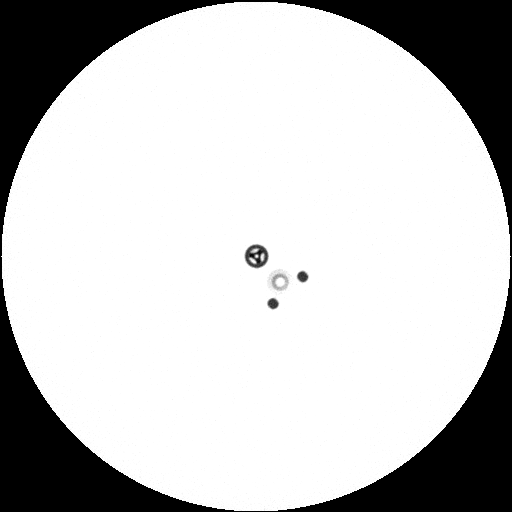

Supplement: S1 File — (ZIP) [file pone.0176383.s001.zip › Raw Image Data of a carburetor/CC-151.bmp]

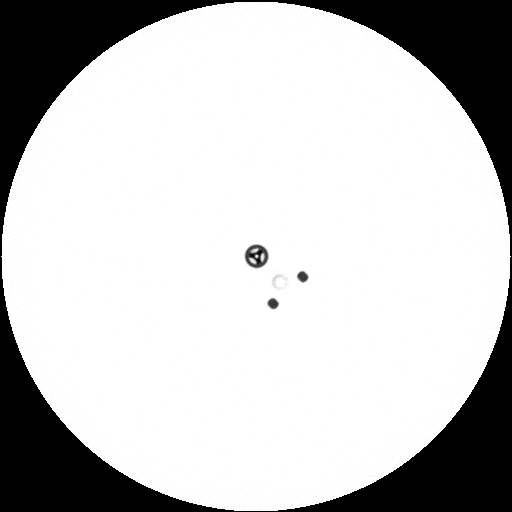

Supplement: S1 File — (ZIP) [file pone.0176383.s001.zip › Raw Image Data of a carburetor/CC-152.bmp]

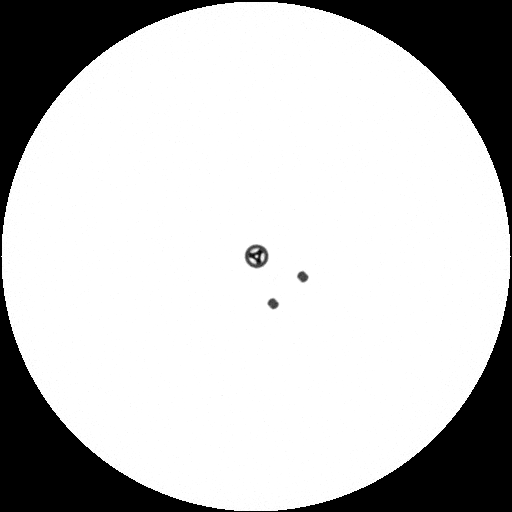

Supplement: S1 File — (ZIP) [file pone.0176383.s001.zip › Raw Image Data of a carburetor/CC-153.bmp]

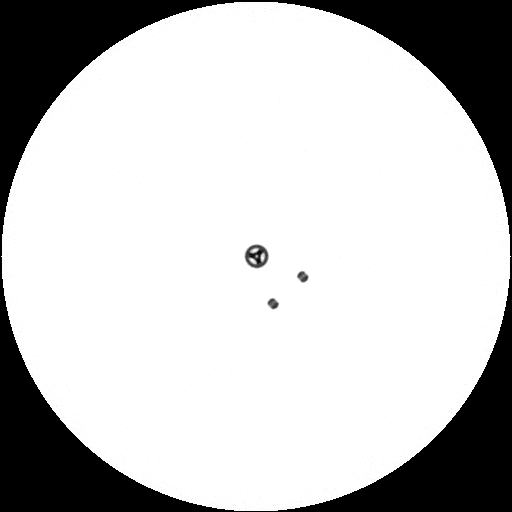

Supplement: S1 File — (ZIP) [file pone.0176383.s001.zip › Raw Image Data of a carburetor/CC-154.bmp]

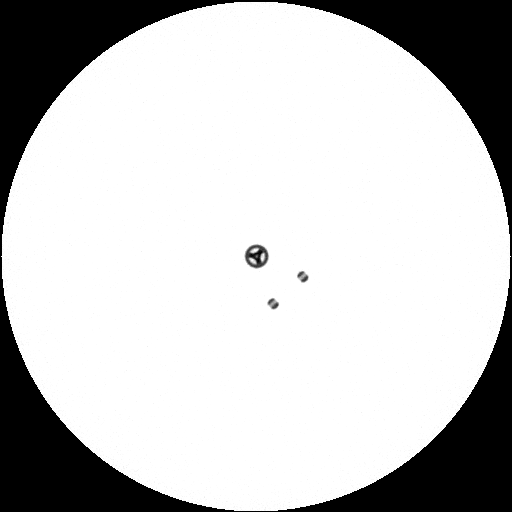

Supplement: S1 File — (ZIP) [file pone.0176383.s001.zip › Raw Image Data of a carburetor/CC-155.bmp]

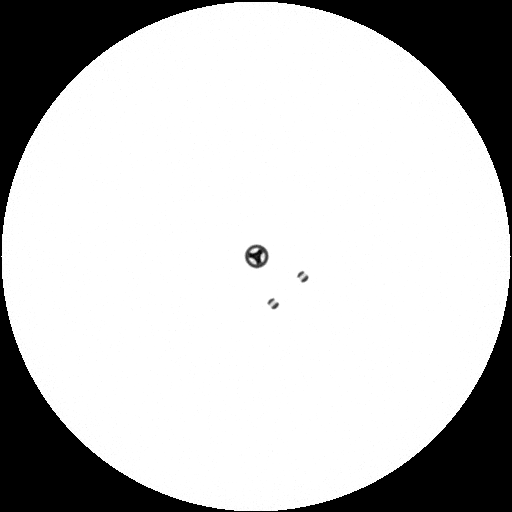

Supplement: S1 File — (ZIP) [file pone.0176383.s001.zip › Raw Image Data of a carburetor/CC-156.bmp]

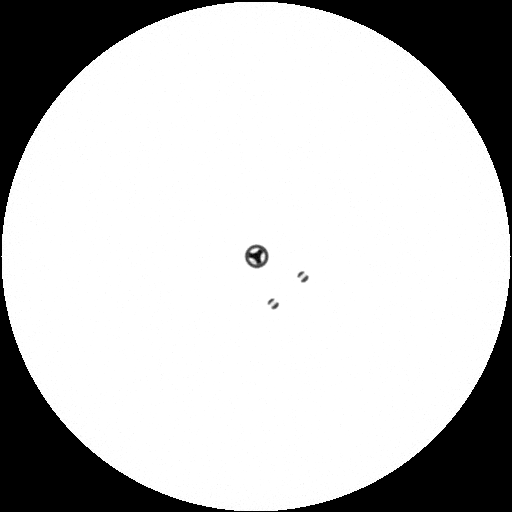

Supplement: S1 File — (ZIP) [file pone.0176383.s001.zip › Raw Image Data of a carburetor/CC-157.bmp]

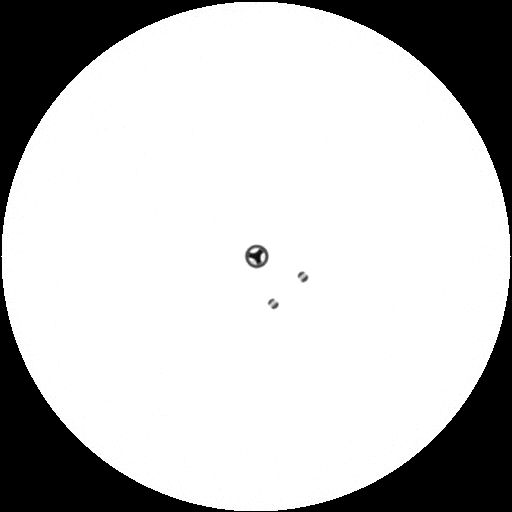

Supplement: S1 File — (ZIP) [file pone.0176383.s001.zip › Raw Image Data of a carburetor/CC-158.bmp]

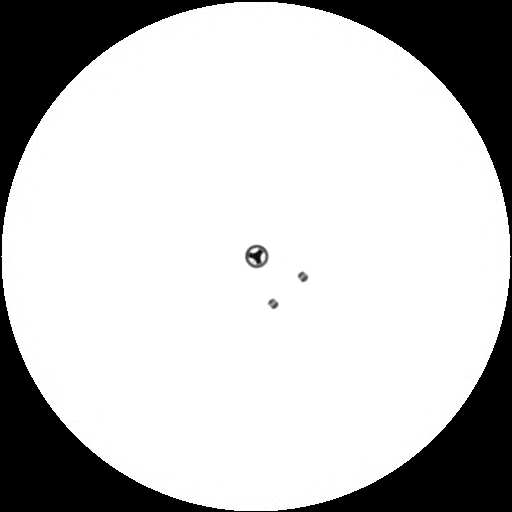

Supplement: S1 File — (ZIP) [file pone.0176383.s001.zip › Raw Image Data of a carburetor/CC-159.bmp]

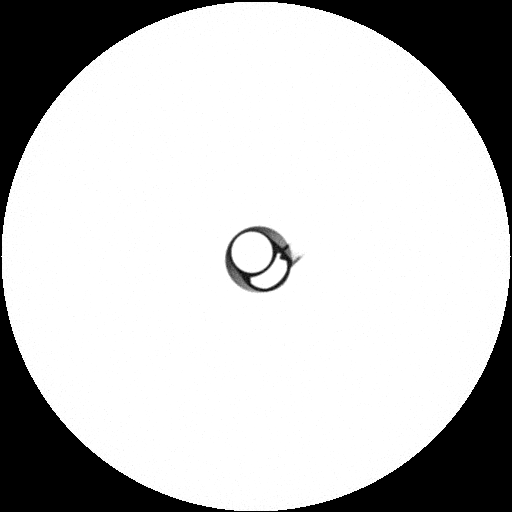

Supplement: S1 File — (ZIP) [file pone.0176383.s001.zip › Raw Image Data of a carburetor/CC-16.bmp]

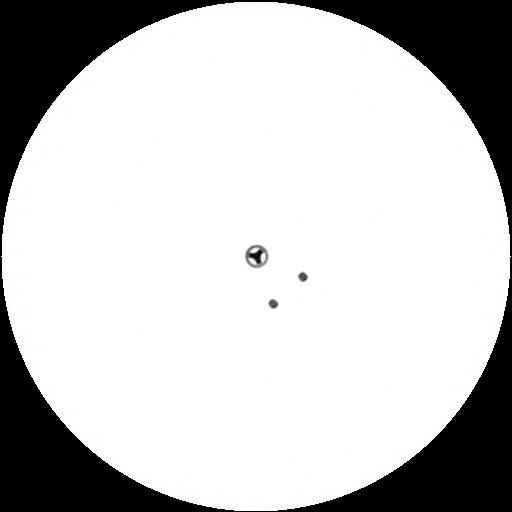

Supplement: S1 File — (ZIP) [file pone.0176383.s001.zip › Raw Image Data of a carburetor/CC-160.bmp]

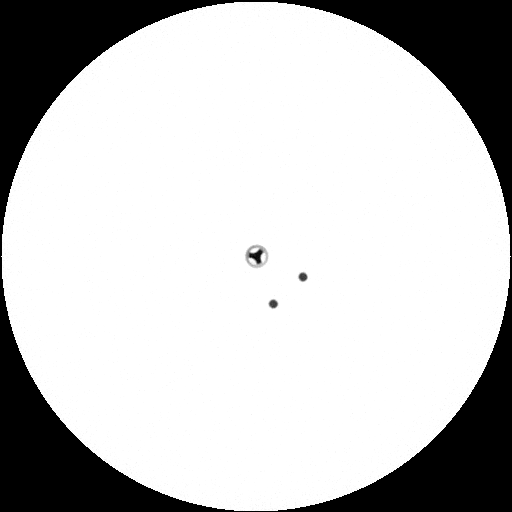

Supplement: S1 File — (ZIP) [file pone.0176383.s001.zip › Raw Image Data of a carburetor/CC-161.bmp]

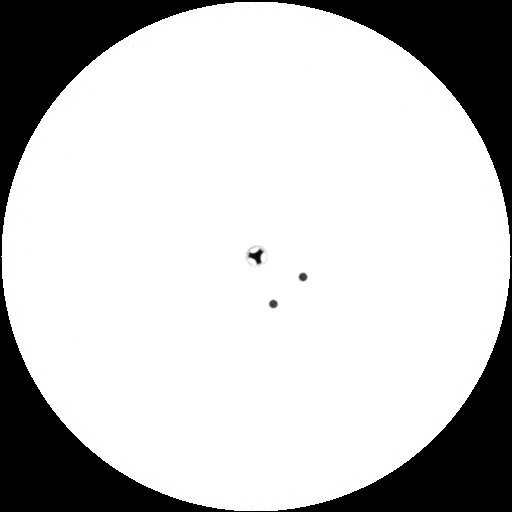

Supplement: S1 File — (ZIP) [file pone.0176383.s001.zip › Raw Image Data of a carburetor/CC-162.bmp]

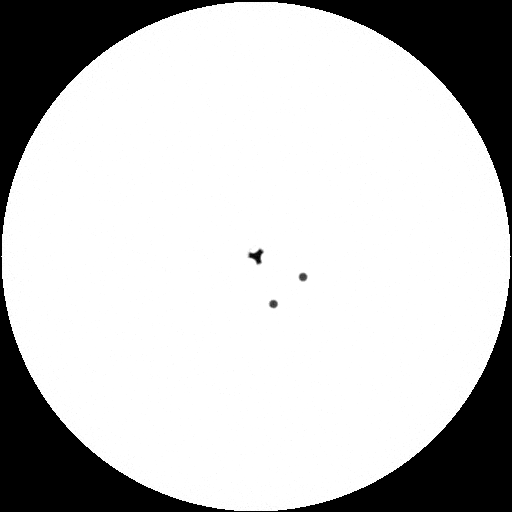

Supplement: S1 File — (ZIP) [file pone.0176383.s001.zip › Raw Image Data of a carburetor/CC-163.bmp]

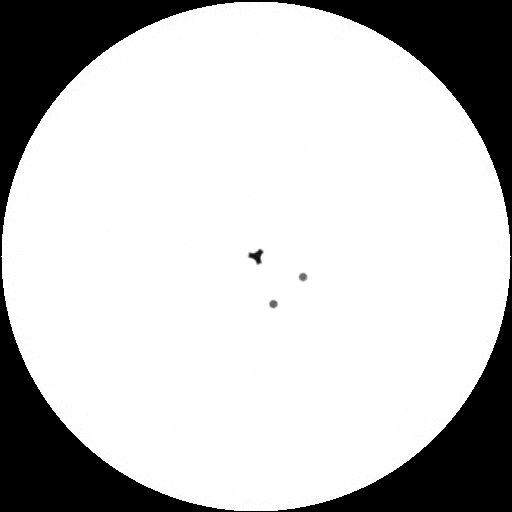

Supplement: S1 File — (ZIP) [file pone.0176383.s001.zip › Raw Image Data of a carburetor/CC-164.bmp]

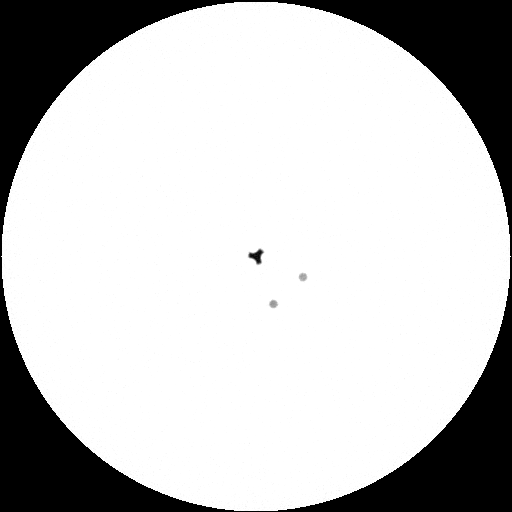

Supplement: S1 File — (ZIP) [file pone.0176383.s001.zip › Raw Image Data of a carburetor/CC-165.bmp]

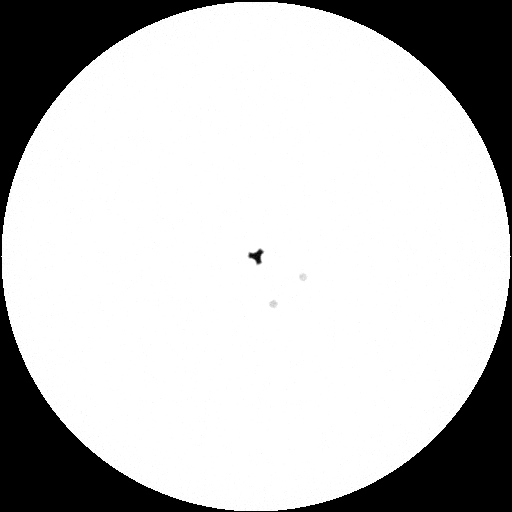

Supplement: S1 File — (ZIP) [file pone.0176383.s001.zip › Raw Image Data of a carburetor/CC-166.bmp]

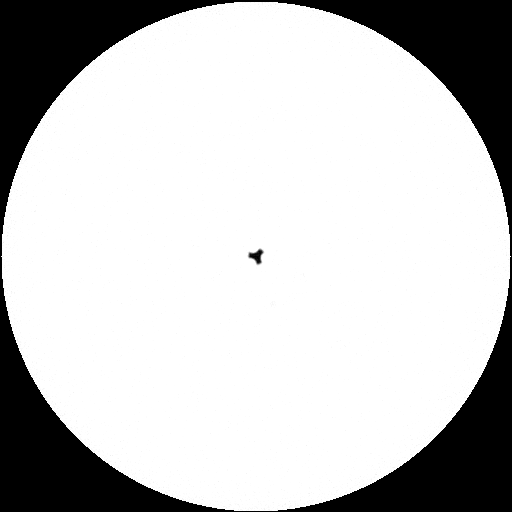

Supplement: S1 File — (ZIP) [file pone.0176383.s001.zip › Raw Image Data of a carburetor/CC-167.bmp]

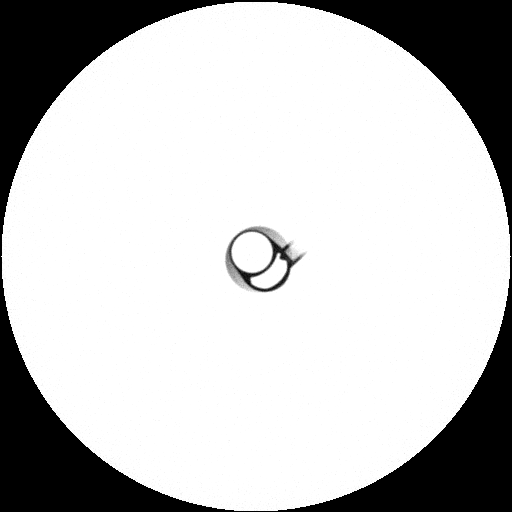

Supplement: S1 File — (ZIP) [file pone.0176383.s001.zip › Raw Image Data of a carburetor/CC-17.bmp]

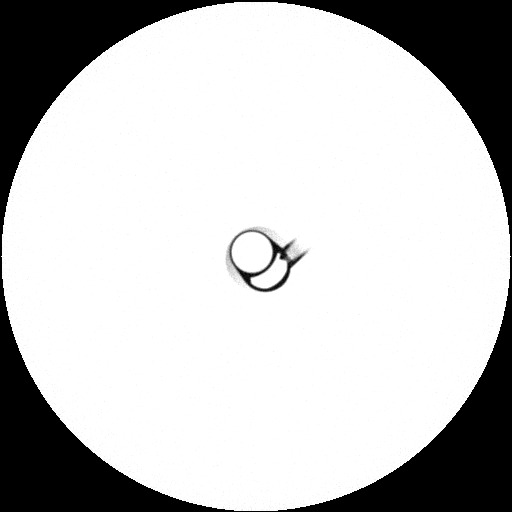

Supplement: S1 File — (ZIP) [file pone.0176383.s001.zip › Raw Image Data of a carburetor/CC-18.bmp]

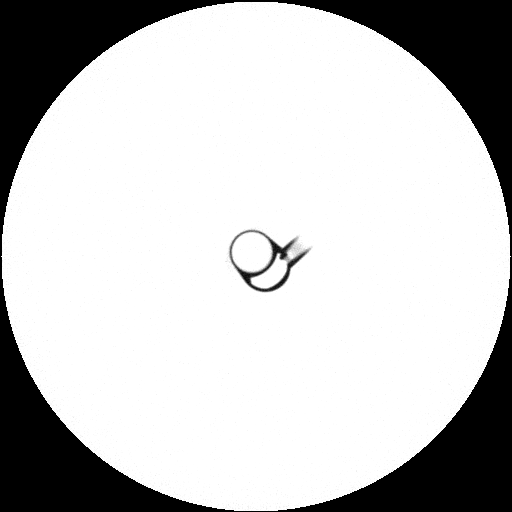

Supplement: S1 File — (ZIP) [file pone.0176383.s001.zip › Raw Image Data of a carburetor/CC-19.bmp]

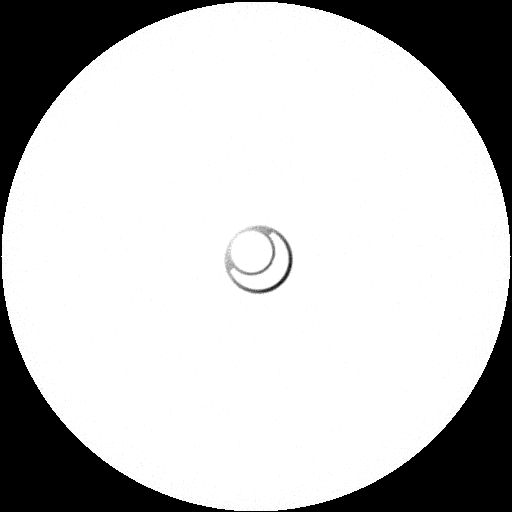

Supplement: S1 File — (ZIP) [file pone.0176383.s001.zip › Raw Image Data of a carburetor/CC-2.bmp]

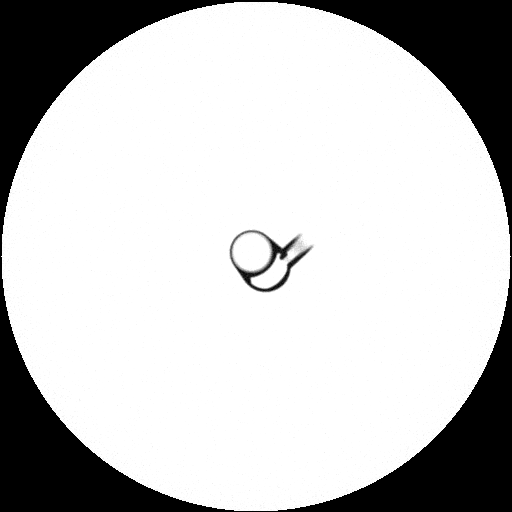

Supplement: S1 File — (ZIP) [file pone.0176383.s001.zip › Raw Image Data of a carburetor/CC-20.bmp]

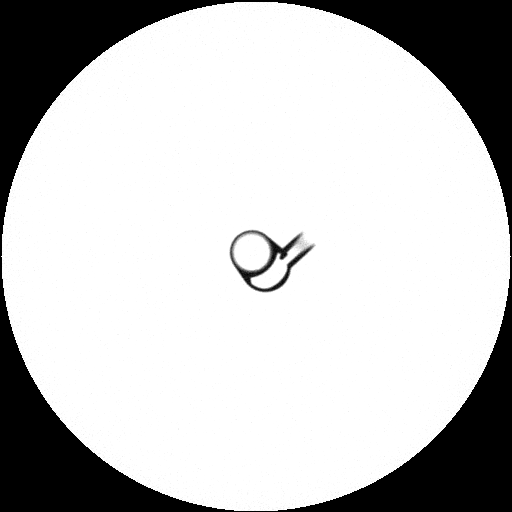

Supplement: S1 File — (ZIP) [file pone.0176383.s001.zip › Raw Image Data of a carburetor/CC-21.bmp]

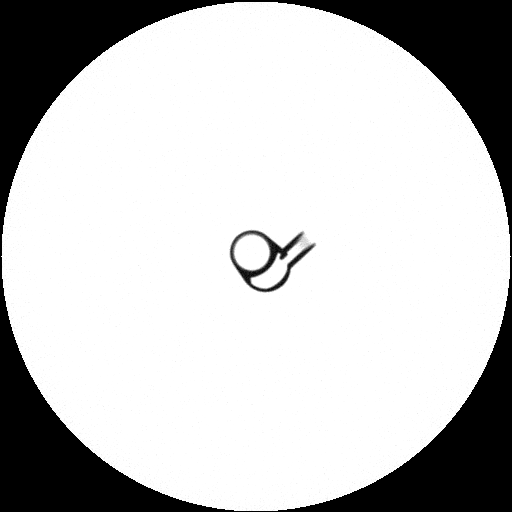

Supplement: S1 File — (ZIP) [file pone.0176383.s001.zip › Raw Image Data of a carburetor/CC-22.bmp]

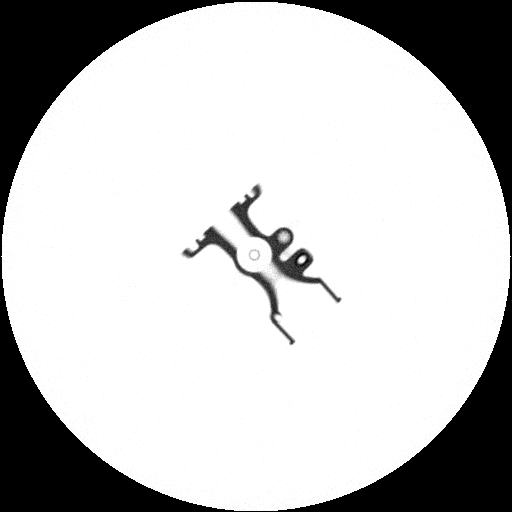

Supplement: S1 File — (ZIP) [file pone.0176383.s001.zip › Raw Image Data of a carburetor/CC-99.bmp]

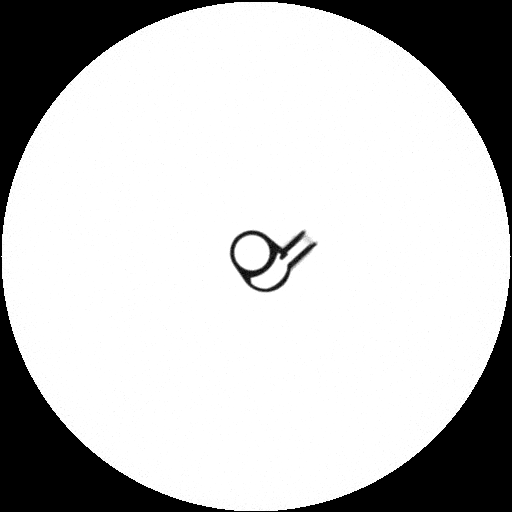

Supplement: S1 File — (ZIP) [file pone.0176383.s001.zip › Raw Image Data of a carburetor/CC-24.bmp]

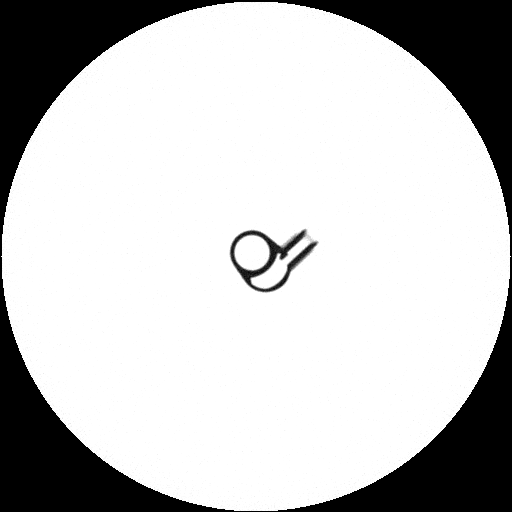

Supplement: S1 File — (ZIP) [file pone.0176383.s001.zip › Raw Image Data of a carburetor/CC-25.bmp]

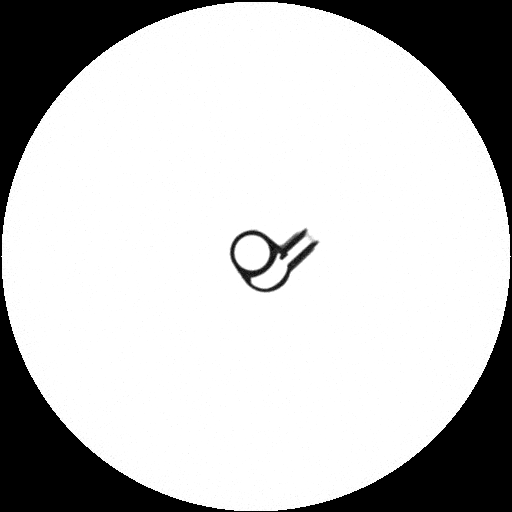

Supplement: S1 File — (ZIP) [file pone.0176383.s001.zip › Raw Image Data of a carburetor/CC-26.bmp]

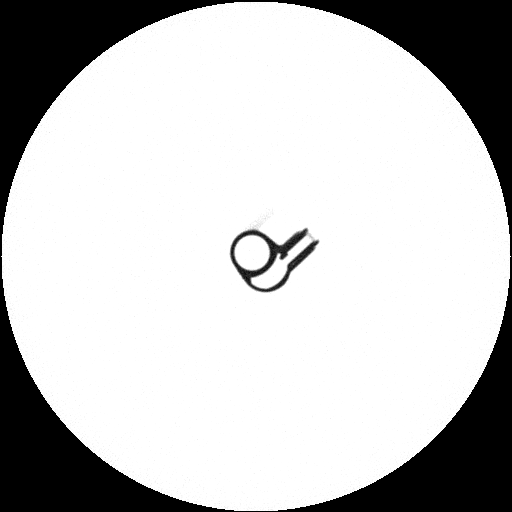

Supplement: S1 File — (ZIP) [file pone.0176383.s001.zip › Raw Image Data of a carburetor/CC-27.bmp]

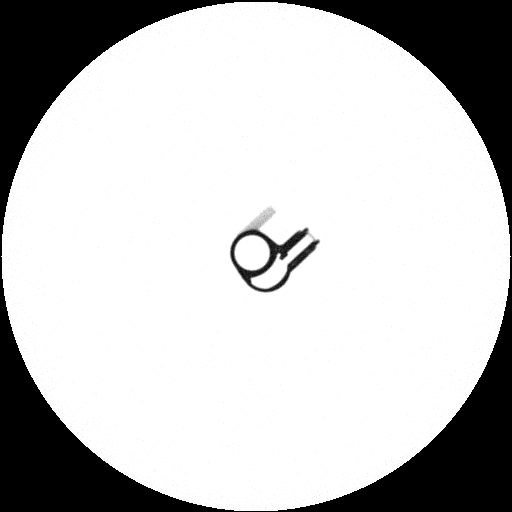

Supplement: S1 File — (ZIP) [file pone.0176383.s001.zip › Raw Image Data of a carburetor/CC-28.bmp]

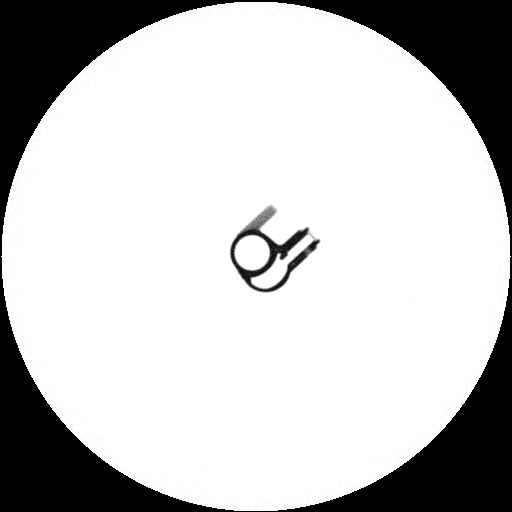

Supplement: S1 File — (ZIP) [file pone.0176383.s001.zip › Raw Image Data of a carburetor/CC-29.bmp]

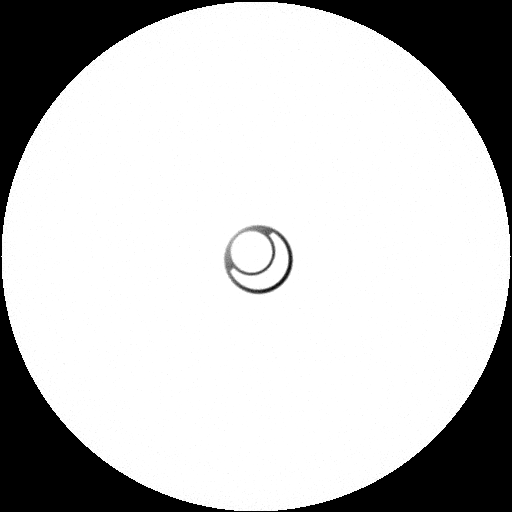

Supplement: S1 File — (ZIP) [file pone.0176383.s001.zip › Raw Image Data of a carburetor/CC-3.bmp]

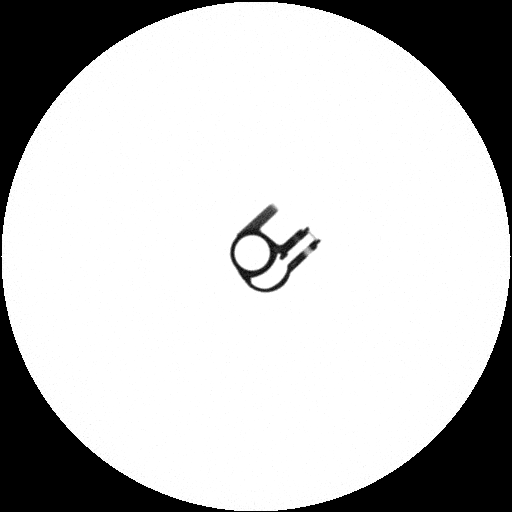

Supplement: S1 File — (ZIP) [file pone.0176383.s001.zip › Raw Image Data of a carburetor/CC-30.bmp]

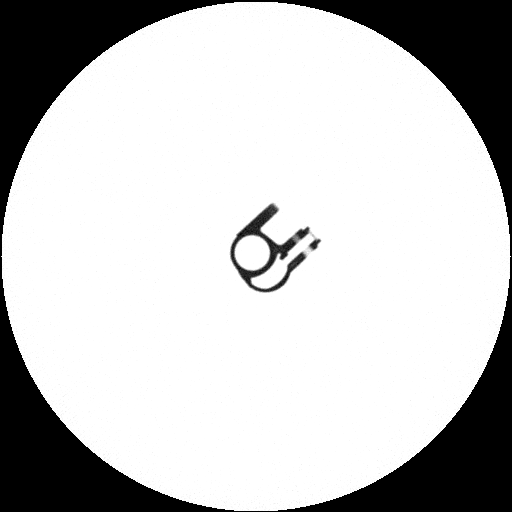

Supplement: S1 File — (ZIP) [file pone.0176383.s001.zip › Raw Image Data of a carburetor/CC-31.bmp]

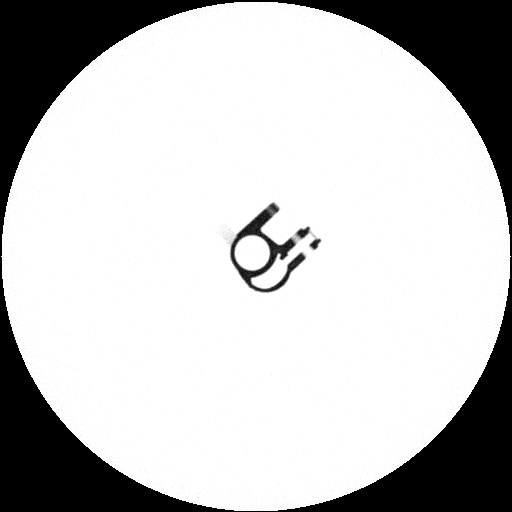

Supplement: S1 File — (ZIP) [file pone.0176383.s001.zip › Raw Image Data of a carburetor/CC-32.bmp]

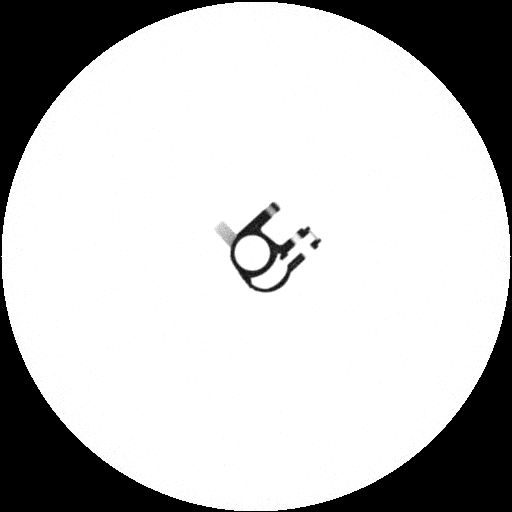

Supplement: S1 File — (ZIP) [file pone.0176383.s001.zip › Raw Image Data of a carburetor/CC-33.bmp]

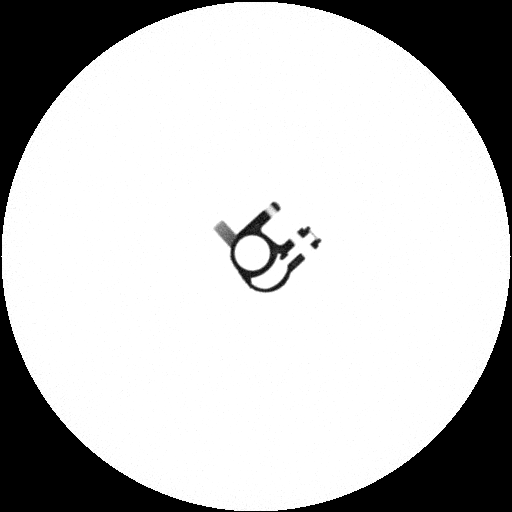

Supplement: S1 File — (ZIP) [file pone.0176383.s001.zip › Raw Image Data of a carburetor/CC-34.bmp]

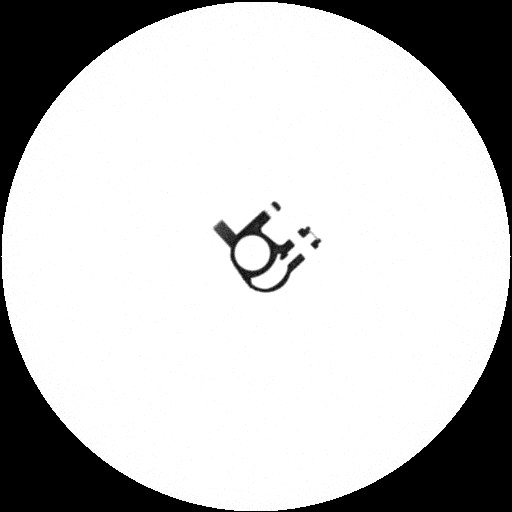

Supplement: S1 File — (ZIP) [file pone.0176383.s001.zip › Raw Image Data of a carburetor/CC-35.bmp]

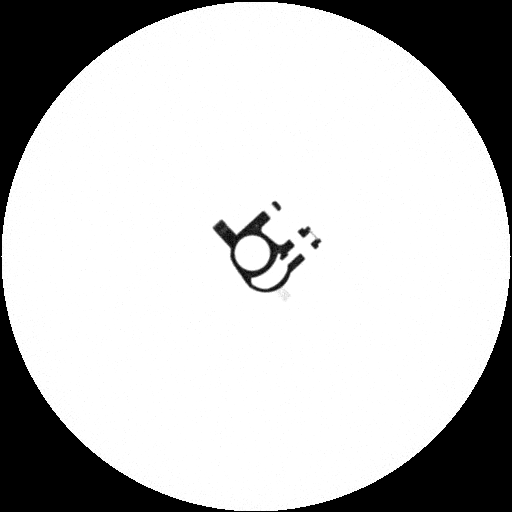

Supplement: S1 File — (ZIP) [file pone.0176383.s001.zip › Raw Image Data of a carburetor/CC-36.bmp]

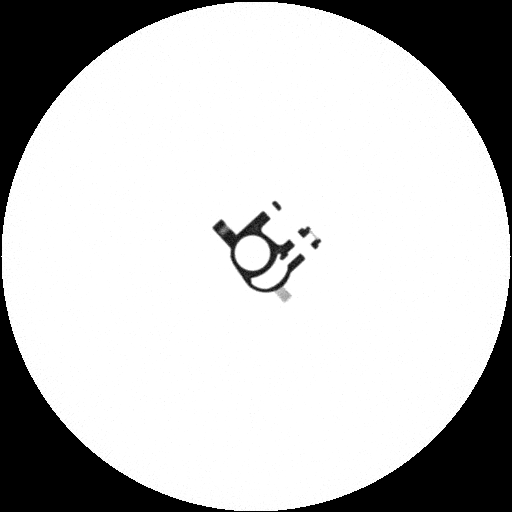

Supplement: S1 File — (ZIP) [file pone.0176383.s001.zip › Raw Image Data of a carburetor/CC-37.bmp]

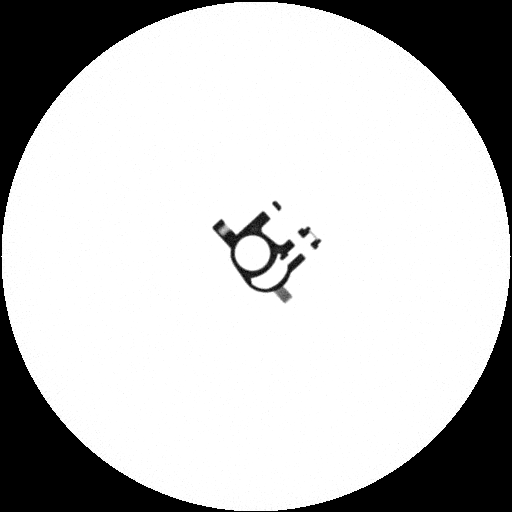

Supplement: S1 File — (ZIP) [file pone.0176383.s001.zip › Raw Image Data of a carburetor/CC-38.bmp]

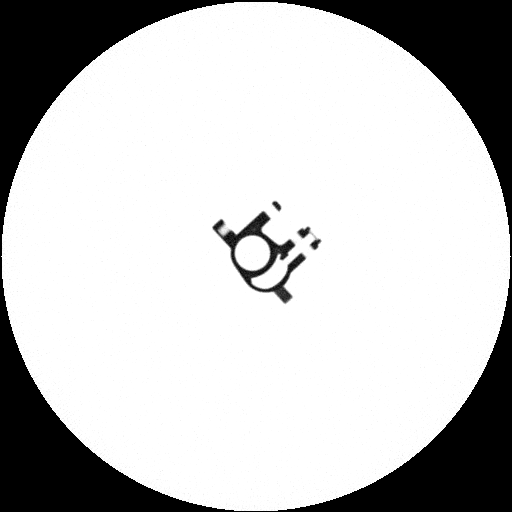

Supplement: S1 File — (ZIP) [file pone.0176383.s001.zip › Raw Image Data of a carburetor/CC-39.bmp]

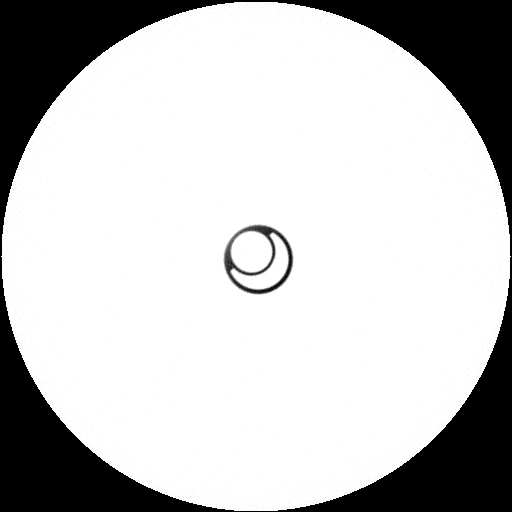

Supplement: S1 File — (ZIP) [file pone.0176383.s001.zip › Raw Image Data of a carburetor/CC-4.bmp]

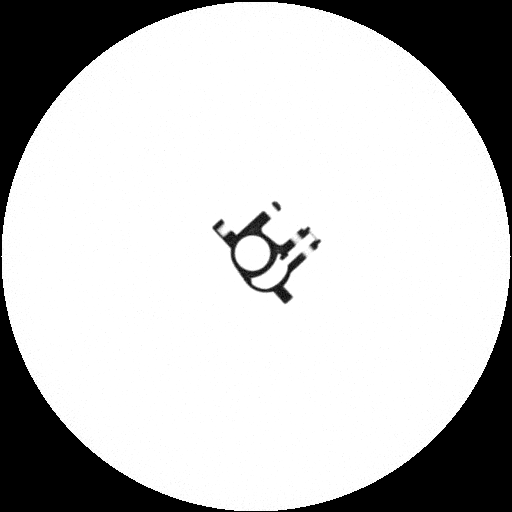

Supplement: S1 File — (ZIP) [file pone.0176383.s001.zip › Raw Image Data of a carburetor/CC-40.bmp]

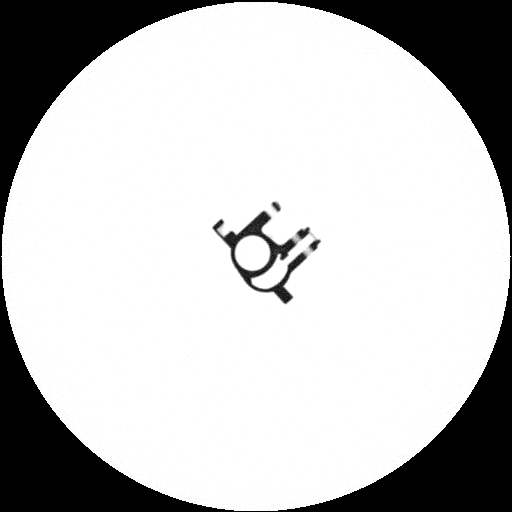

Supplement: S1 File — (ZIP) [file pone.0176383.s001.zip › Raw Image Data of a carburetor/CC-41.bmp]

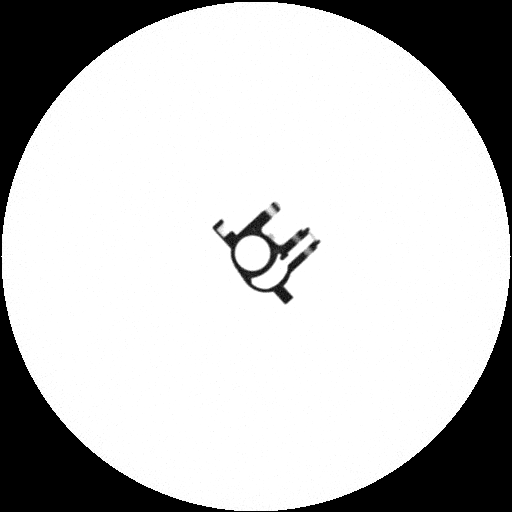

Supplement: S1 File — (ZIP) [file pone.0176383.s001.zip › Raw Image Data of a carburetor/CC-42.bmp]

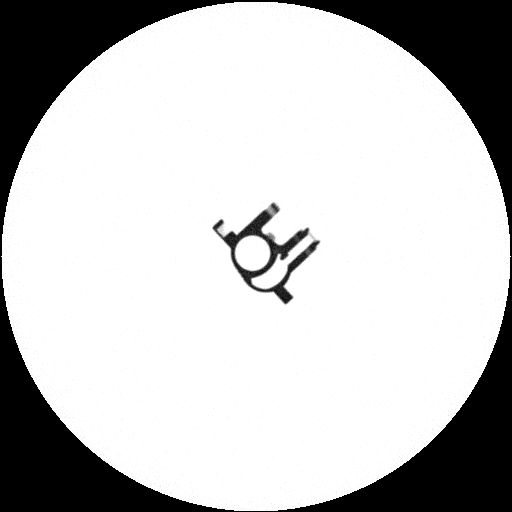

Supplement: S1 File — (ZIP) [file pone.0176383.s001.zip › Raw Image Data of a carburetor/CC-43.bmp]

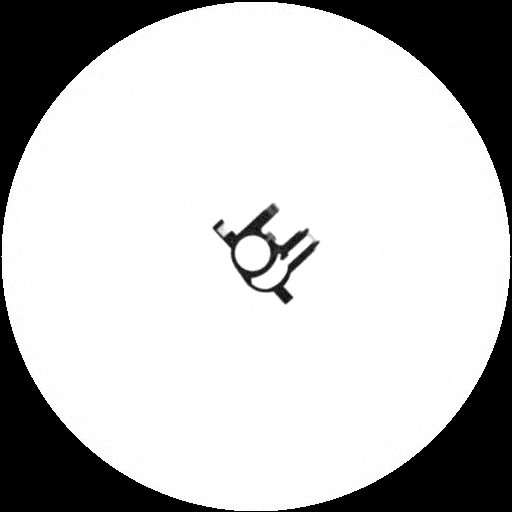

Supplement: S1 File — (ZIP) [file pone.0176383.s001.zip › Raw Image Data of a carburetor/CC-44.bmp]

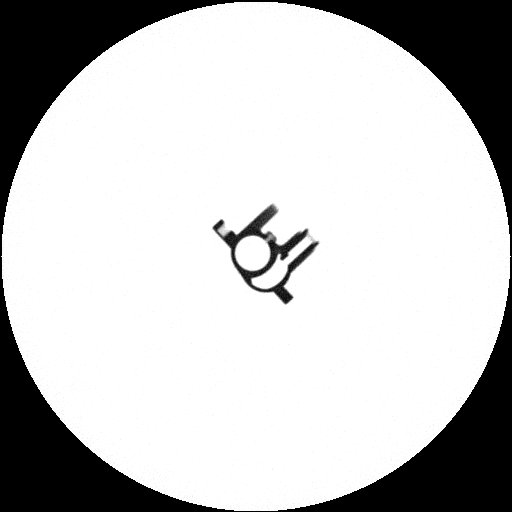

Supplement: S1 File — (ZIP) [file pone.0176383.s001.zip › Raw Image Data of a carburetor/CC-45.bmp]

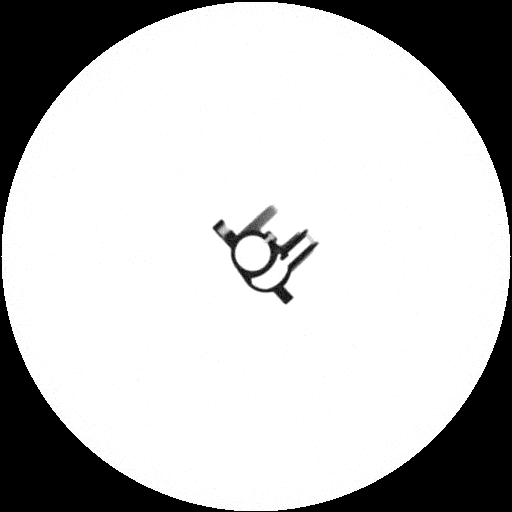

Supplement: S1 File — (ZIP) [file pone.0176383.s001.zip › Raw Image Data of a carburetor/CC-46.bmp]

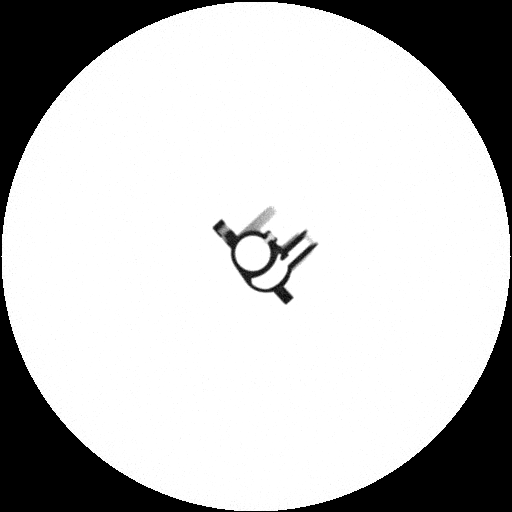

Supplement: S1 File — (ZIP) [file pone.0176383.s001.zip › Raw Image Data of a carburetor/CC-47.bmp]

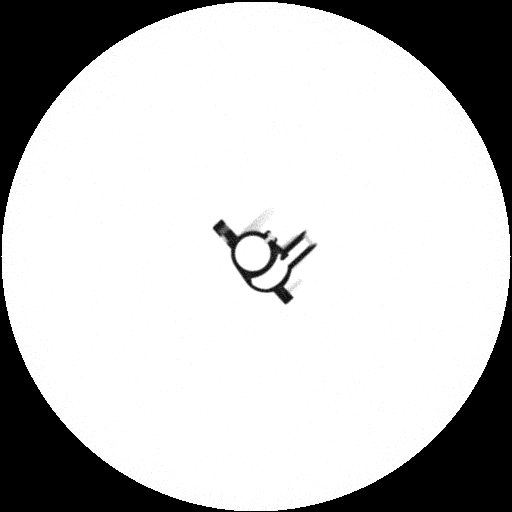

Supplement: S1 File — (ZIP) [file pone.0176383.s001.zip › Raw Image Data of a carburetor/CC-48.bmp]

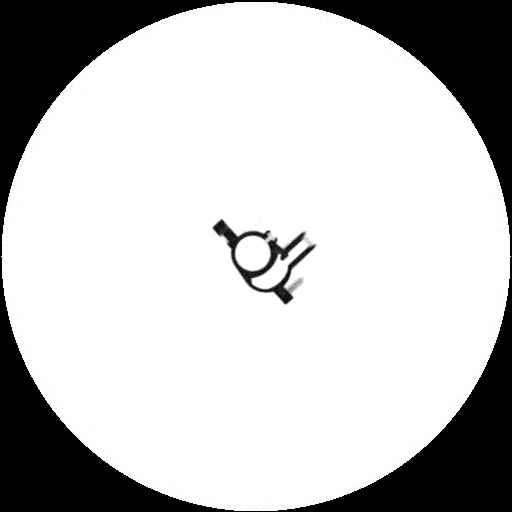

Supplement: S1 File — (ZIP) [file pone.0176383.s001.zip › Raw Image Data of a carburetor/CC-49.bmp]

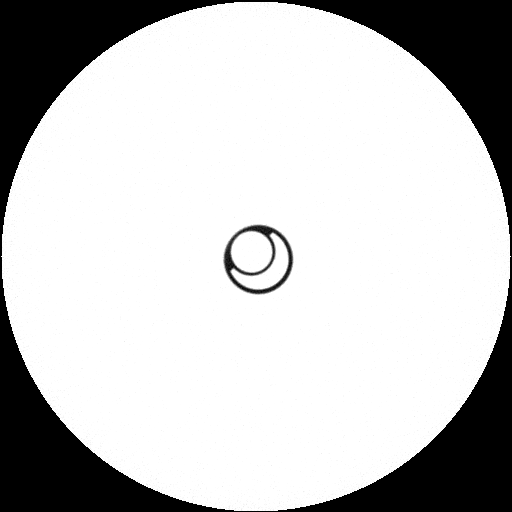

Supplement: S1 File — (ZIP) [file pone.0176383.s001.zip › Raw Image Data of a carburetor/CC-5.bmp]

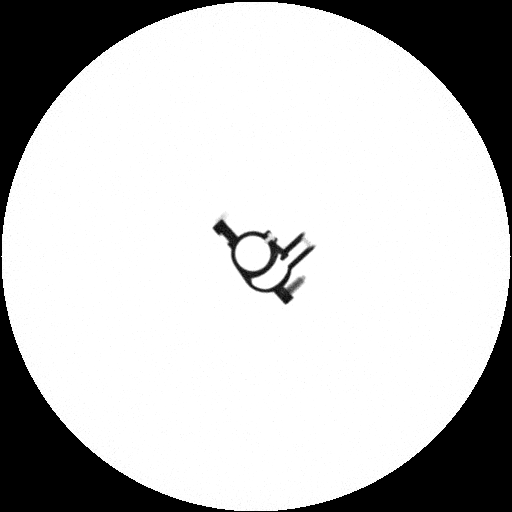

Supplement: S1 File — (ZIP) [file pone.0176383.s001.zip › Raw Image Data of a carburetor/CC-50.bmp]

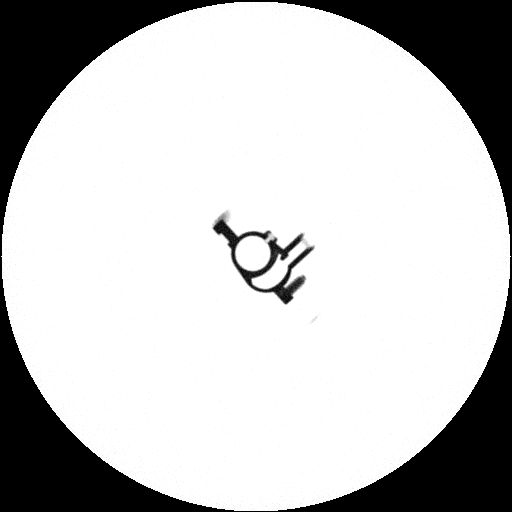

Supplement: S1 File — (ZIP) [file pone.0176383.s001.zip › Raw Image Data of a carburetor/CC-51.bmp]

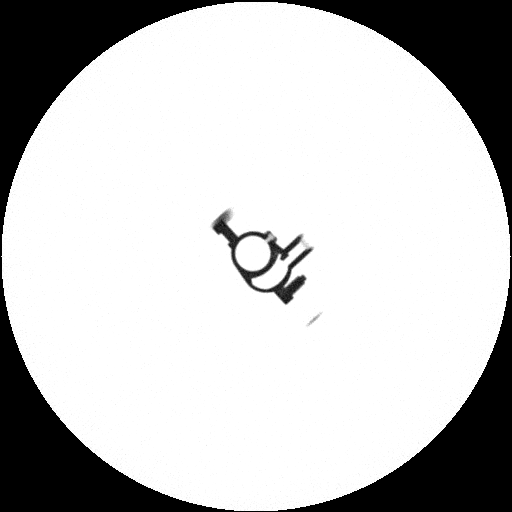

Supplement: S1 File — (ZIP) [file pone.0176383.s001.zip › Raw Image Data of a carburetor/CC-52.bmp]

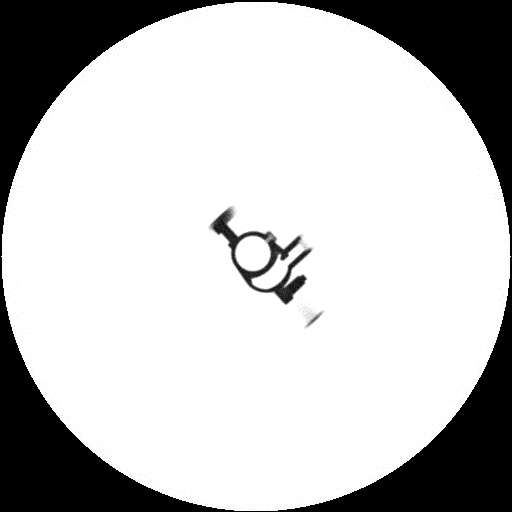

Supplement: S1 File — (ZIP) [file pone.0176383.s001.zip › Raw Image Data of a carburetor/CC-53.bmp]

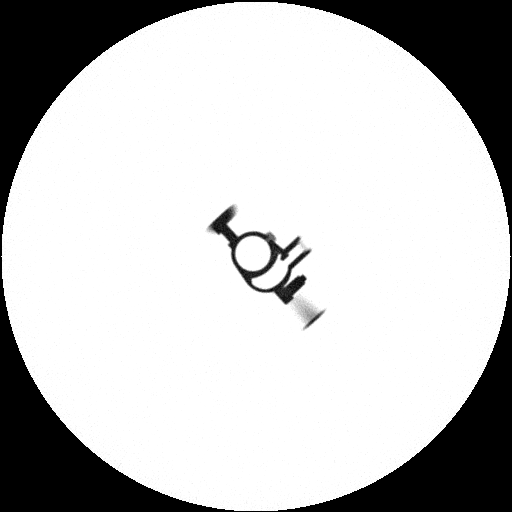

Supplement: S1 File — (ZIP) [file pone.0176383.s001.zip › Raw Image Data of a carburetor/CC-54.bmp]

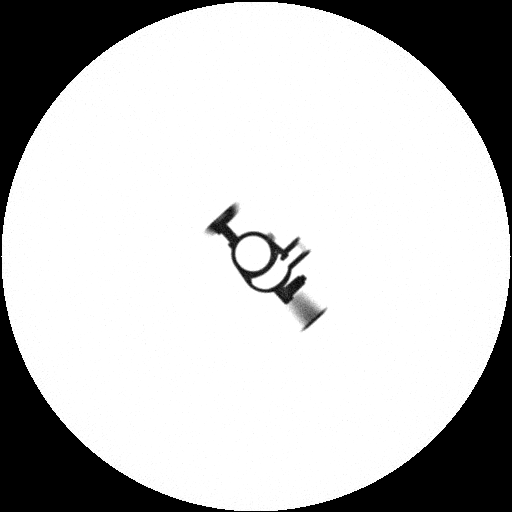

Supplement: S1 File — (ZIP) [file pone.0176383.s001.zip › Raw Image Data of a carburetor/CC-55.bmp]

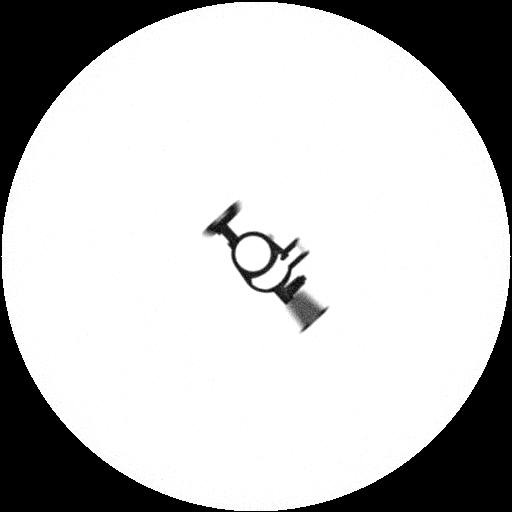

Supplement: S1 File — (ZIP) [file pone.0176383.s001.zip › Raw Image Data of a carburetor/CC-56.bmp]

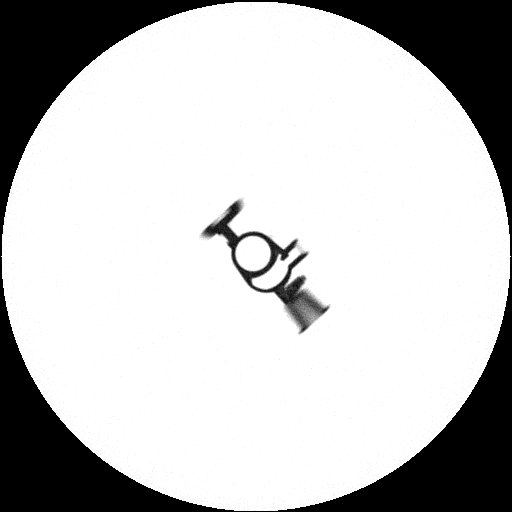

Supplement: S1 File — (ZIP) [file pone.0176383.s001.zip › Raw Image Data of a carburetor/CC-57.bmp]

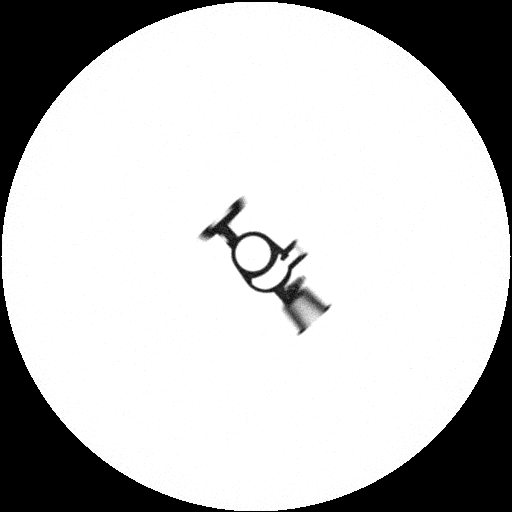

Supplement: S1 File — (ZIP) [file pone.0176383.s001.zip › Raw Image Data of a carburetor/CC-58.bmp]

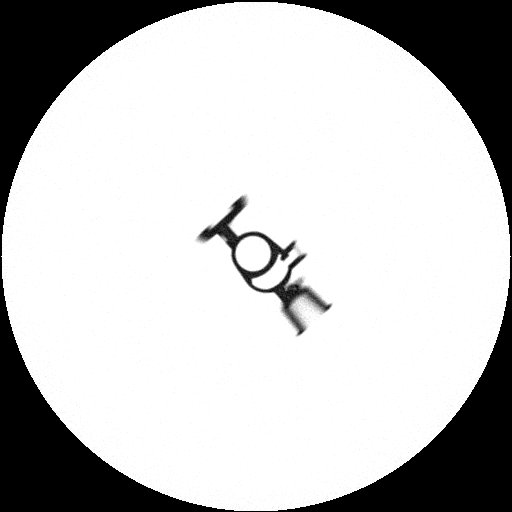

Supplement: S1 File — (ZIP) [file pone.0176383.s001.zip › Raw Image Data of a carburetor/CC-59.bmp]

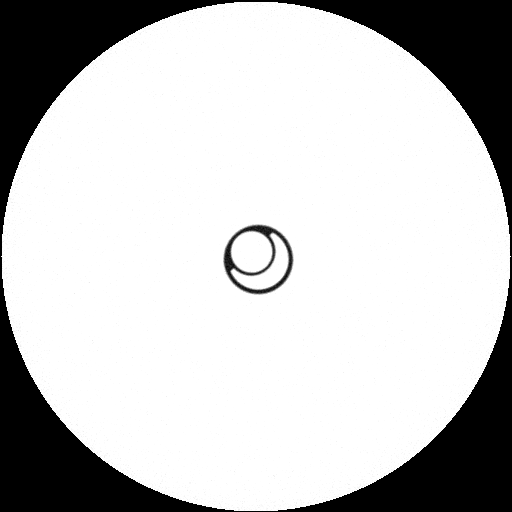

Supplement: S1 File — (ZIP) [file pone.0176383.s001.zip › Raw Image Data of a carburetor/CC-6.bmp]

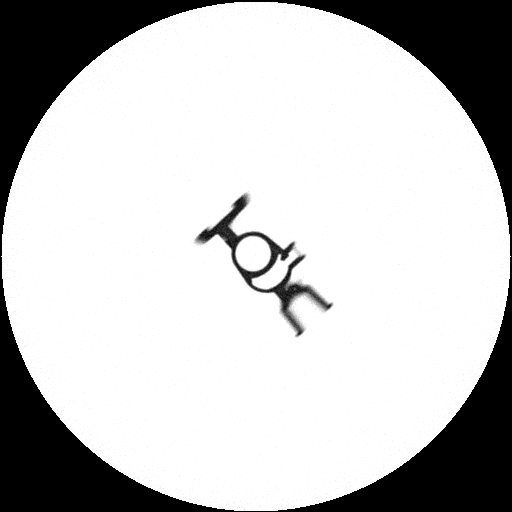

Supplement: S1 File — (ZIP) [file pone.0176383.s001.zip › Raw Image Data of a carburetor/CC-60.bmp]

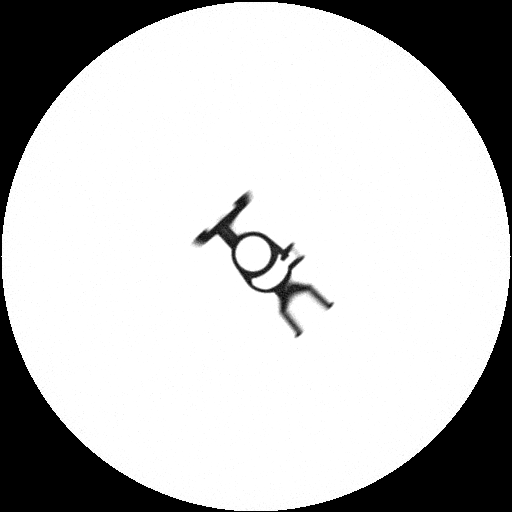

Supplement: S1 File — (ZIP) [file pone.0176383.s001.zip › Raw Image Data of a carburetor/CC-61.bmp]

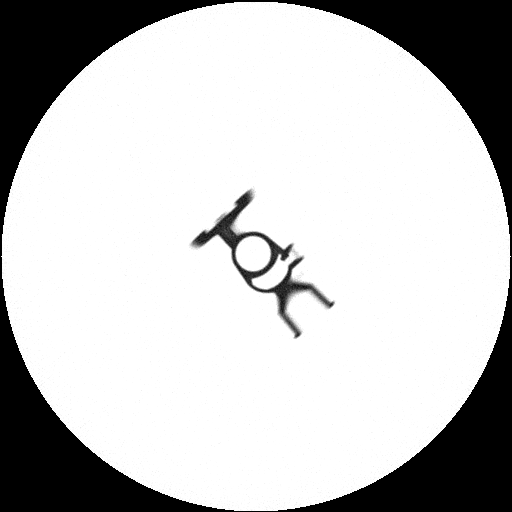

Supplement: S1 File — (ZIP) [file pone.0176383.s001.zip › Raw Image Data of a carburetor/CC-62.bmp]

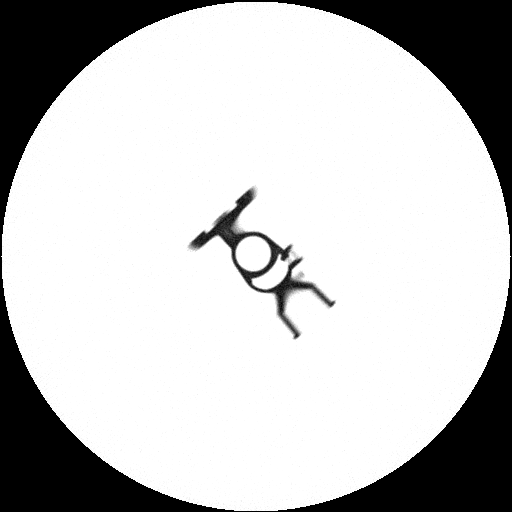

Supplement: S1 File — (ZIP) [file pone.0176383.s001.zip › Raw Image Data of a carburetor/CC-63.bmp]

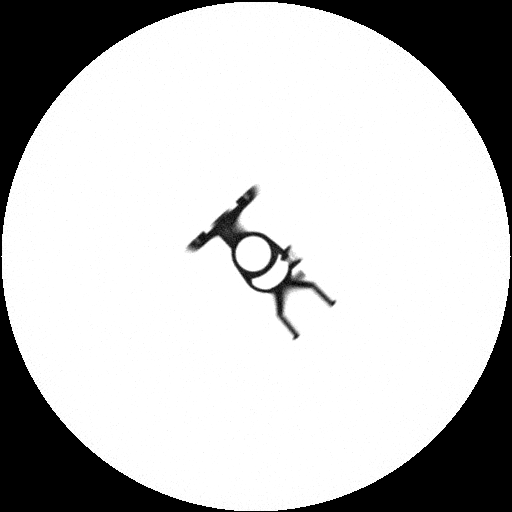

Supplement: S1 File — (ZIP) [file pone.0176383.s001.zip › Raw Image Data of a carburetor/CC-64.bmp]

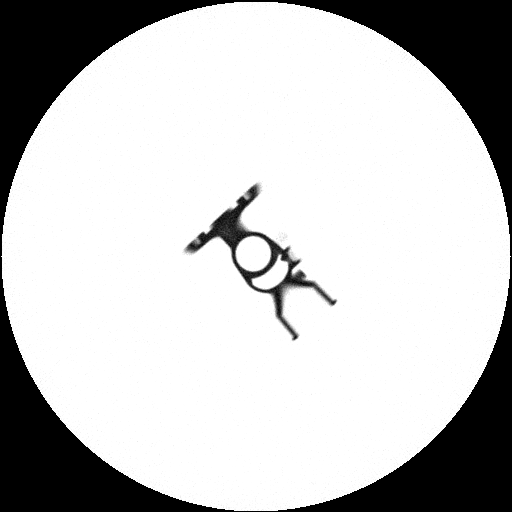

Supplement: S1 File — (ZIP) [file pone.0176383.s001.zip › Raw Image Data of a carburetor/CC-65.bmp]

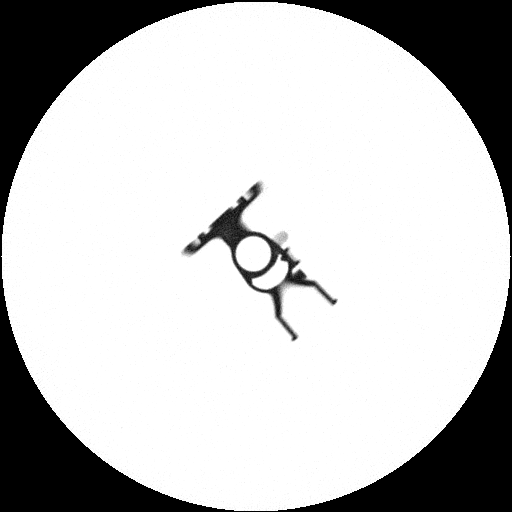

Supplement: S1 File — (ZIP) [file pone.0176383.s001.zip › Raw Image Data of a carburetor/CC-66.bmp]

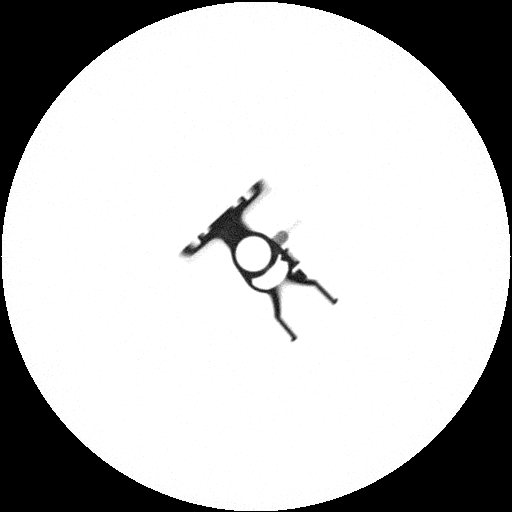

Supplement: S1 File — (ZIP) [file pone.0176383.s001.zip › Raw Image Data of a carburetor/CC-67.bmp]
