# Supplementary figures and images for: A shape-based inter-layer contours correspondence method for ICT-based reverse engineering (part 1 of 2)
Source: PLoS One. 2017 May 10;12(5):e0176383. doi: 10.1371/journal.pone.0176383 (PMC5425182; doi:10.1371/journal.pone.0176383)

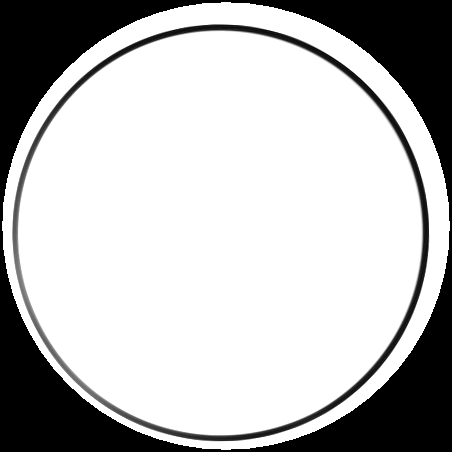

Supplement: S1 File — (ZIP) [file pone.0176383.s001.zip › Raw Image Data of a hub/200503180083.bmp]

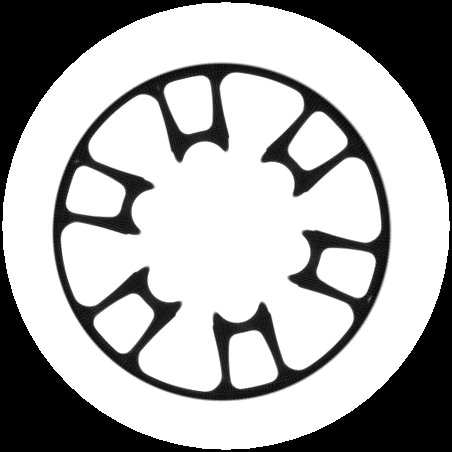

Supplement: S1 File — (ZIP) [file pone.0176383.s001.zip › Raw Image Data of a hub/200503180033.bmp]

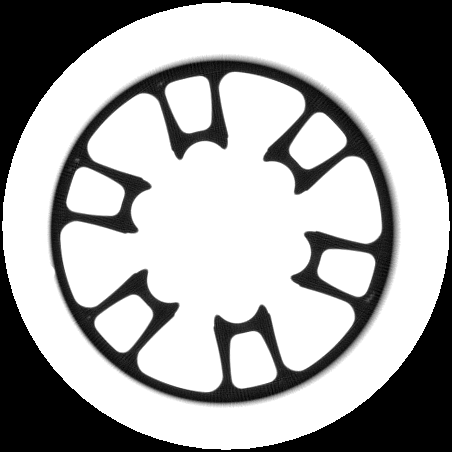

Supplement: S1 File — (ZIP) [file pone.0176383.s001.zip › Raw Image Data of a hub/200503180032.bmp]

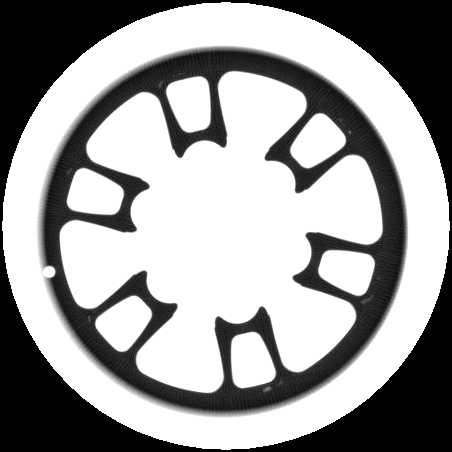

Supplement: S1 File — (ZIP) [file pone.0176383.s001.zip › Raw Image Data of a hub/200503180031.bmp]

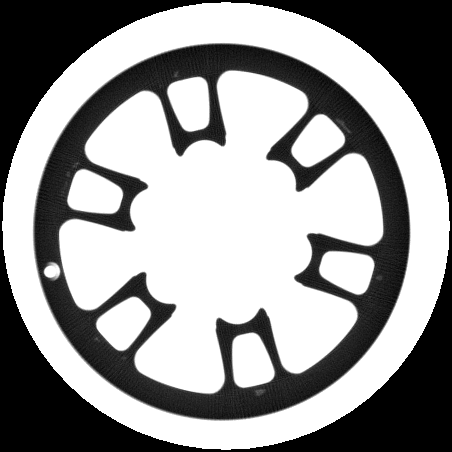

Supplement: S1 File — (ZIP) [file pone.0176383.s001.zip › Raw Image Data of a hub/200503180030.bmp]

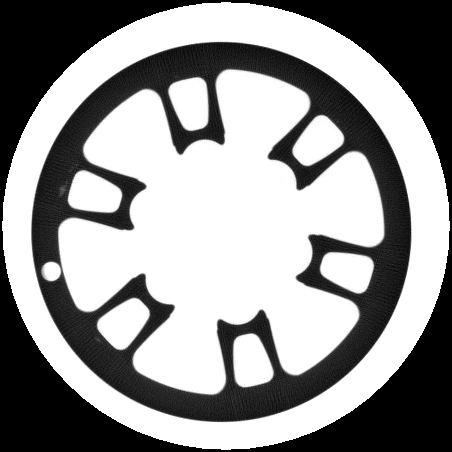

Supplement: S1 File — (ZIP) [file pone.0176383.s001.zip › Raw Image Data of a hub/200503180029.bmp]

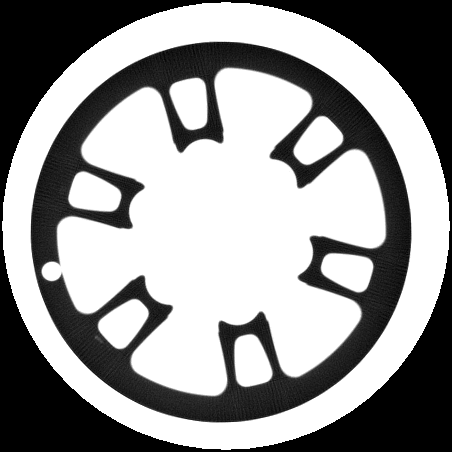

Supplement: S1 File — (ZIP) [file pone.0176383.s001.zip › Raw Image Data of a hub/200503180028.bmp]

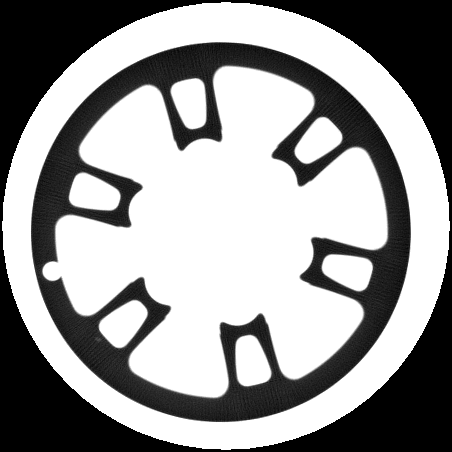

Supplement: S1 File — (ZIP) [file pone.0176383.s001.zip › Raw Image Data of a hub/200503180027.bmp]

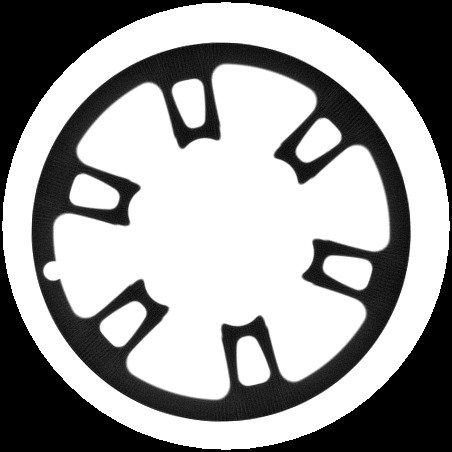

Supplement: S1 File — (ZIP) [file pone.0176383.s001.zip › Raw Image Data of a hub/200503180026.bmp]

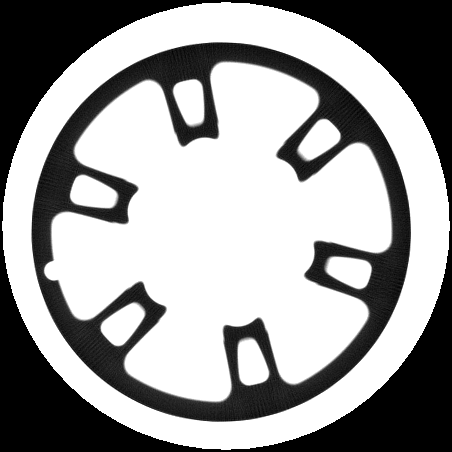

Supplement: S1 File — (ZIP) [file pone.0176383.s001.zip › Raw Image Data of a hub/200503180025.bmp]

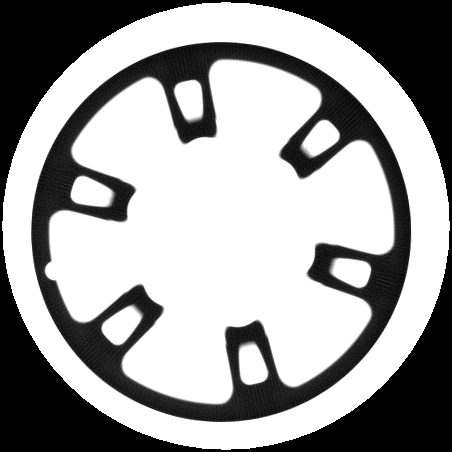

Supplement: S1 File — (ZIP) [file pone.0176383.s001.zip › Raw Image Data of a hub/200503180024.bmp]

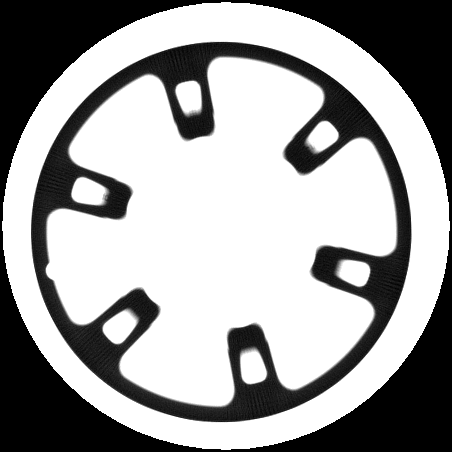

Supplement: S1 File — (ZIP) [file pone.0176383.s001.zip › Raw Image Data of a hub/200503180023.bmp]

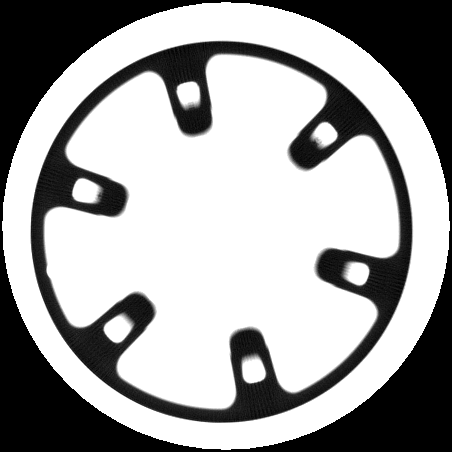

Supplement: S1 File — (ZIP) [file pone.0176383.s001.zip › Raw Image Data of a hub/200503180022.bmp]

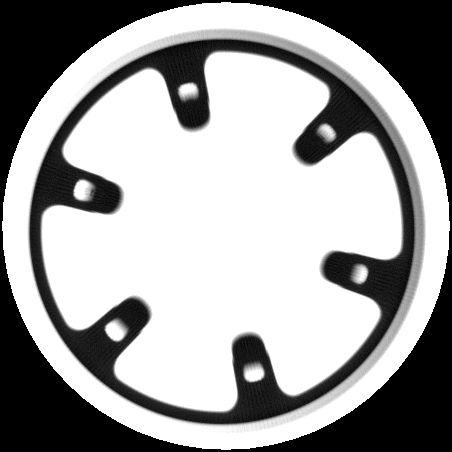

Supplement: S1 File — (ZIP) [file pone.0176383.s001.zip › Raw Image Data of a hub/200503180021.bmp]

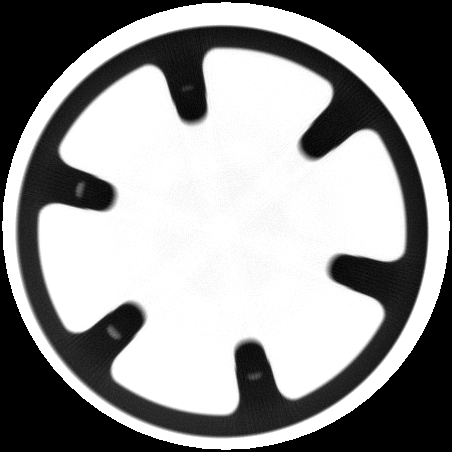

Supplement: S1 File — (ZIP) [file pone.0176383.s001.zip › Raw Image Data of a hub/200503180020.bmp]

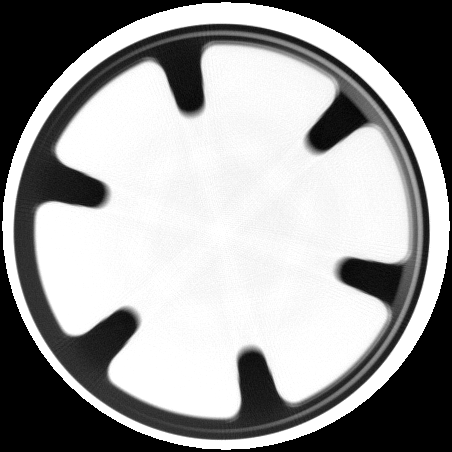

Supplement: S1 File — (ZIP) [file pone.0176383.s001.zip › Raw Image Data of a hub/200503180019.bmp]

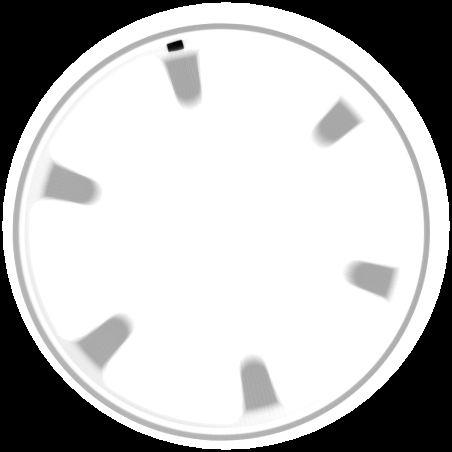

Supplement: S1 File — (ZIP) [file pone.0176383.s001.zip › Raw Image Data of a hub/200503180018.bmp]

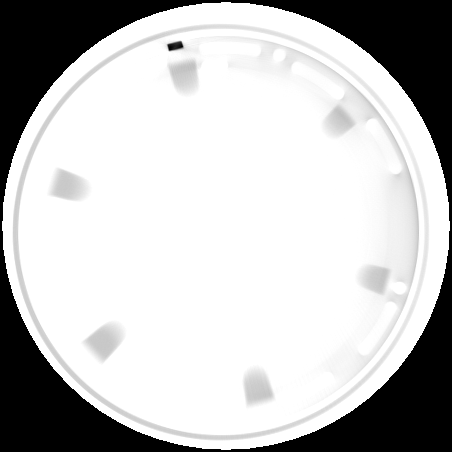

Supplement: S1 File — (ZIP) [file pone.0176383.s001.zip › Raw Image Data of a hub/200503180017.bmp]

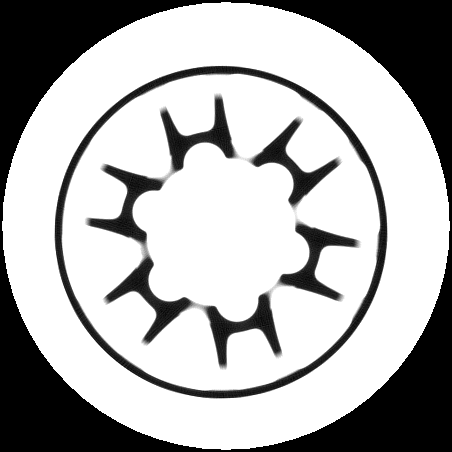

Supplement: S1 File — (ZIP) [file pone.0176383.s001.zip › Raw Image Data of a hub/200503180035.bmp]

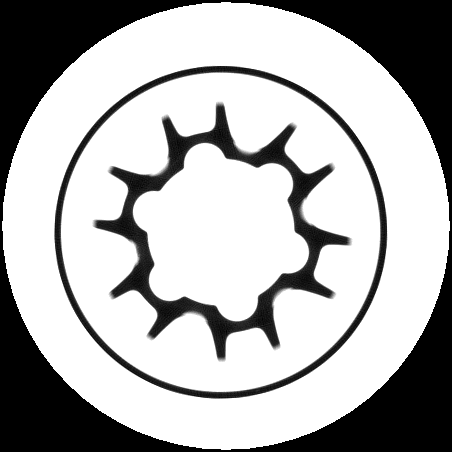

Supplement: S1 File — (ZIP) [file pone.0176383.s001.zip › Raw Image Data of a hub/200503180036.bmp]

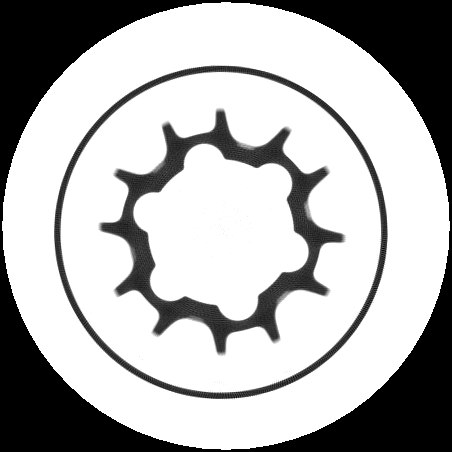

Supplement: S1 File — (ZIP) [file pone.0176383.s001.zip › Raw Image Data of a hub/200503180037.bmp]

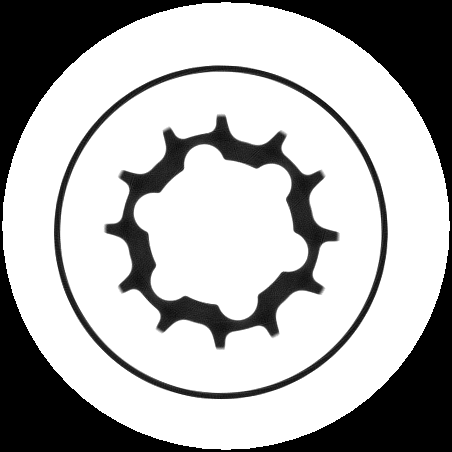

Supplement: S1 File — (ZIP) [file pone.0176383.s001.zip › Raw Image Data of a hub/200503180038.bmp]

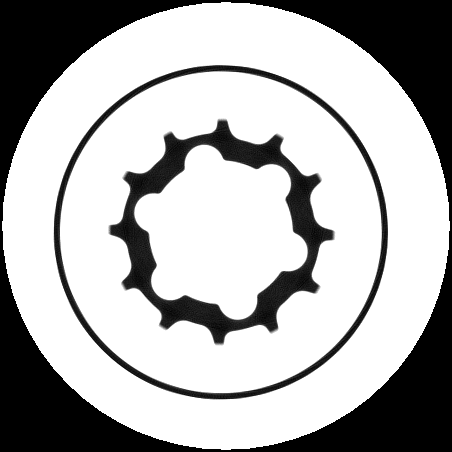

Supplement: S1 File — (ZIP) [file pone.0176383.s001.zip › Raw Image Data of a hub/200503180039.bmp]

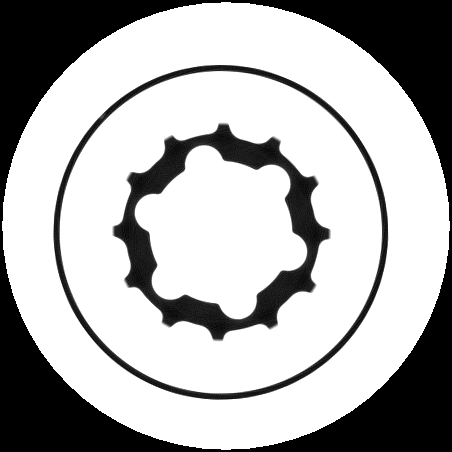

Supplement: S1 File — (ZIP) [file pone.0176383.s001.zip › Raw Image Data of a hub/200503180040.bmp]

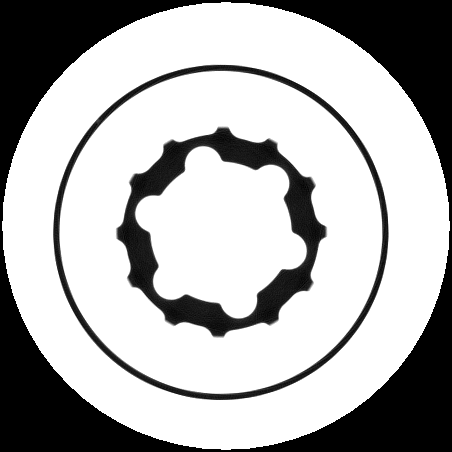

Supplement: S1 File — (ZIP) [file pone.0176383.s001.zip › Raw Image Data of a hub/200503180041.bmp]

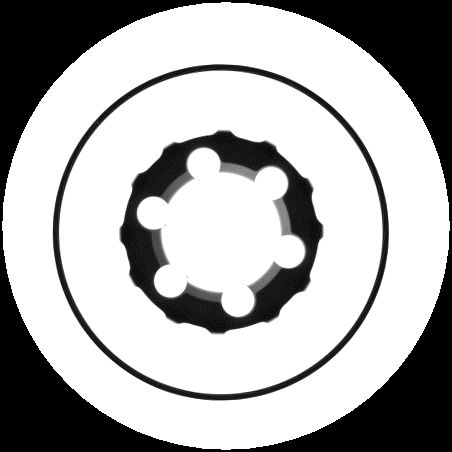

Supplement: S1 File — (ZIP) [file pone.0176383.s001.zip › Raw Image Data of a hub/200503180042.bmp]

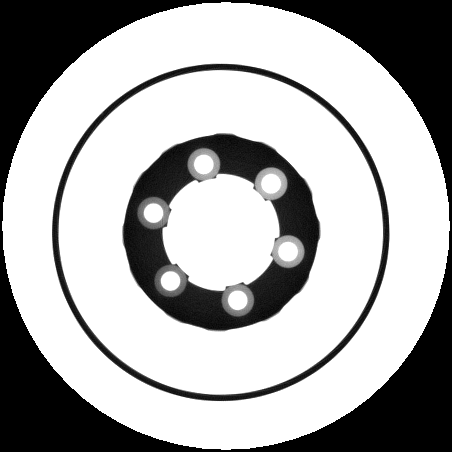

Supplement: S1 File — (ZIP) [file pone.0176383.s001.zip › Raw Image Data of a hub/200503180043.bmp]

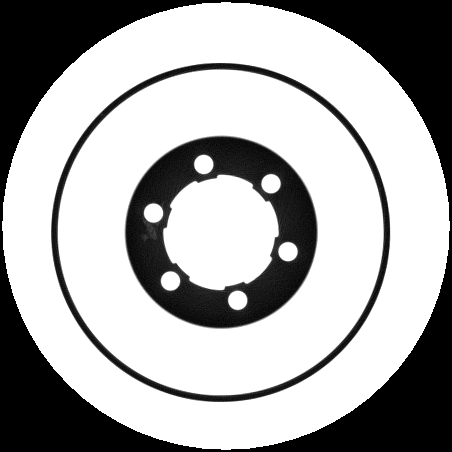

Supplement: S1 File — (ZIP) [file pone.0176383.s001.zip › Raw Image Data of a hub/200503180044.bmp]

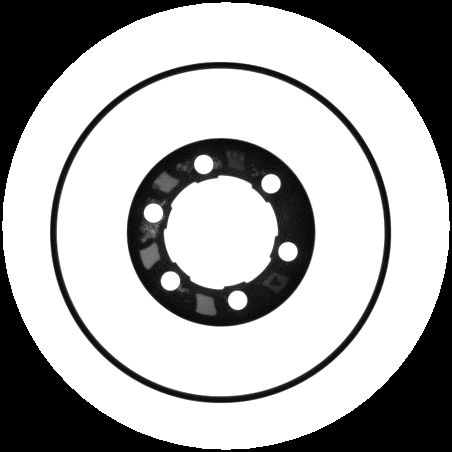

Supplement: S1 File — (ZIP) [file pone.0176383.s001.zip › Raw Image Data of a hub/200503180045.bmp]

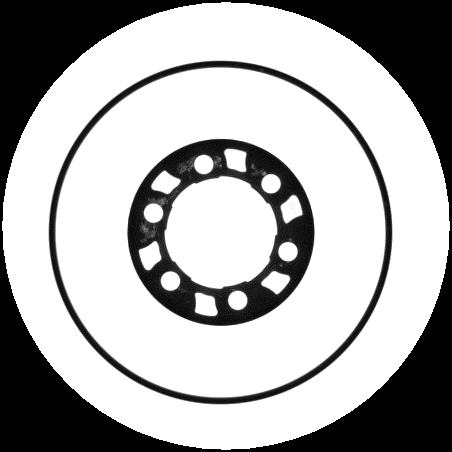

Supplement: S1 File — (ZIP) [file pone.0176383.s001.zip › Raw Image Data of a hub/200503180046.bmp]

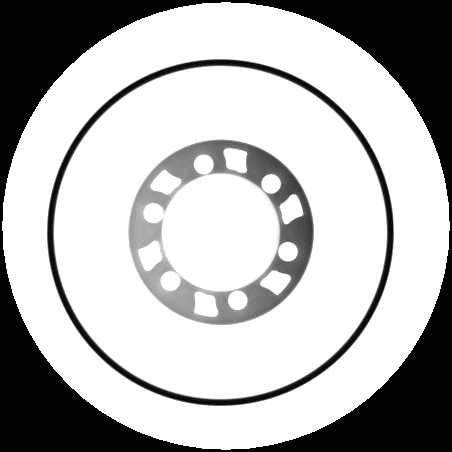

Supplement: S1 File — (ZIP) [file pone.0176383.s001.zip › Raw Image Data of a hub/200503180047.bmp]

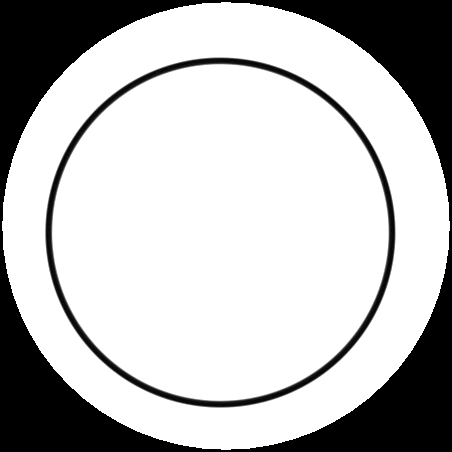

Supplement: S1 File — (ZIP) [file pone.0176383.s001.zip › Raw Image Data of a hub/200503180048.bmp]

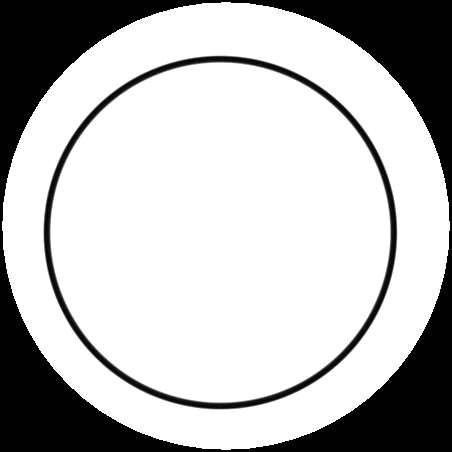

Supplement: S1 File — (ZIP) [file pone.0176383.s001.zip › Raw Image Data of a hub/200503180049.bmp]

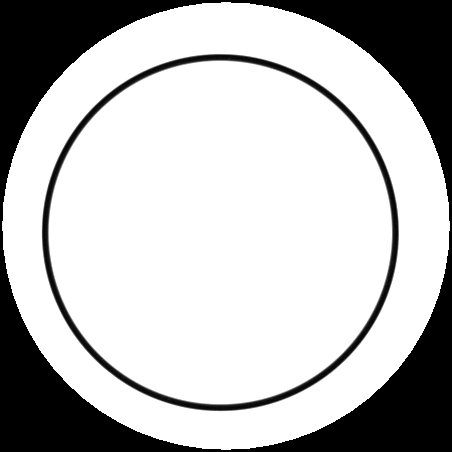

Supplement: S1 File — (ZIP) [file pone.0176383.s001.zip › Raw Image Data of a hub/200503180050.bmp]

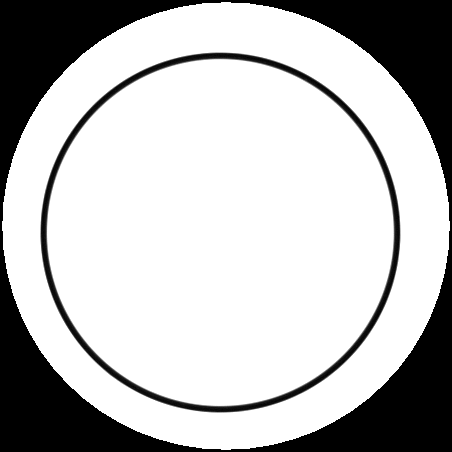

Supplement: S1 File — (ZIP) [file pone.0176383.s001.zip › Raw Image Data of a hub/200503180051.bmp]

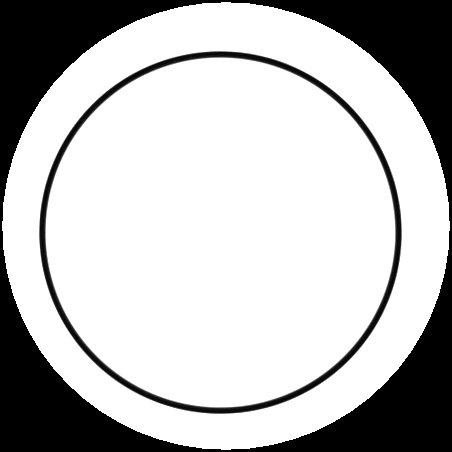

Supplement: S1 File — (ZIP) [file pone.0176383.s001.zip › Raw Image Data of a hub/200503180052.bmp]

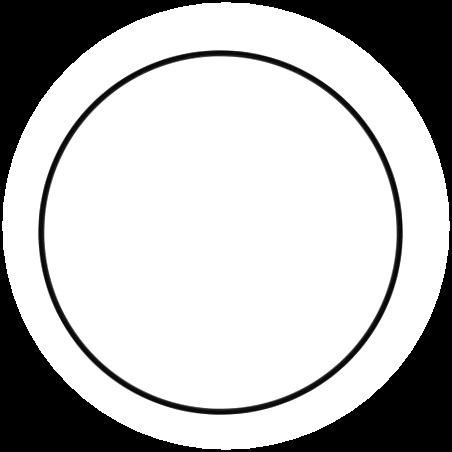

Supplement: S1 File — (ZIP) [file pone.0176383.s001.zip › Raw Image Data of a hub/200503180053.bmp]

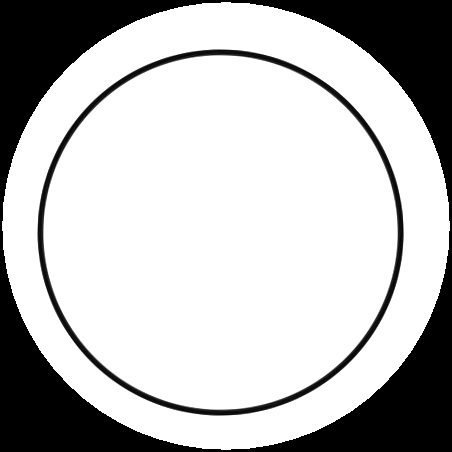

Supplement: S1 File — (ZIP) [file pone.0176383.s001.zip › Raw Image Data of a hub/200503180054.bmp]

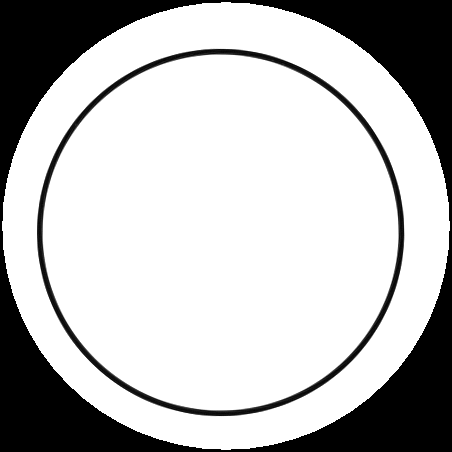

Supplement: S1 File — (ZIP) [file pone.0176383.s001.zip › Raw Image Data of a hub/200503180055.bmp]

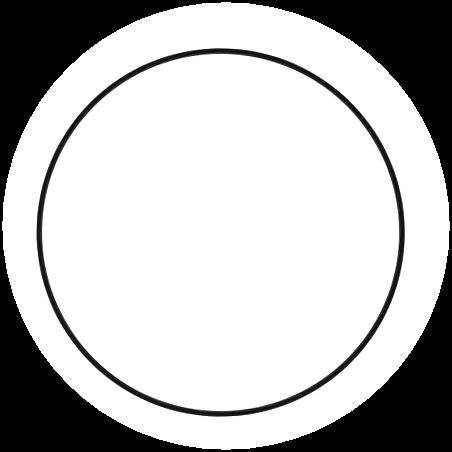

Supplement: S1 File — (ZIP) [file pone.0176383.s001.zip › Raw Image Data of a hub/200503180056.bmp]

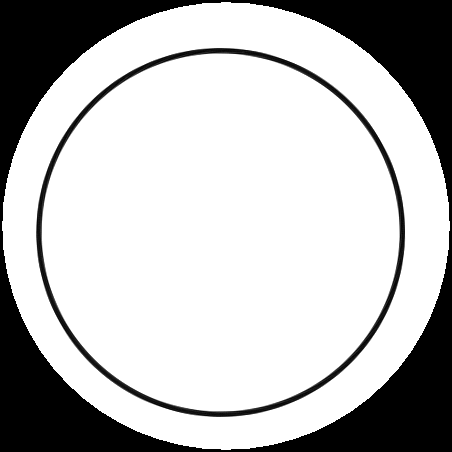

Supplement: S1 File — (ZIP) [file pone.0176383.s001.zip › Raw Image Data of a hub/200503180057.bmp]

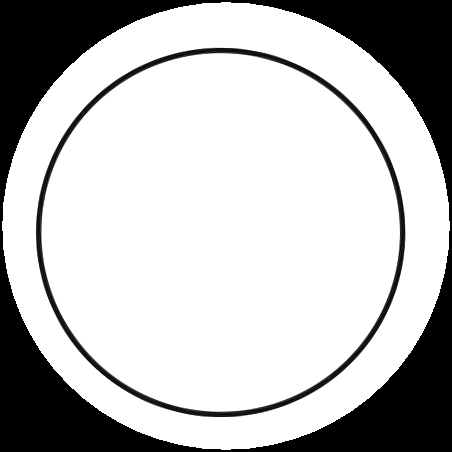

Supplement: S1 File — (ZIP) [file pone.0176383.s001.zip › Raw Image Data of a hub/200503180058.bmp]

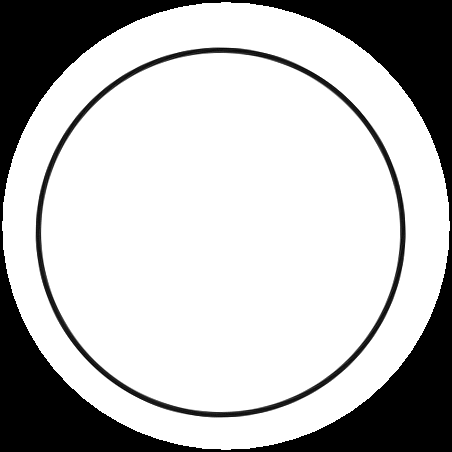

Supplement: S1 File — (ZIP) [file pone.0176383.s001.zip › Raw Image Data of a hub/200503180059.bmp]

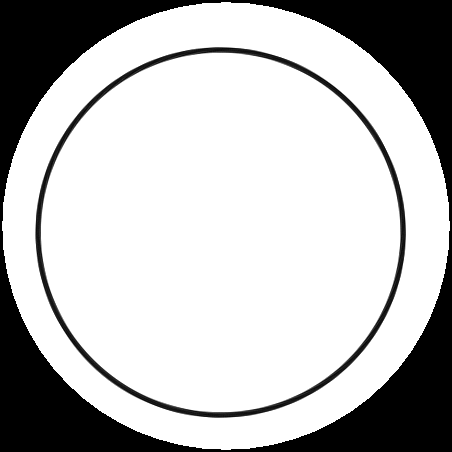

Supplement: S1 File — (ZIP) [file pone.0176383.s001.zip › Raw Image Data of a hub/200503180060.bmp]

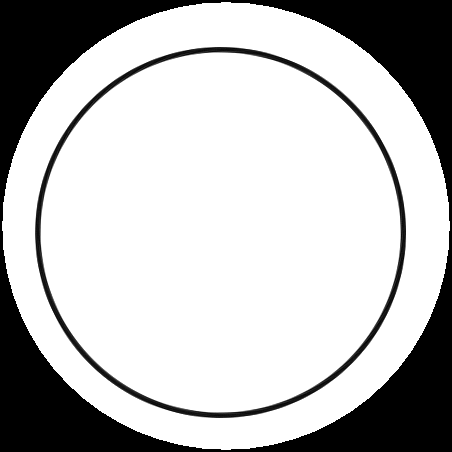

Supplement: S1 File — (ZIP) [file pone.0176383.s001.zip › Raw Image Data of a hub/200503180061.bmp]

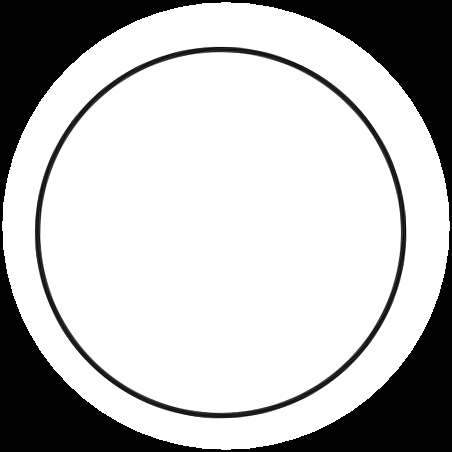

Supplement: S1 File — (ZIP) [file pone.0176383.s001.zip › Raw Image Data of a hub/200503180062.bmp]

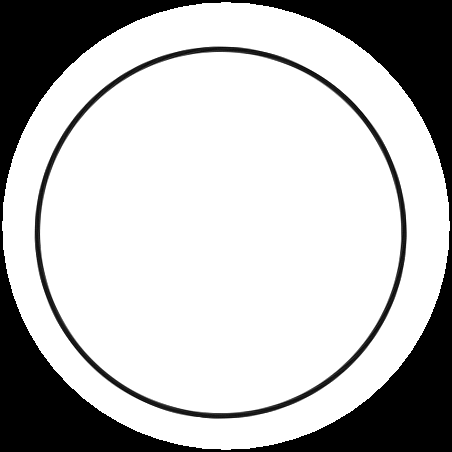

Supplement: S1 File — (ZIP) [file pone.0176383.s001.zip › Raw Image Data of a hub/200503180063.bmp]

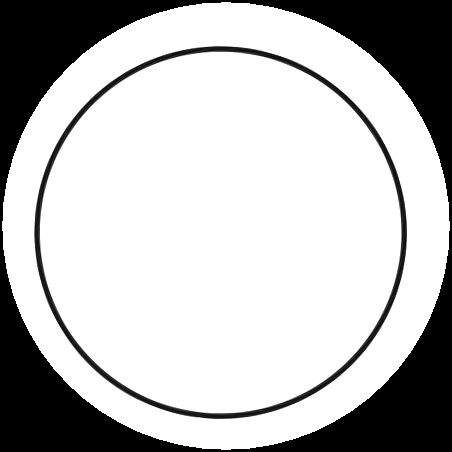

Supplement: S1 File — (ZIP) [file pone.0176383.s001.zip › Raw Image Data of a hub/200503180064.bmp]

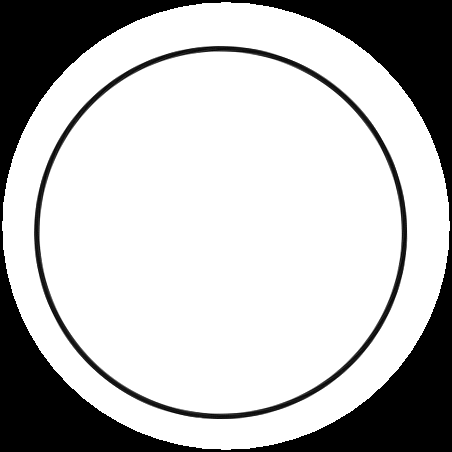

Supplement: S1 File — (ZIP) [file pone.0176383.s001.zip › Raw Image Data of a hub/200503180065.bmp]

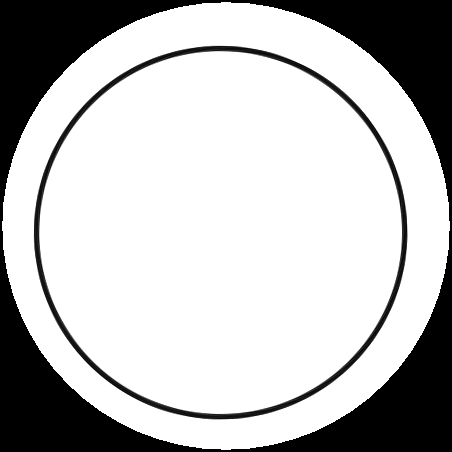

Supplement: S1 File — (ZIP) [file pone.0176383.s001.zip › Raw Image Data of a hub/200503180066.bmp]

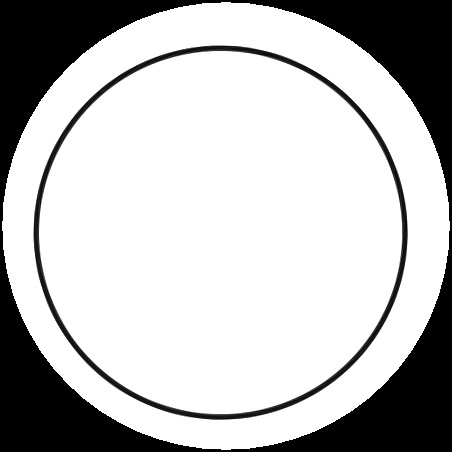

Supplement: S1 File — (ZIP) [file pone.0176383.s001.zip › Raw Image Data of a hub/200503180067.bmp]

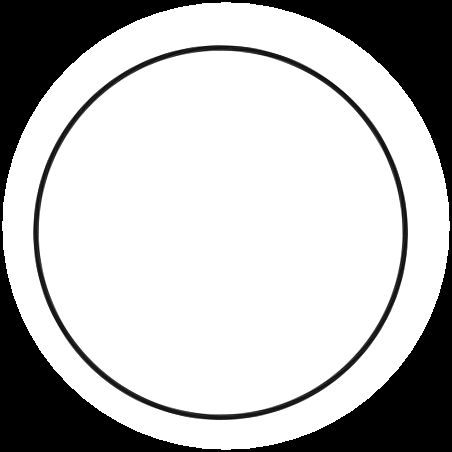

Supplement: S1 File — (ZIP) [file pone.0176383.s001.zip › Raw Image Data of a hub/200503180068.bmp]

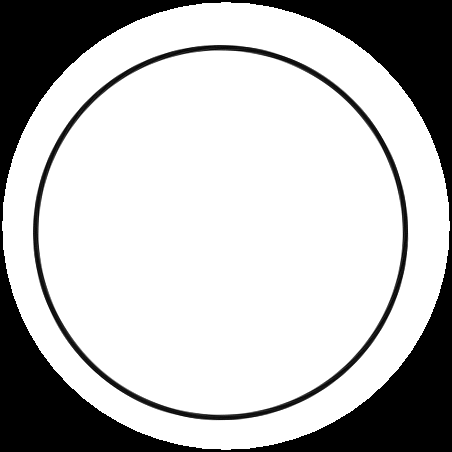

Supplement: S1 File — (ZIP) [file pone.0176383.s001.zip › Raw Image Data of a hub/200503180069.bmp]

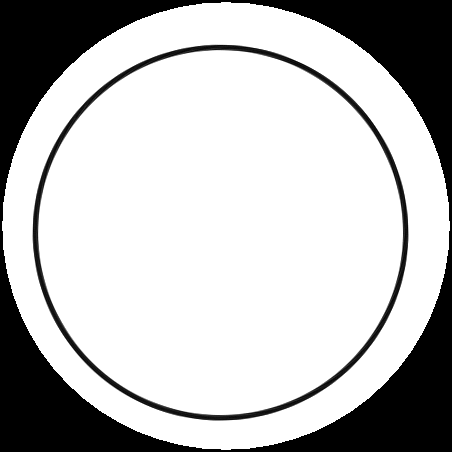

Supplement: S1 File — (ZIP) [file pone.0176383.s001.zip › Raw Image Data of a hub/200503180070.bmp]

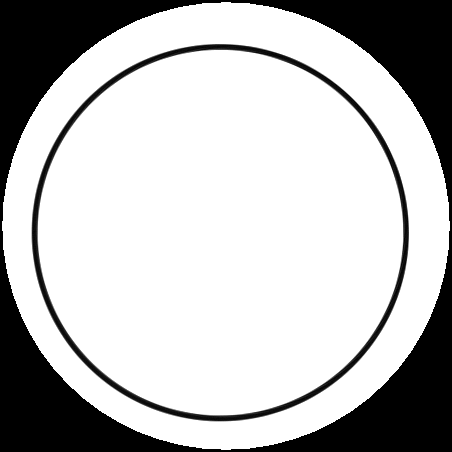

Supplement: S1 File — (ZIP) [file pone.0176383.s001.zip › Raw Image Data of a hub/200503180071.bmp]

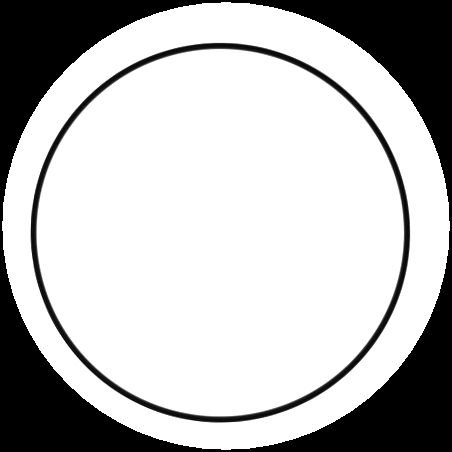

Supplement: S1 File — (ZIP) [file pone.0176383.s001.zip › Raw Image Data of a hub/200503180072.bmp]

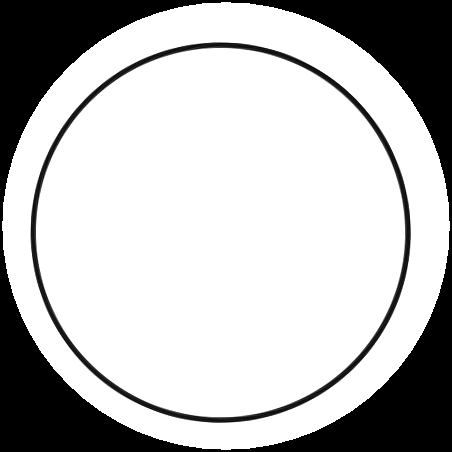

Supplement: S1 File — (ZIP) [file pone.0176383.s001.zip › Raw Image Data of a hub/200503180073.bmp]

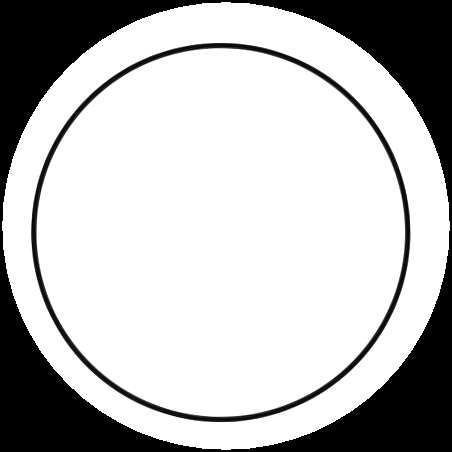

Supplement: S1 File — (ZIP) [file pone.0176383.s001.zip › Raw Image Data of a hub/200503180074.bmp]

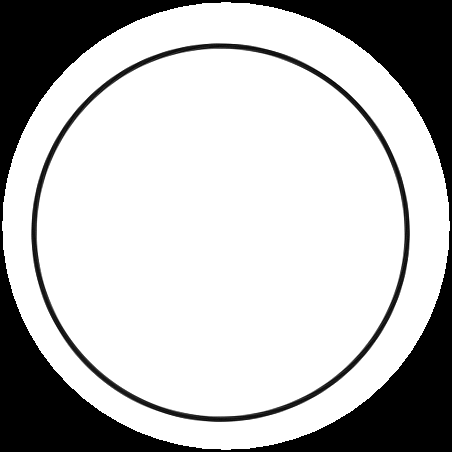

Supplement: S1 File — (ZIP) [file pone.0176383.s001.zip › Raw Image Data of a hub/200503180075.bmp]

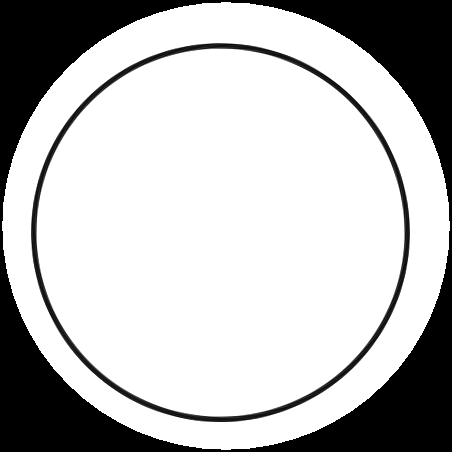

Supplement: S1 File — (ZIP) [file pone.0176383.s001.zip › Raw Image Data of a hub/200503180076.bmp]

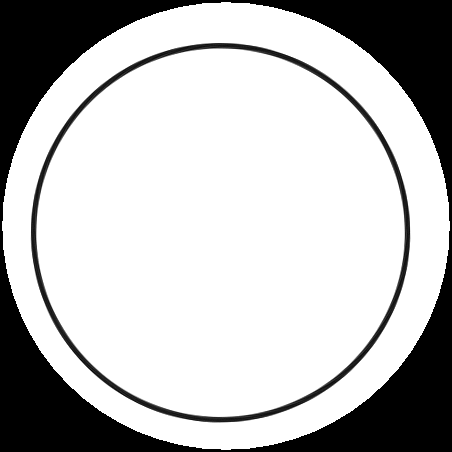

Supplement: S1 File — (ZIP) [file pone.0176383.s001.zip › Raw Image Data of a hub/200503180077.bmp]

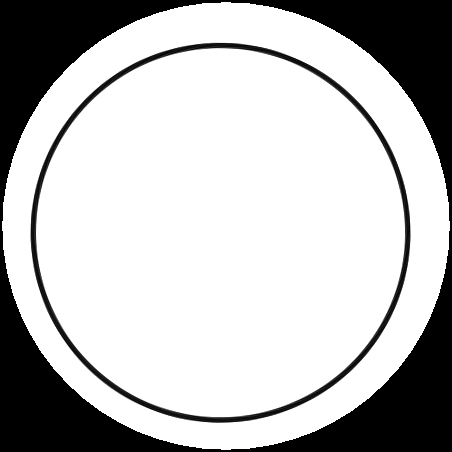

Supplement: S1 File — (ZIP) [file pone.0176383.s001.zip › Raw Image Data of a hub/200503180078.bmp]

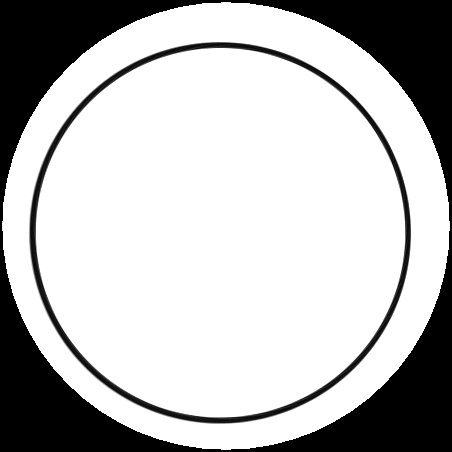

Supplement: S1 File — (ZIP) [file pone.0176383.s001.zip › Raw Image Data of a hub/200503180079.bmp]

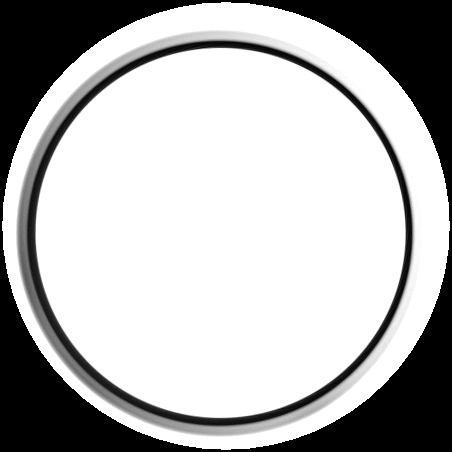

Supplement: S1 File — (ZIP) [file pone.0176383.s001.zip › Raw Image Data of a hub/200503180080.bmp]

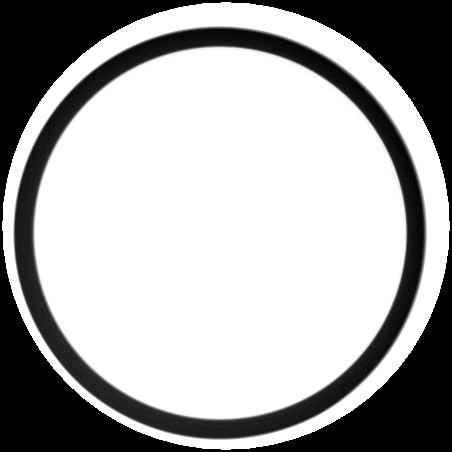

Supplement: S1 File — (ZIP) [file pone.0176383.s001.zip › Raw Image Data of a hub/200503180081.bmp]

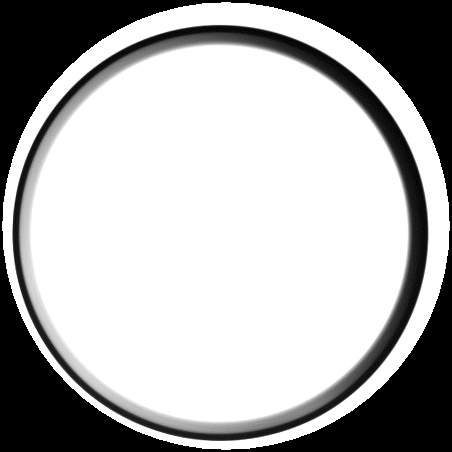

Supplement: S1 File — (ZIP) [file pone.0176383.s001.zip › Raw Image Data of a hub/200503180082.bmp]

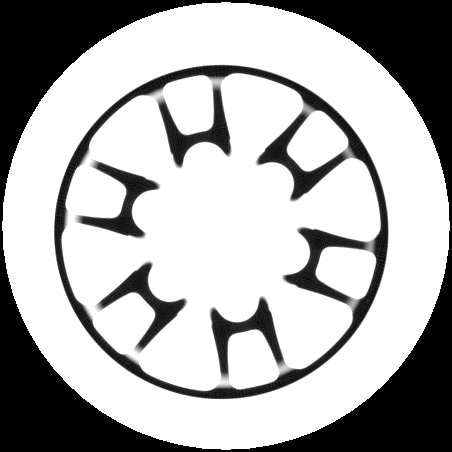

Supplement: S1 File — (ZIP) [file pone.0176383.s001.zip › Raw Image Data of a hub/200503180034.bmp]

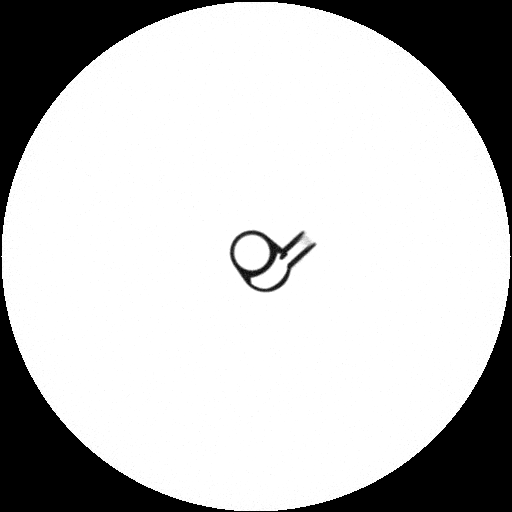

Supplement: S1 File — (ZIP) [file pone.0176383.s001.zip › Raw Image Data of a carburetor/CC-23.bmp]

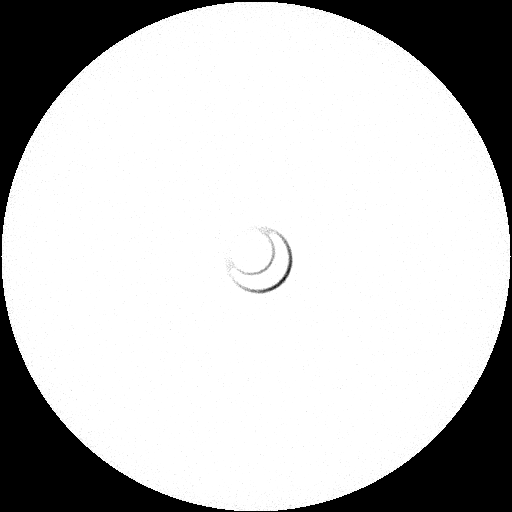

Supplement: S1 File — (ZIP) [file pone.0176383.s001.zip › Raw Image Data of a carburetor/CC-1.bmp]

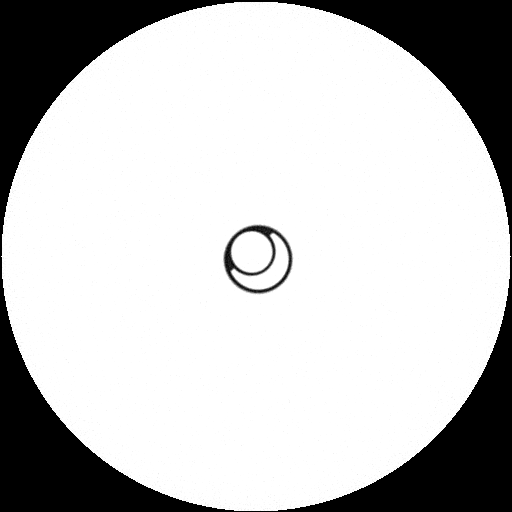

Supplement: S1 File — (ZIP) [file pone.0176383.s001.zip › Raw Image Data of a carburetor/CC-10.bmp]

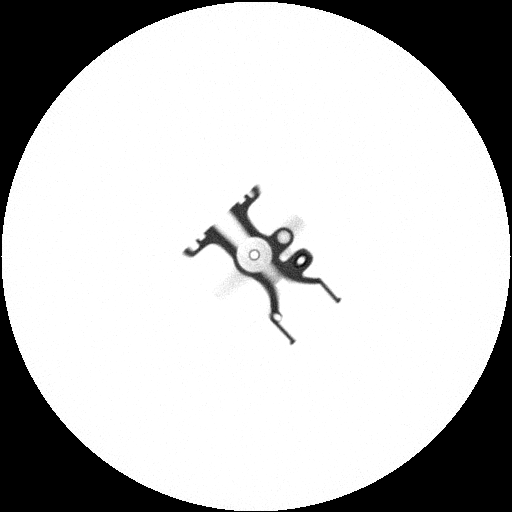

Supplement: S1 File — (ZIP) [file pone.0176383.s001.zip › Raw Image Data of a carburetor/CC-100.bmp]

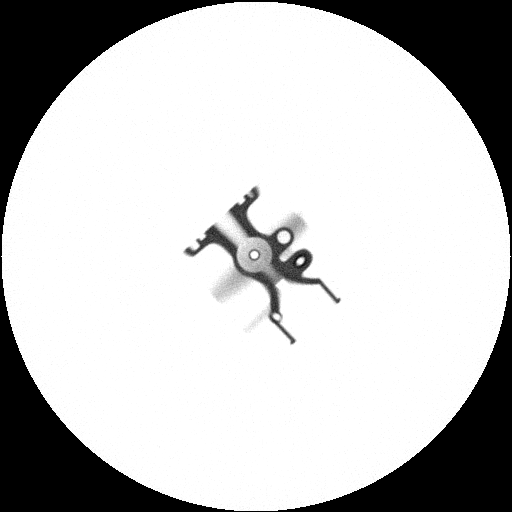

Supplement: S1 File — (ZIP) [file pone.0176383.s001.zip › Raw Image Data of a carburetor/CC-101.bmp]

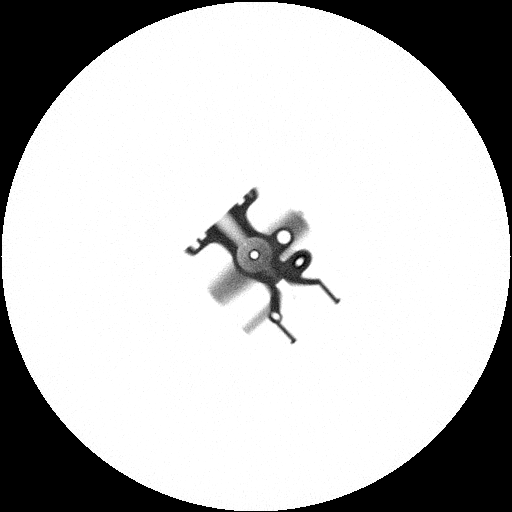

Supplement: S1 File — (ZIP) [file pone.0176383.s001.zip › Raw Image Data of a carburetor/CC-102.bmp]

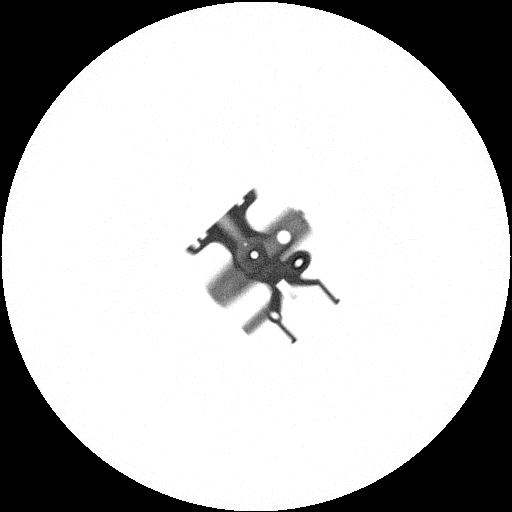

Supplement: S1 File — (ZIP) [file pone.0176383.s001.zip › Raw Image Data of a carburetor/CC-103.bmp]

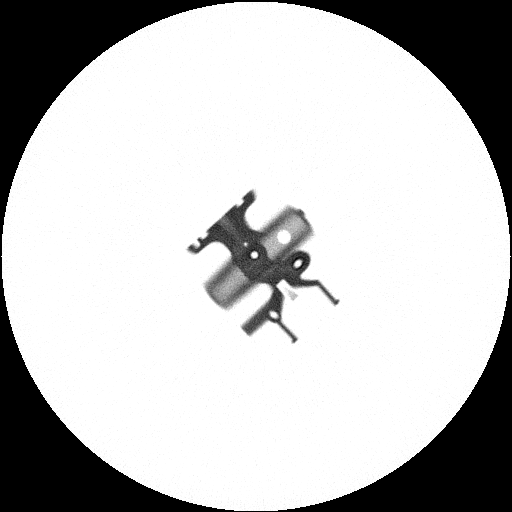

Supplement: S1 File — (ZIP) [file pone.0176383.s001.zip › Raw Image Data of a carburetor/CC-104.bmp]

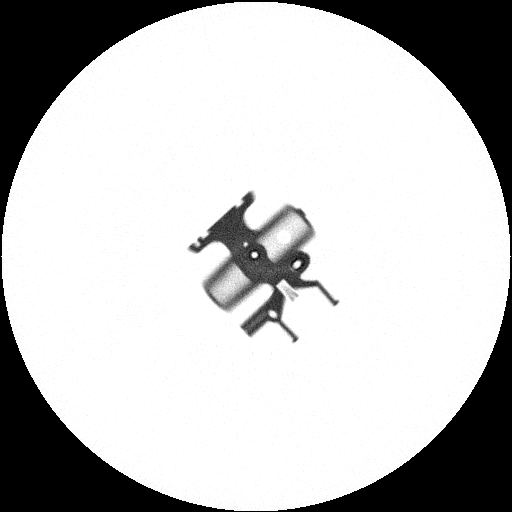

Supplement: S1 File — (ZIP) [file pone.0176383.s001.zip › Raw Image Data of a carburetor/CC-105.bmp]

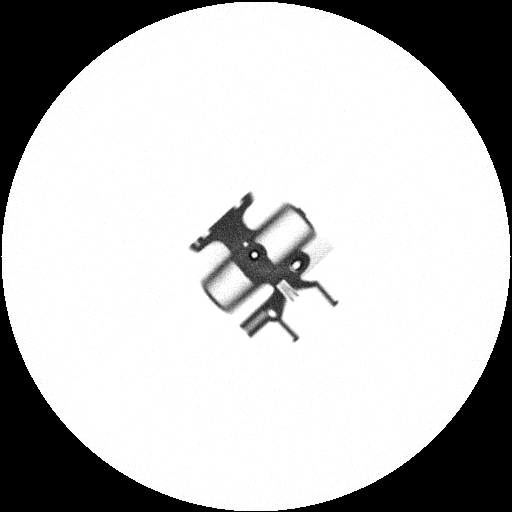

Supplement: S1 File — (ZIP) [file pone.0176383.s001.zip › Raw Image Data of a carburetor/CC-106.bmp]

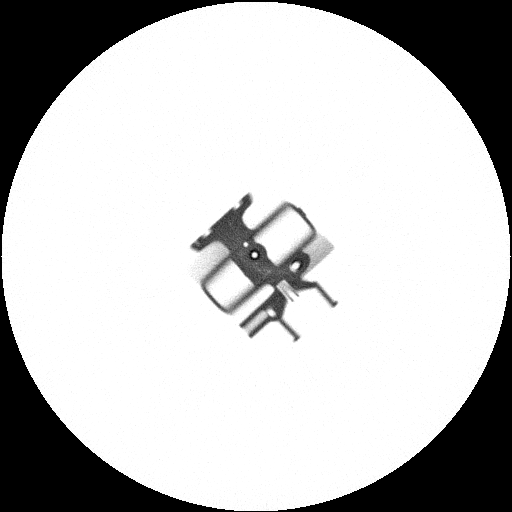

Supplement: S1 File — (ZIP) [file pone.0176383.s001.zip › Raw Image Data of a carburetor/CC-107.bmp]

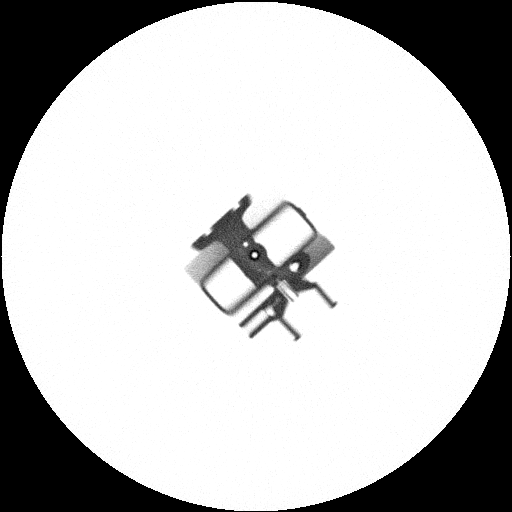

Supplement: S1 File — (ZIP) [file pone.0176383.s001.zip › Raw Image Data of a carburetor/CC-108.bmp]

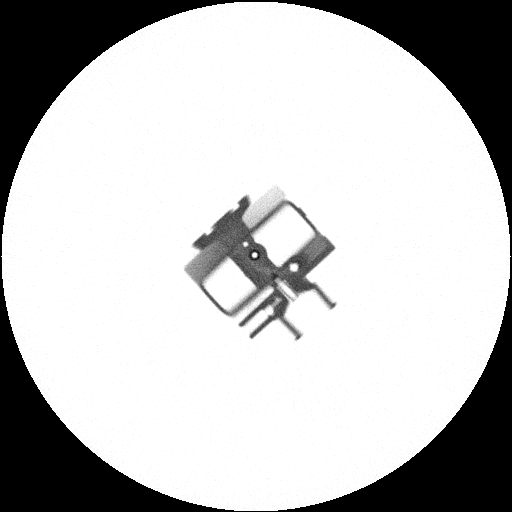

Supplement: S1 File — (ZIP) [file pone.0176383.s001.zip › Raw Image Data of a carburetor/CC-109.bmp]

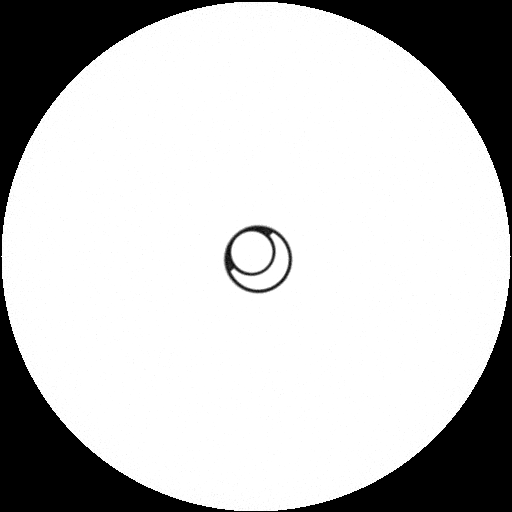

Supplement: S1 File — (ZIP) [file pone.0176383.s001.zip › Raw Image Data of a carburetor/CC-11.bmp]

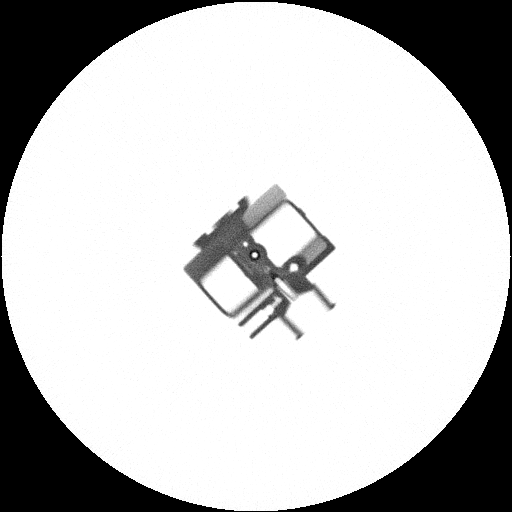

Supplement: S1 File — (ZIP) [file pone.0176383.s001.zip › Raw Image Data of a carburetor/CC-110.bmp]

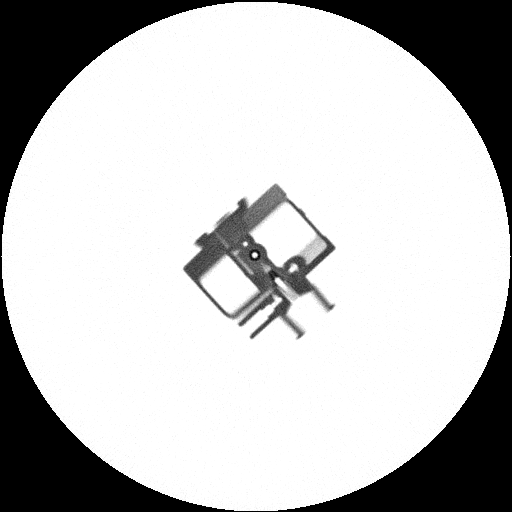

Supplement: S1 File — (ZIP) [file pone.0176383.s001.zip › Raw Image Data of a carburetor/CC-111.bmp]

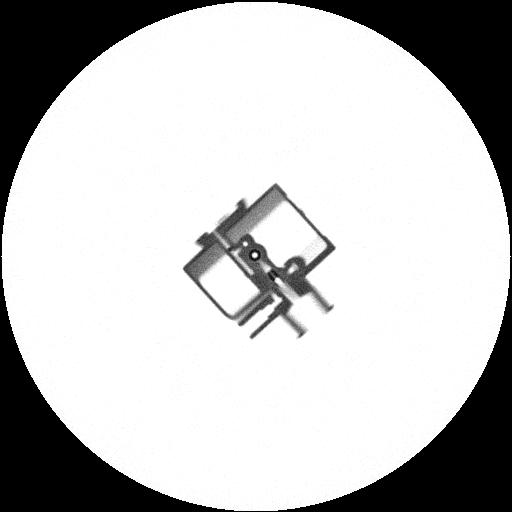

Supplement: S1 File — (ZIP) [file pone.0176383.s001.zip › Raw Image Data of a carburetor/CC-112.bmp]

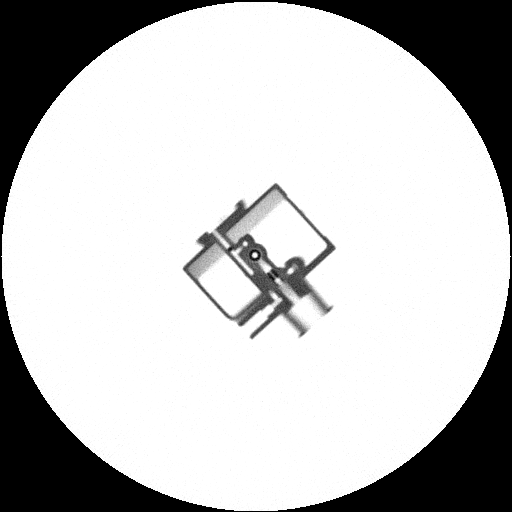

Supplement: S1 File — (ZIP) [file pone.0176383.s001.zip › Raw Image Data of a carburetor/CC-113.bmp]

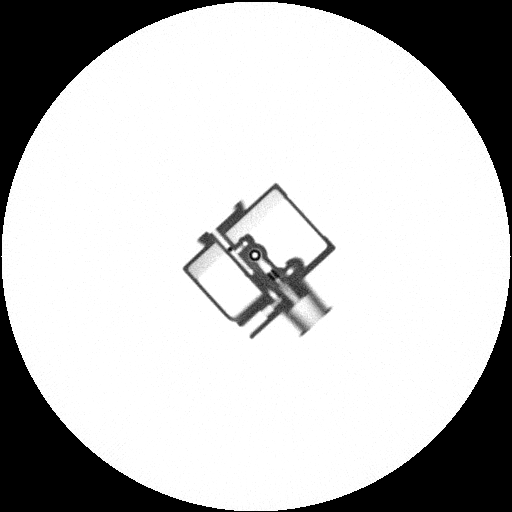

Supplement: S1 File — (ZIP) [file pone.0176383.s001.zip › Raw Image Data of a carburetor/CC-114.bmp]

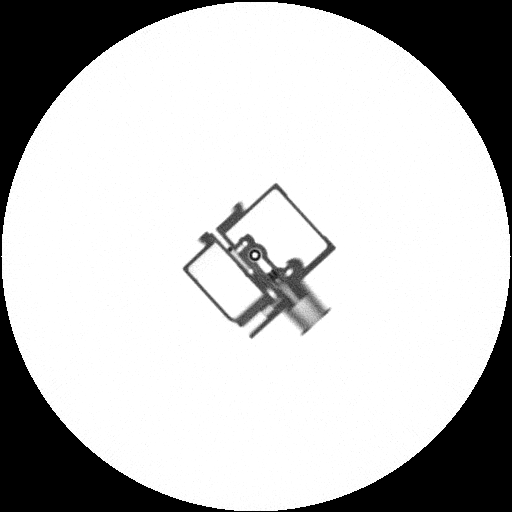

Supplement: S1 File — (ZIP) [file pone.0176383.s001.zip › Raw Image Data of a carburetor/CC-115.bmp]

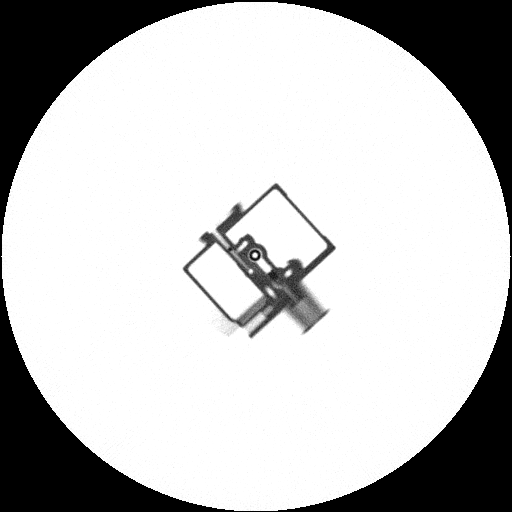

Supplement: S1 File — (ZIP) [file pone.0176383.s001.zip › Raw Image Data of a carburetor/CC-116.bmp]

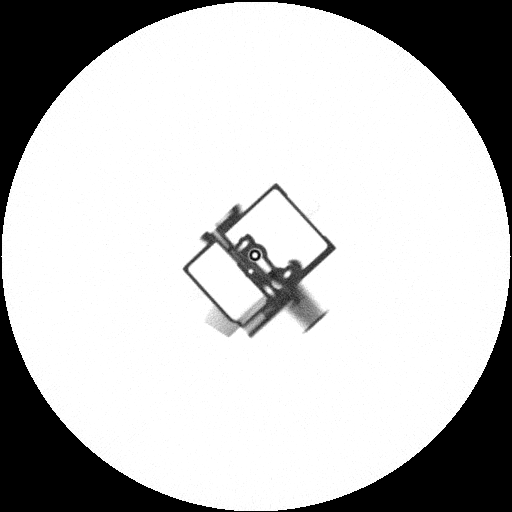

Supplement: S1 File — (ZIP) [file pone.0176383.s001.zip › Raw Image Data of a carburetor/CC-117.bmp]

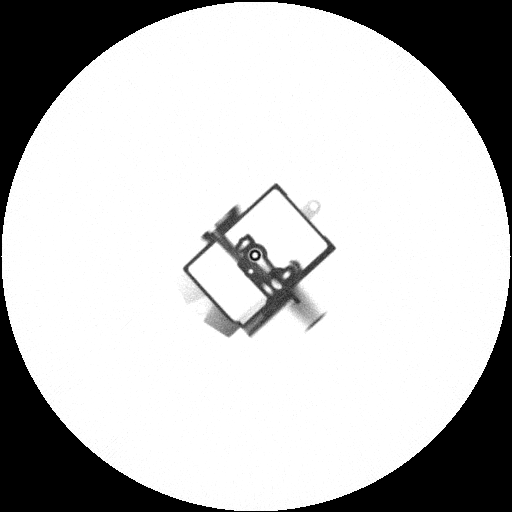

Supplement: S1 File — (ZIP) [file pone.0176383.s001.zip › Raw Image Data of a carburetor/CC-118.bmp]

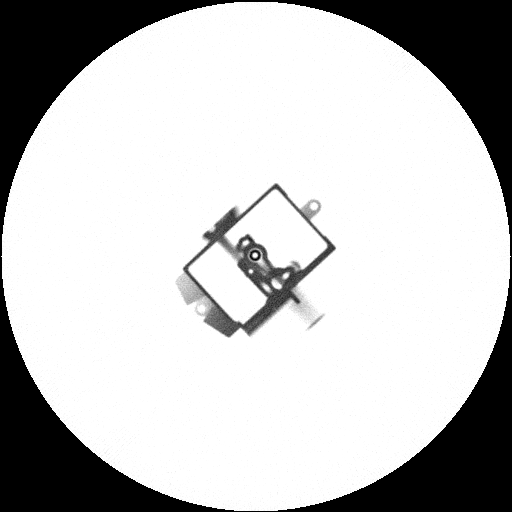

Supplement: S1 File — (ZIP) [file pone.0176383.s001.zip › Raw Image Data of a carburetor/CC-119.bmp]

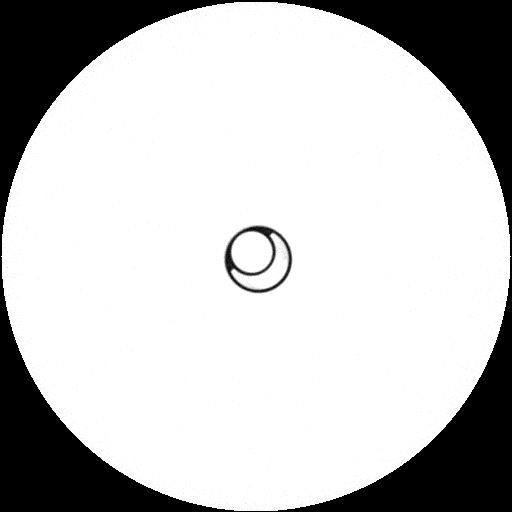

Supplement: S1 File — (ZIP) [file pone.0176383.s001.zip › Raw Image Data of a carburetor/CC-12.bmp]

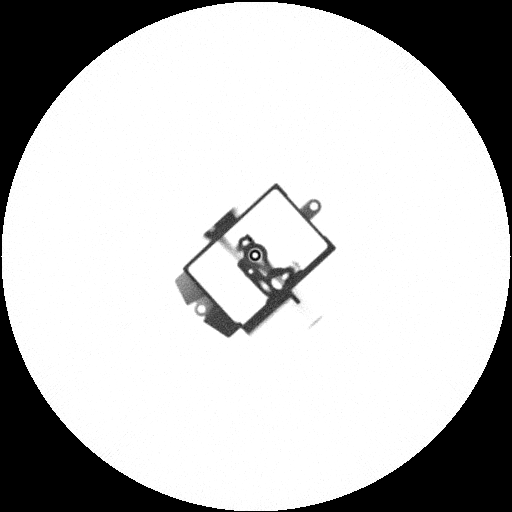

Supplement: S1 File — (ZIP) [file pone.0176383.s001.zip › Raw Image Data of a carburetor/CC-120.bmp]

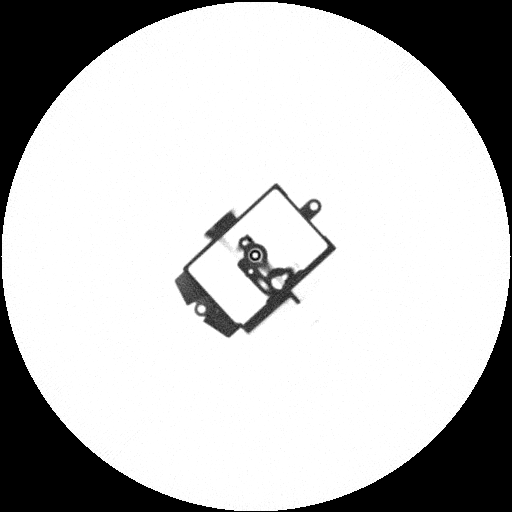

Supplement: S1 File — (ZIP) [file pone.0176383.s001.zip › Raw Image Data of a carburetor/CC-121.bmp]

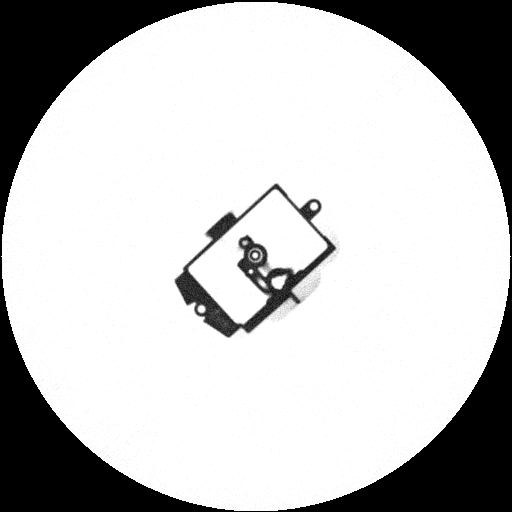

Supplement: S1 File — (ZIP) [file pone.0176383.s001.zip › Raw Image Data of a carburetor/CC-122.bmp]

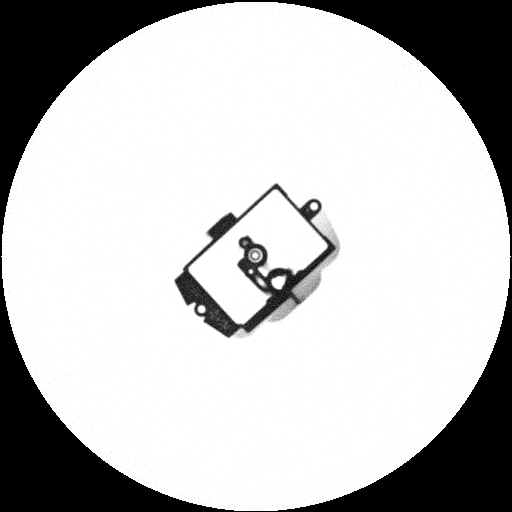

Supplement: S1 File — (ZIP) [file pone.0176383.s001.zip › Raw Image Data of a carburetor/CC-123.bmp]

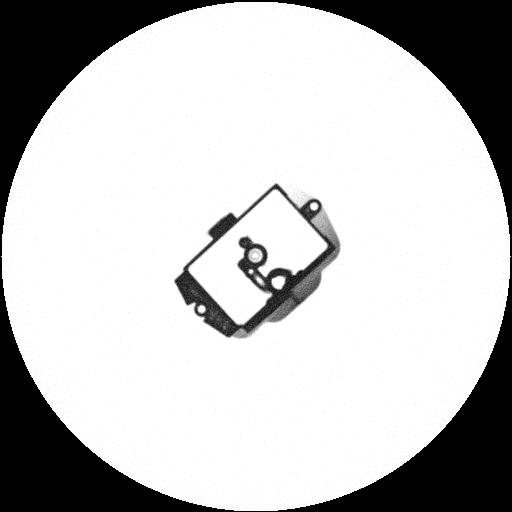

Supplement: S1 File — (ZIP) [file pone.0176383.s001.zip › Raw Image Data of a carburetor/CC-124.bmp]

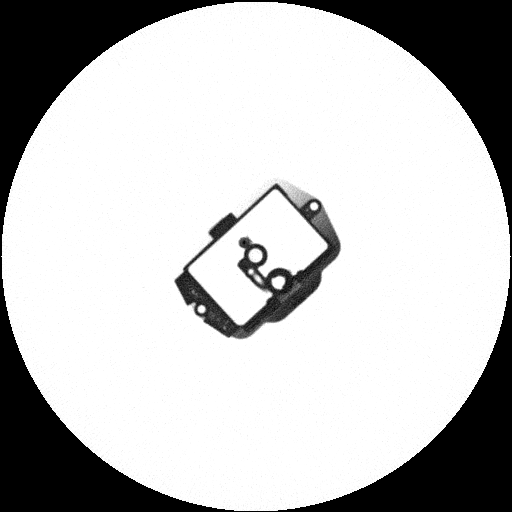

Supplement: S1 File — (ZIP) [file pone.0176383.s001.zip › Raw Image Data of a carburetor/CC-125.bmp]

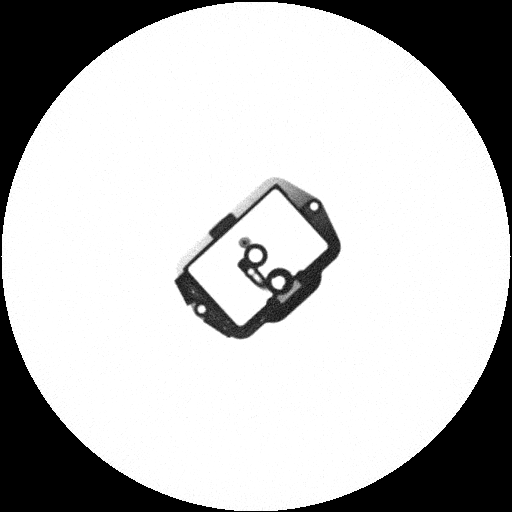

Supplement: S1 File — (ZIP) [file pone.0176383.s001.zip › Raw Image Data of a carburetor/CC-126.bmp]

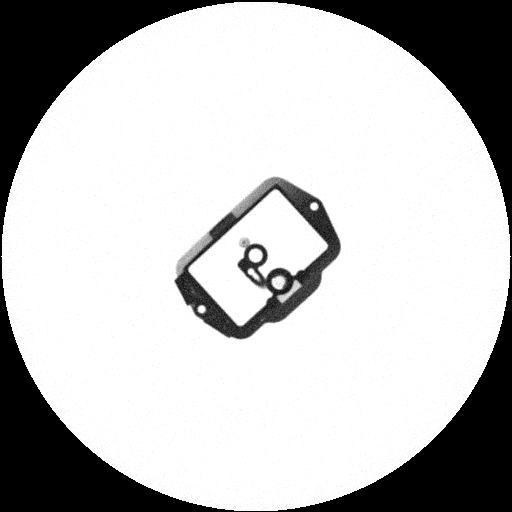

Supplement: S1 File — (ZIP) [file pone.0176383.s001.zip › Raw Image Data of a carburetor/CC-127.bmp]
